# Based on network pharmacology and bioinformatics to analyze the mechanism of action of Astragalus membranaceus in the treatment of vitiligo and COVID-19

**DOI:** 10.1038/s41598-023-29207-6

**Published:** 2023-03-08

**Authors:** Yaojun Wang, Ming Ding, Jiaoni Chi, Tao Wang, Yue Zhang, Zhimin Li, Qiang Li

**Affiliations:** 1 Graduate School, Hebei North University, Zhangjiakou, 075000 China; 2 Air Force Clinical College, The Fifth School of Clinical Medicine, Anhui Medical University, Hefei, 230032 China; 3 Department of Dermatology, Air Force Medical Center, PLA, Beijing, 100142 China

**Keywords:** Vitiligo, Pharmacology, Pharmacodynamics

## Abstract

Coronavirus disease 2019 (COVID-19) is spreading rapidly around the world. However, the treatment of vitiligo combined with COVID-19 has not been reported. Astragalus membranaceus (AM) has a therapeutic effect on patients with vitiligo and COVID-19. This study aims to discover its possible therapeutic mechanisms and provide potential drug targets. Using the Chinese Medicine System Pharmacological Database (TCMSP), GEO database and Genecards websites and other databases, AM target, vitiligo disease target, and COVID-19 related gene set were established. Then find the crossover genes by taking the intersection. Then use GO, KEGG enrichment analysis, and PPI network to discover its underlying mechanism. Finally, by importing drugs, active ingredients, crossover genes, and enriched signal pathways into Cytoscape software, a “drug-active ingredient-target signal pathway-” network is constructed. TCMSP screened and obtained 33 active ingredients including baicalein (MOL002714), NEOBAICALEIN (MOL002934), Skullcapflavone II (MOL002927), and wogonin (MOL000173), which acted on 448 potential targets. 1166 differentially expressed genes for vitiligo were screened by GEO. CIVID-19 related genes were screened by Genecards. Then by taking the intersection, a total of 10 crossover genes (PTGS2, CDK1, STAT1, BCL2L1, SCARB1, HIF1A, NAE1, PLA2G4A, HSP90AA1, and HSP90B1) were obtained. KEGG analysis found that it was mainly enriched in signaling pathways such as IL-17 signaling pathway, Th17 cell differentiation, Necroptosis, NOD-like receptor signaling pathway. Five core targets (PTGS2, STAT1, BCL2L1, HIF1A, and HSP90AA1) were obtained by analyzing the PPI network. The network of "active ingredients-crossover genes" was constructed by Cytoscape, and the 5 main active ingredients acting on the 5 core crossover genes acacetin, wogonin, baicalein, bis2S)-2-ethylhexyl) benzene-1,2-dicarboxylate and 5,2′-Dihydroxy-6,7,8-trimethoxyflavone. The core crossover genes obtained by PPI and the core crossover genes obtained by the "active ingredient-crossover gene" network are intersected to obtain the three most important core genes (PTGS2, STAT1, HSP90AA1). AM may act on PTGS2, STAT1, HSP90AA1, etc. through active components such as acacetin, wogonin, baicalein, bis2S)-2-ethylhexyl) benzene-1,2-dicarboxylate and 5,2′-Dihydroxy-6,7,8-trimethoxyflavone to activate IL-17 signaling pathway, Th17 cell differentiation, Necroptosis, NOD-like receptor signaling pathway, Kaposi sarcoma-associated herpesvirus infection, and VEGF signaling pathway and other signaling pathways to achieve the effect of treating vitiligo and COVID-19.

## Introduction

Vitiligo is a depigmented autoimmune skin disease that affects between 0.1 and 2% of the global population. It can happen to anyone, at any age, in any location, and in any size. The persistent decline or disappearance of melanocytes in skin sores is the most obsessive feature of vitiligo. Its pathogenesis is unknown, but it is currently thought to be linked to heredity, oxidative stress, autoimmunity, melanocyte autophagy, development factors, profound elements, etc.^1^. Current clinical treatment is essentially centered around improving the melanogenesis of melanocytes^2^. Because of the inability to follow the pathogenesis of vitiligo, its application is restricted. Traditional Chinese Medicine (TCM) is an extensive clinical framework that assumes a key part in keeping up with the soundness of Asian populaces. Due to the relatively few curative effects and side effects of traditional Chinese medicine, it has gradually become popular in Western countries. According to the medical theory of traditional Chinese medicine, traditional Chinese medicine is considered to be a good source of prevention and treatment of complex diseases such as vitiligo^3^. Some studies have demonstrated that AM has therapeutic effects in both vitiligo and COVID-19 patients. However, little is currently known about the common mechanisms of action and their potential targets in vitiligo patients with COVID-19 disease. Consequently, it is essential to explain the specific mechanism of AM in these diseases.

COVID-19 is an acute respiratory infectious disease caused by severe acute respiratory syndrome coronavirus-2 (SARS-CoV-2). It can cause fever, fatigue, dry cough, multiple symptoms, and all organ dysfunction and death^4^. Notwithstanding, drugs that can treat SARS-CoV-2 disease are as yet slippery. The high cost and adverse side effects of synthetic drugs, as well as the emergence of adverse reactions, require safe and novel antiviral drugs. In the treatment process, TCM has also made a huge contribution^5^. A meta-analysis involving 11 studies compared traditional Chinese medicine plus Western medicine with pure Western medicine^6^. The summary results showed that the combination of Chinese and Western medicine produced a higher remission rate, a high cure rate, a low severity rate, and a short hospital stay. However, the mechanism of action of TCM is still unclear because TCM is usually composed of dozens of compounds. Exploring the active compounds and target genes of traditional Chinese medicine has clinical significance for guiding drug discovery.

**Figure 1:**
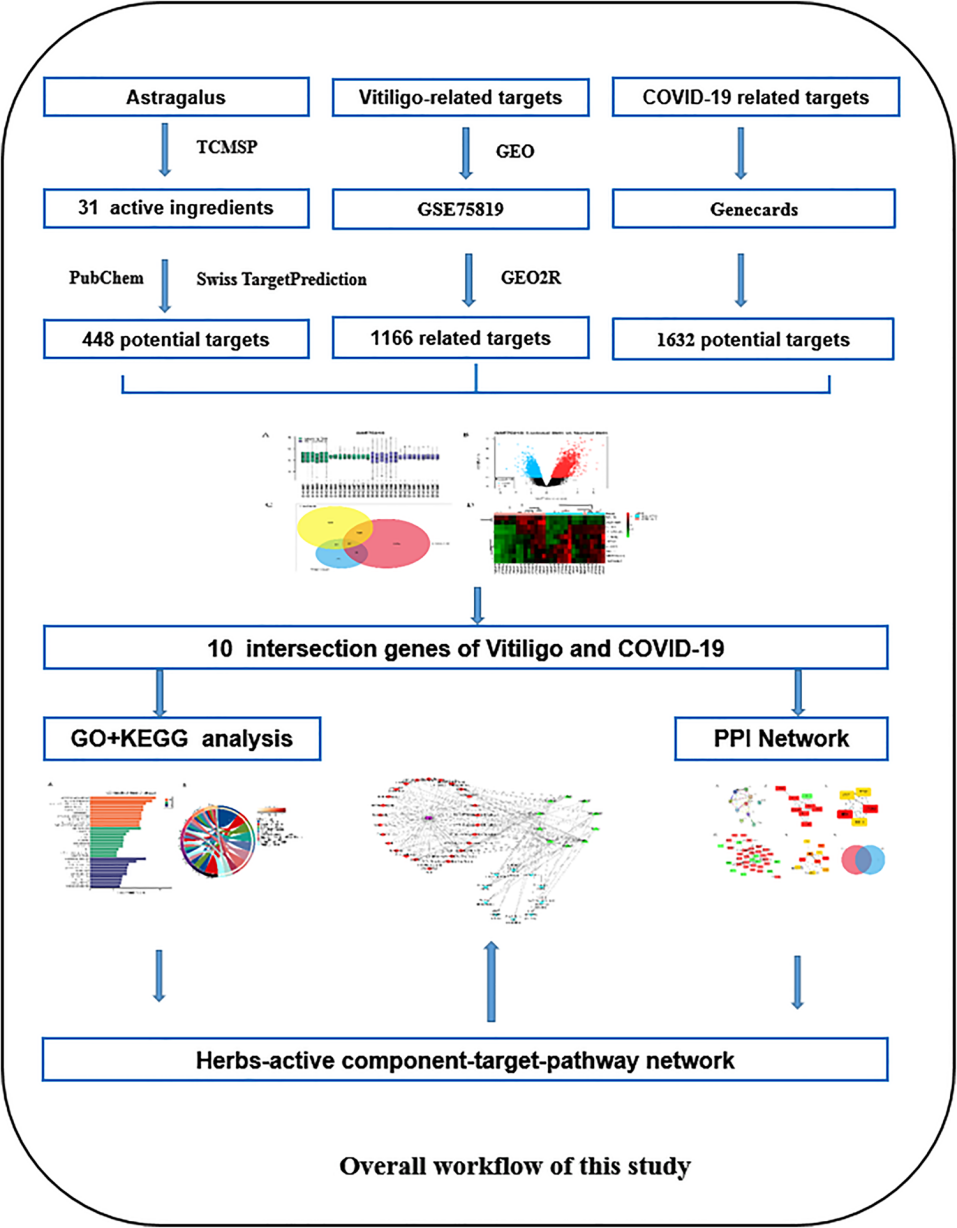
Overall workflow of this study.

AM, known as HuangQi in China. It is the dried root of astragalus (Fisch).AM is a medicinal plant of the family Leguminosae, species Astragalus, genus Astragalus. Herbal medicine has been shown to have immunomodulatory, anti-hyperglycemic, anti-inflammatory, antioxidant, and antiviral activities^7,8^. Lin et al.^8,9^ observed that the water extract of astragalus root can stimulate the proliferation of melanocytes. Astragaloside IV (ASIV) is the main active ingredient isolated from the Astragalus root. Some studies have reported its pharmacological effects, including anti-oxidation, anti-inflammatory, anti-fungal, and anti-hair loss^10^. Research by Adhikari et al.^11^ has shown that crude extracts or pure compounds extracted from the medicinal plant AM have shown inhibitory effects on the coronavirus. In another clinical trial of 82 patients with acute exacerbation of COPD chronic obstructive pulmonary disease, in conventional bronchodilator treatment, 15 mg of Huangqi granules twice a day for two consecutive weeks can significantly reduce TNF-α and IL- 8. IL-1-β and IL-32^12^. However, the molecular mechanism of AM in the treatment of vitiligo and COVID-19 is still unclear. Therefore, this study is planned to be based on network pharmacology and bioinformatics methods to systematically analyze the main active components of AM and its targets and pathways in the treatment of vitiligo and COVID-19. Analysis, to provide a reference for follow-up research. The detailed process is shown in the flowchart Fig. 1.

## Materials and methods

### Collection of active ingredients of AM and screening of active ingredients

In order to study the main active ingredients of AM, the traditional Chinese medicine system TCMSP^13^, (http://tcmspw.com/tcmsp.php/). The pharmacological database and analysis platform collect the main active ingredients of AM. The pharmacokinetic parameters of oral bioavailability (OB) ≥ 30% and drug-likeness (DL) ≥ 0.18 were used as criteria to screen the major active ingredients of AM as candidate compounds^14^. The "drug-active ingredient" network was constructed using Cytoscape 3.8.2 software.

### Prediction of the target of the active ingredients of AM

The active ingredients obtained from screening were entered into the PubChem (https://pubchem.ncbi.nlm.nih.gov/) using their CAS number or InChIKey to acquire the corresponding molecular structures which were stored in canonical simplified molecular-input line-entry system SMILES format.

We screened candidate compounds through the Swiss TargetPrediction^15^ (http://www.swisstargetprediction.ch/) database and predicted targets using Probability > 0 as the screening criteria. The screened targets were combined and de-weighted to be the targets of the main active ingredient of AM.

### Crossover genes screening of AM in the treatment of vitiligo and COVID-19

This research does not involve the ethics of human and animal experiments.To identify potential targets of AM for vitiligo and COVID-19, we searched the comprehensive gene expression database GEO^16^, (http://www.ncbi.nlm.nih.gov/geo). The species "human" was selected as the study subject and the dataset GSE75819 with samples from healthy volunteers and vitiligo lesion skin samples. Differentially expressed genes (DEGs) between lesioned and healthy volunteers were screened by GEO2R (http://www.ncbi.nlm.nih.gov/geo/geo2r). Probe sets without corresponding gene symbols or multiple probe sets were removed or averaged, respectively. adj. P values < 0.05 and |LogFC|< 1 were considered statistically significant.

To identify the disease targets of AM for the treatment of COVID-19, we used the Genecards website (https://www.genecards.org) to search for COVID-19 disease related targets using "COVID-19", "coronavirus disease", and "coronary pneumonia" as search terms.

Then the disease targets of vitiligo and COVID-19 were matched with the targets of the active ingredients of AM and imported into Venny 2.1 online website (https://bioinfogp.cnb.csic.es/tools/venny/index.html), which was displayed as a Venn diagram, and the overlapping part intersection of the two was the crossover genes of AM for vitiligo and COVID-19.

To investigate the clustering of the crossover genes in the GSE75819 dataset, we finally drew the clustering heatmap of the crossover genes through the ImageGP website (http://www.ehbio.com/ImageGP/index.php/Home/Index/index.html).

### GO enrichment analysis and KEGG pathway analysis

To investigate GO and KEGG analysis, we imported crossover genes into the Metascape database^17,18^ (https://metascape.org/gp/index.html#/main/step1). Where "Select Identifier" was selected as "OFFICIAL_GENE_ SYMBOL" and "Homo sapiens" was selected for GO annotation and KEGG pathway enrichment analysis of the targets at P < 0.05. The top 10 ranking items were selected and the results were visualized and analyzed through the bioinformatics website (http://www.bioinformatics.com.cn/).

### Protein–Protein Interaction (PPI) network construction and screening of core cross targets of AM in the treatment of vitiligo and COVID-19

To study protein–protein interactions at crossover targets, the STRING database^19^ (https://stringdb.org/) was used to construct a PPI network of crossover genes of AM sapiens for vitiligo and COVID-19. "Homo sapiens" was selected in the parameter Organism, "Medium confidence 0.400)" was selected in the "Minimum required interaction score", and the other parameters were kept at their default values. Then, we filtered out the top 5 ranked core crossover genes by cytohubba plugin in Cytoscape software.

Finally, we constructed the "active ingredient-crossover genes" network by Cytoscape software and screened the top 10 ranked core ingredients. The core crossover geness in the PPI network were matched with those in the "active ingredient-crossover geness" network and imported into the Venny 2.1 online website, which was presented as a Venn diagram. The overlap between the two crossover) is the most important core crossover target for AM for vitiligo and COVID-19.

### Construction of "Drug—Active Ingredient—crossover genes—disease Pathway" network

In order to investigate the relationship between "drug—active ingredient—crossover genes—disease pathway", drugs, active ingredients, predicted crossover genes and the disease pathways were imported into Cytoscape software to construct a "drug-active ingredient-crossover genes-disease pathway" network.

### Ethics statement

This research does not involve the ethics of human and animal experiments.

### Relevant institutional, national, and international guidelines and legislation

The authors confirm that all methods were performed in accordance with the relevant guidelines in the “Materials and methods” section.

## Results

### The main active ingredients of AM

**Figure 2:**
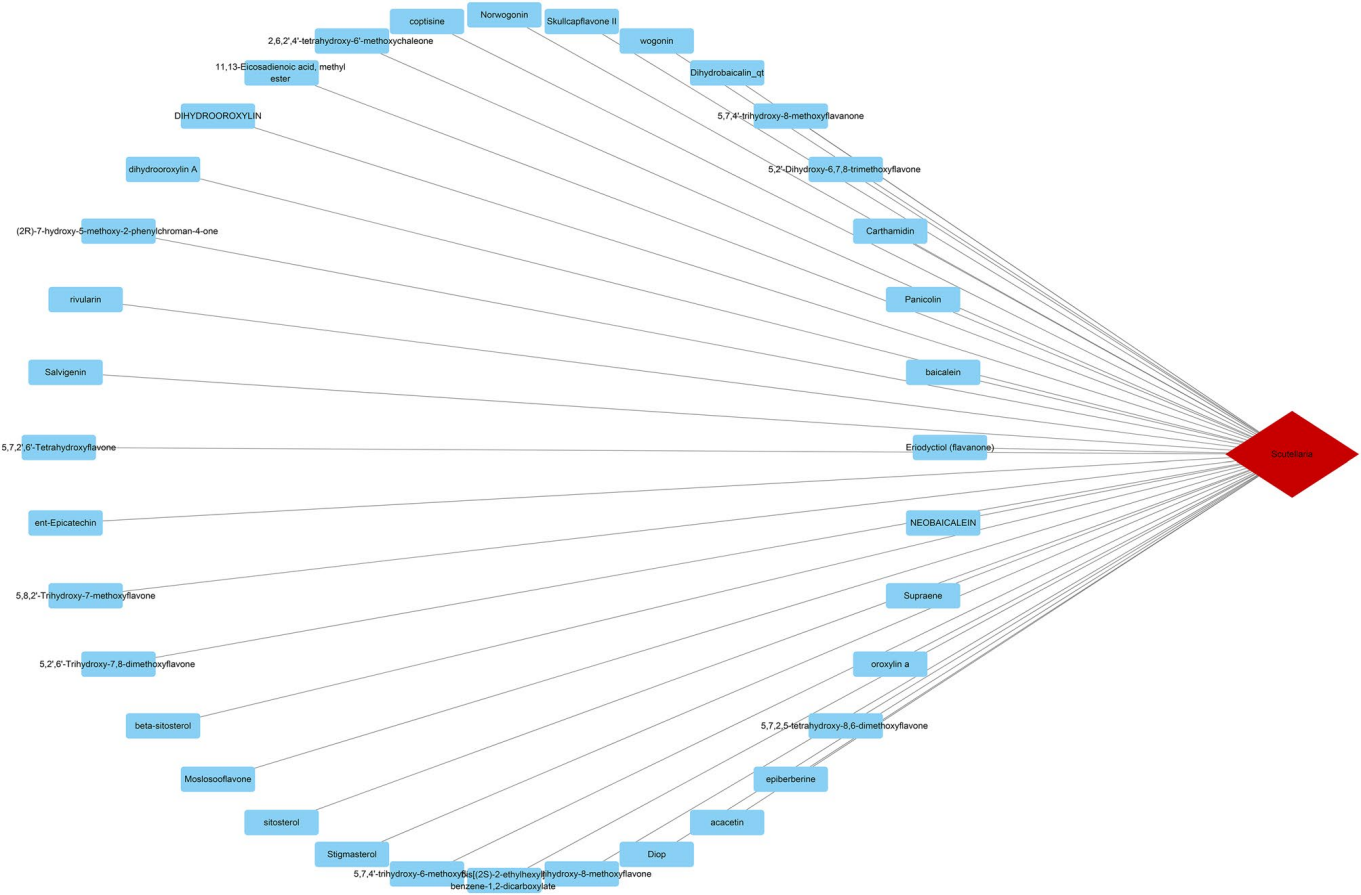
Drug-active ingredient network. Red diamonds represent drugs, blue rectangles represent active ingredients.

To screen the main active components of AM, a total of 143 AM chemical components were retrieved using the TCMSP database. According to the conditions of OB ≥ 30% and DL ≥ 0.18, 36 candidate compounds were screened as the main active components of AM, including flavonoids: baicalein (MOL002714), NEOBAICALEIN (MOL002934), Skullcapflavone II (MOL002927), and wogonin (MOL000173), etc. Then we constructed the "drug—active ingredient" network by Cytoscape software. (Fig. 2, Table 1).

**Table 1:** Basic information of the main active ingredients of Astragalus membranaceus.

Mol ID	Molecule name	OB (%)	DL
MOL002934	NEOBAICALEIN	104.3446052	0.43917
MOL002932	Panicolin	76.25704989	0.2915
MOL012246	5,7,4′-trihydroxy-8-methoxyflavanone	74.23522001	0.26479
MOL002927	Skullcapflavone II	69.51043398	0.4379
MOL002911	2,6,2′,4′-tetrahydroxy-6′-methoxychaleone	69.03987557	0.21994
MOL002937	DIHYDROOROXYLIN	66.06173872	0.23057
MOL000228	(2R)-7-hydroxy-5-methoxy-2-phenylchroman-4-one	55.23317389	0.20163
MOL002915	Salvigenin	49.06592606	0.33279
MOL000073	ent-Epicatechin	48.95984114	0.24162
MOL002917	5,2′,6′-Trihydroxy-7,8-dimethoxyflavone	45.04742802	0.33057
MOL008206	Moslosooflavone	44.08795959	0.25331
MOL000449	Stigmasterol	43.82985158	0.75665
MOL001490	bis[(2S)-2-ethylhexyl] benzene-1,2-dicarboxylate	43.59332547	0.34531
MOL002879	Diop	43.59332547	0.39247
MOL002897	Epiberberine	43.09233228	0.7761
MOL002928	Oroxylin a	41.367569	0.23233
MOL002914	Eriodyctiol (flavanone)	41.35042713	0.2436
MOL002910	Carthamidin	41.15096273	0.24189
MOL002913	Dihydrobaicalin_qt	40.03778103	0.20722
MOL000525	Norwogonin	39.40397184	0.20723
MOL010415	11,13-Eicosadienoic acid, methyl ester	39.27534422	0.2289
MOL002926	Dihydrooroxylin A	38.71506565	0.22987
MOL012266	Rivularin	37.94023355	0.3663
MOL002925	5,7,2′,6′-Tetrahydroxyflavone	37.01348688	0.24382
MOL002908	5,8,2′-Trihydroxy-7-methoxyflavone	37.00837363	0.26546
MOL000358	beta-Sitosterol	36.91390583	0.75123
MOL000359	Sitosterol	36.91390583	0.7512
MOL012245	5,7,4′-trihydroxy-6-methoxyflavanone	36.62688628	0.26833
MOL002933	5,7,4′-Trihydroxy-8-methoxyflavone	36.56200469	0.26666
MOL001689	Acacetin	34.97357273	0.24082
MOL002909	5,7,2,5-tetrahydroxy-8,6-dimethoxyflavone	33.81582599	0.44739
MOL001506	SUPRAENE	33.54594264	0.42161
MOL002714	Baicalein	33.51891869	0.20888
MOL000552	5,2′-Dihydroxy-6,7,8-trimethoxyflavone	31.71246493	0.35462
MOL000173	Wogonin	30.68456706	0.22942
MOL001458	Coptisine	30.671852	0.85647

### Target of active ingredients of AM

In order to screen the active targets of AM active ingredients, the 36 obtained AM active ingredients were imported into the Swiss Target Prediction database platform, and a total of 2336 targets were obtained, of which, 2,6,2′,4′-tetrahydroxy-6′-methoxychaleone,2R)-7-hydroxy-5-methoxy-2-phenylchroman-4-one,ent-Epicatechin, Dihydrobaicalin_qt and 5,7,2,5-tetrahydroxy-8,6-dimethoxyflavone, no effect was found The target point is removed. Finally, integrate all the targets screened by the database platform, delete duplicate or invalid targets, and obtain a total of 448 targets for the active ingredients of AM (Supplementary File S1).

### Crossover genes of AM in the treatment of vitiligo and COVID-19

**Figure 3:**
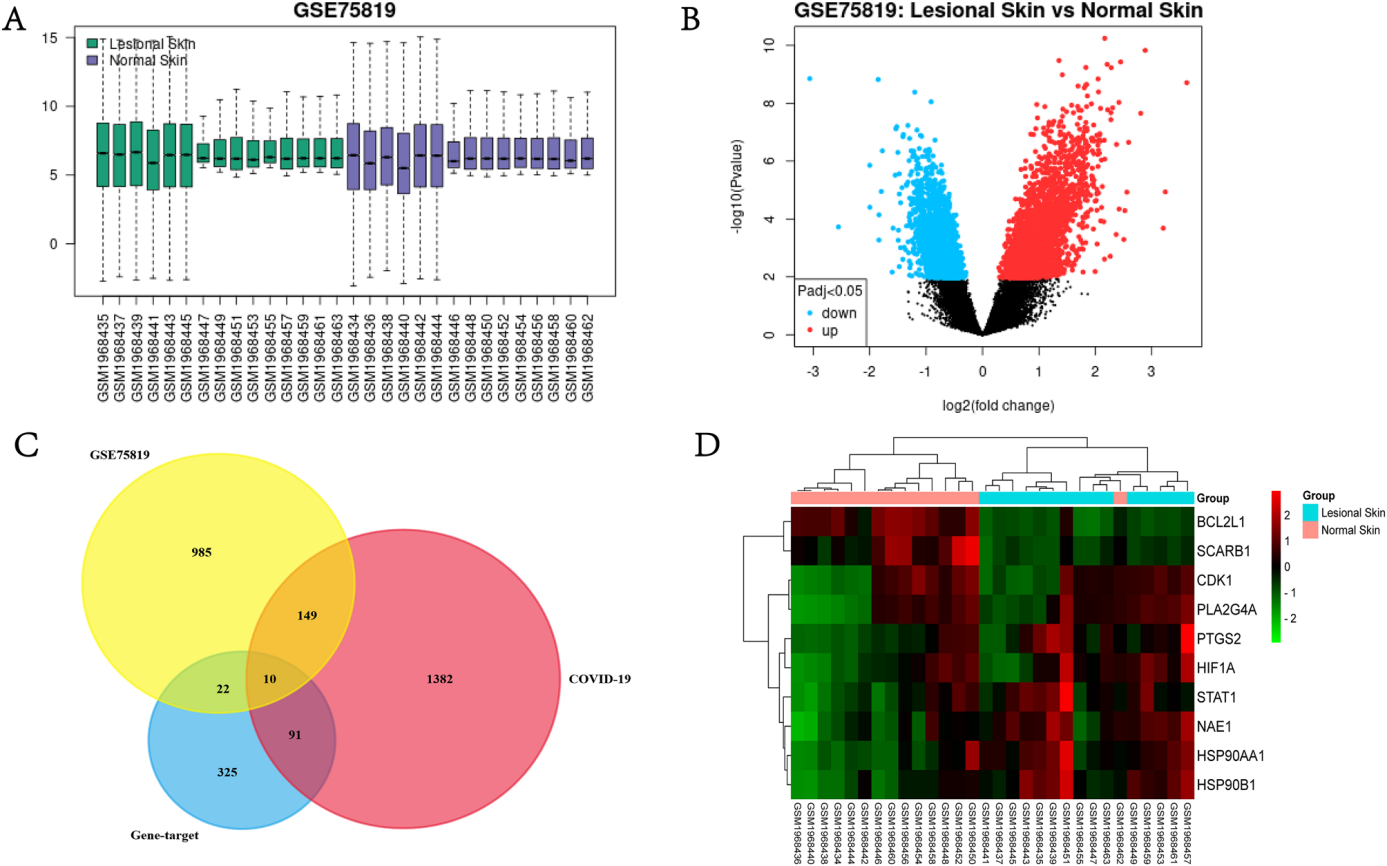
Cross-gene screening of Astragalus in the treatment of vitiligo and COVID-19. (**A**) Quality assessment of the dataset GSE75819. (**B**) Up- and down-regulation relationships of differentially expressed genes in the dataset GSE75819, represented by volcano plots. Up-regulated genes are represented in red, Down-regulated genes are represented in blue. (**C**) Crossover gene screen between GSE75819, drug target and COVID-19. (**D**) Clustering analysis of crossover genes between vitiligo lesioned skin and normal human skin in the GSE75819 dataset. Red represents up-regulated expression and green represents down-regulated expression.

To investigate the common cross-over genes between AM, COVID-19, and vitiligo, we identified 1166 disease targets in vitiligo by analyzing the GSE75819 dataset to identify the relationship between up- and down-regulated genes. 1632 COVID-19 disease-related targets were screened through the gene card website (Fig. 3A and B). Finally, we mapped the 448 targets of the active ingredients of AM to the disease targets of vitiligo and COVID-19, removed the duplicate targets, and obtained 10 gong cross-targets (Fig. 3C and Table 2), which are the cross-targets of AM for vitiligo and COVID-19 disease sites. To investigate the clustering analysis of the cross-targets in vitiligo patients, a clustering heat map of the 10 cross-targets was drawn through an online website for clustering analysis of the gene set GSE75819 (Fig. 3D).

**Table 2:** 10 intersection genes of drug prediction targets, vitiligo and COVID-19.

ID	Gene.symbol	Gene.title	adj.P.Val	P.Value	logFC
ILMN_2054297	PTGS2	Prostaglandin-endoperoxide synthase 2	0.00968477	0.00109	1.5983493
ILMN_1747911	CDK1	Cyclin dependent kinase 1	0.0000569	0.000000368	1.3881929
ILMN_1690105	STAT1	Signal transducer and activator of transcription 1	0.00354559	0.000251	1.1446364
ILMN_1654118	BCL2L1	BCL2 like 1	0.00484829	0.000394	−1.0330706
ILMN_2183409	SCARB1	Scavenger receptor class B member 1	0.00016174	0.0000022	−1.0107721
ILMN_2379788	HIF1A	Hypoxia inducible factor 1 alpha subunit	0.13651097	0.0425	1.2389639
ILMN_1689665	NAE1	NEDD8 activating enzyme E1 subunit 1	0.00254075	0.000153	1.0137769
ILMN_1803561	PLA2G4A	Phospholipase A2 group IVA	0.02313041	0.00381	1.1065647
ILMN_1691097	HSP90AA1	Heat shock protein 90 alpha family class A member 1	0.00045154	0.0000104	1.8500306
ILMN_2096116	HSP90B1	Heat shock protein 90 beta family member 1	0.00122083	4.96E−05	1.4903969

### GO and KEGG pathway analysis of crossover gene

**Figure 4:**
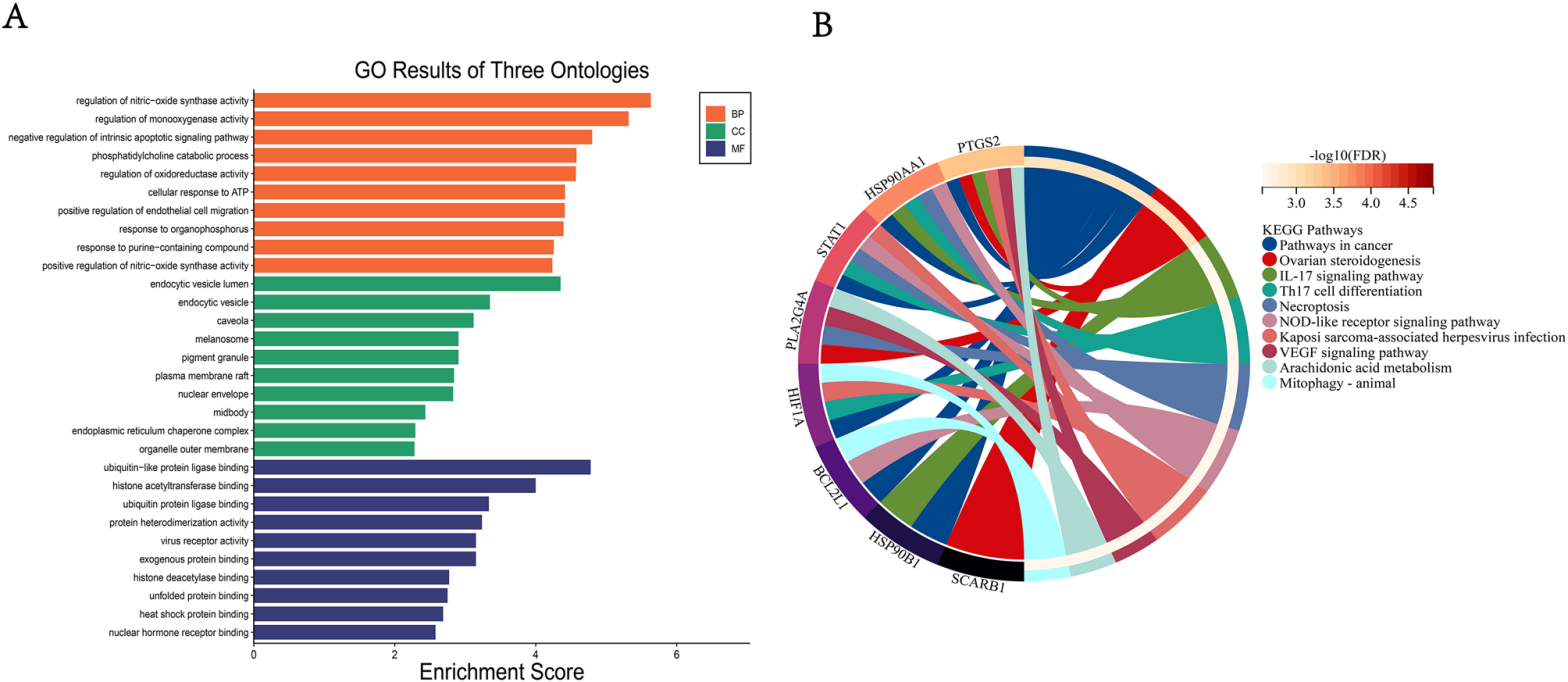
GO and KEGG pathway analysis of cross-targets of Astragalus membranaceus for vitiligo and COVID-19. (**A**) Analysis of the top 10 most significant GO enrichment functions. They are involved in various biological processes (red section), molecular functions (blue section) and cellular components (green section). (**B**) GOChord plot of the top 10 ranked KEGG pathways analysed belonging to crossover genes. Genes are linked by coloured bands to the terms they are assigned to. Genes are ranked according to the observed log fold change (logFC), with log-fold changes shown in decreasing intensity in red squares next to the selected gene.

To investigate the possible mechanisms of action of common crossover genes, we performed GO enrichment analysis and KEGG pathway analysis through the Metascape database. GO enrichment analysis revealed that the main molecular functions (MF) involved in crossover targeting include ubiquitin-like protein ligase binding, histone acetyltransferase binding, and ubiquitin-protein ligase binding; biological processes (BP) mainly include regulation of Nitric oxide synthase activity, regulation of monooxygenase activity, negative regulation of endogenous apoptotic signaling pathway, etc. Cellular components (CC) mainly involve endocytic vesicle lumen, endocytic vesicles, caveolae, and melanosomes (Fig. 4A). KEGG pathway analysis showed that the cross-targets mainly involved IL-17 signaling pathway, Th17 cell differentiation, Necroptosis, NOD-like receptor signaling pathway, Kaposi's sarcoma-associated herpesvirus infection and VEGF signaling pathway (Fig. 4B, Table 3). Taken together, the cross-targets may act through modulation of immune response, apoptosis and viral infection to achieve therapeutic effects in vitiligo and COVID-19.

**Table 3:** KEGG analysis of cross genes in vitiligo patients with COVID-19 treated with AM.

ID	Description	geneID	P value	P.adjust	Count
hsa05200	Pathways in cancer	PTGS2/STAT1/BCL2L1/HIF1A/HSP90AA1/HSP90B1	1.46E−05	0.001259376	6
hsa04913	Ovarian steroidogenesis	PTGS2/SCARB1/PLA2G4A	2.60E−05	0.001259376	3
hsa04657	IL-17 signaling pathway	PTGS2/HSP90AA1/HSP90B1	0.000177606	0.005742583	3
hsa04659	Th17 cell differentiation	STAT1/HIF1A/HSP90AA1	0.000269132	0.00652645	3
hsa04217	Necroptosis	STAT1/PLA2G4A/HSP90AA1	0.00090902	0.017634982	3
hsa04621	NOD-like receptor signaling pathway	STAT1/BCL2L1/HSP90AA1	0.001254258	0.018806283	3
hsa05167	Kaposi sarcoma-associated herpesvirus infection	PTGS2/STAT1/HIF1A	0.001357154	0.018806283	3
hsa04370	VEGF signaling pathway	PTGS2/PLA2G4A	0.002366237	0.027789791	2
hsa00590	Arachidonic acid metabolism	PTGS2/PLA2G4A	0.002693633	0.027789791	2
hsa04137	Mitophagy—animal	BCL2L1/HIF1A	0.002864927	0.027789791	2

### PPI network analysis of crossover gene

**Figure 5:**
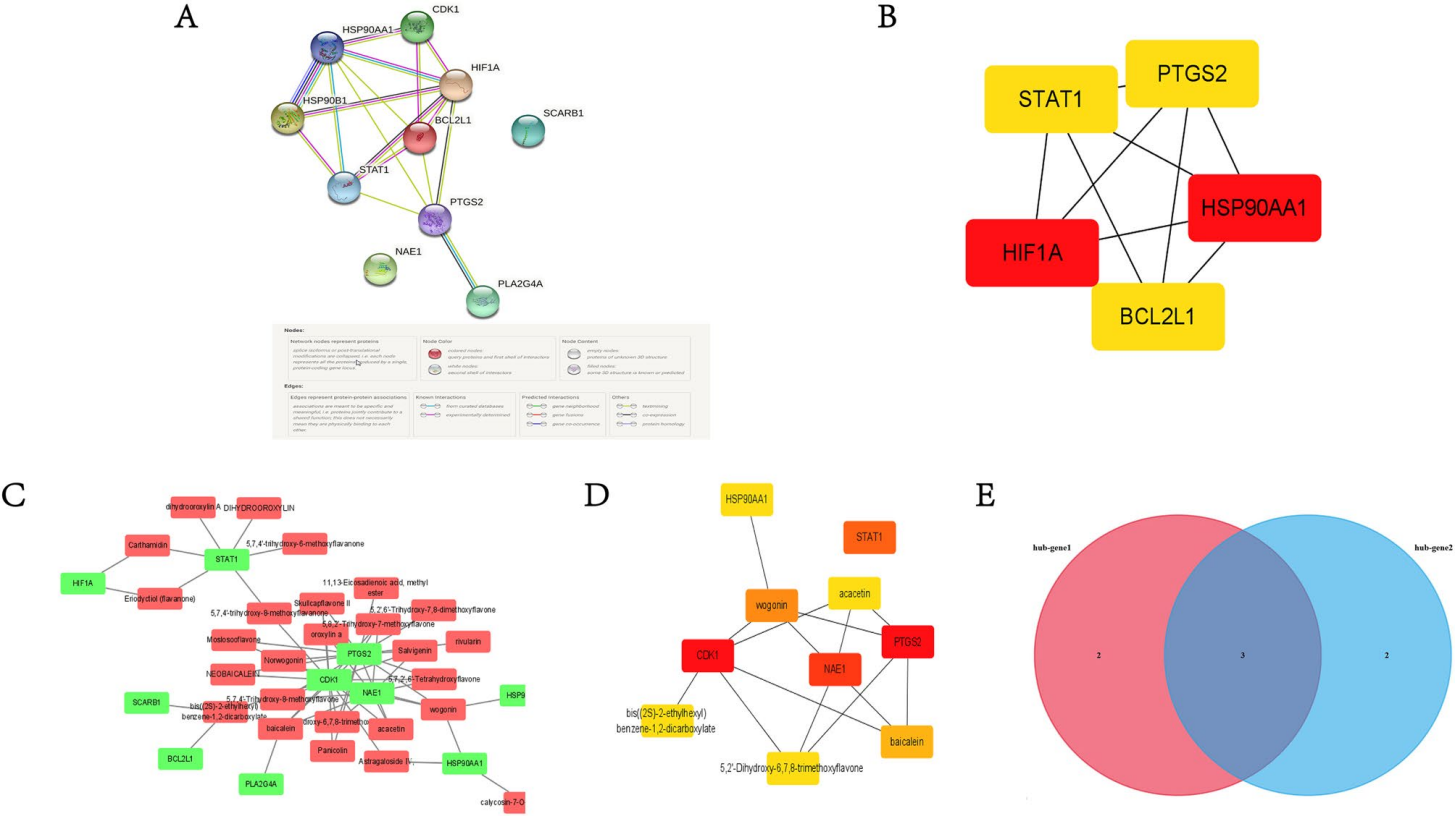
PPI network construction and active ingredient-crossover gene network construction for crossover genes. (**A**) A PPI network of 10 crossed genes was analysed for the gene to gene interactions. The dots represent genes and the connecting lines represent the interactions between genes. (**B**) Top 5 ranked key genes of PPI networks, filtered by cytohubba plugin in Cytoscape software using Degree topology analysis. Darker colours represent greater degrees. (**C**) Network analysis of the active ingredient-crossover gene, red represents the active ingredient, green represents the crossover gene. (**D**) Top 10 ranked active ingredient-crossover gene screen by cytohubba plugin in Cytoscape software using Degree topology analysis. Darker colours represent greater degrees. (**E**) Screening of the most important crossover genes by taking the intersection of (**B**) and (**D**).

To study protein–protein interactions at common crossover gene, we constructed a PPI network using the STRING database (Fig. 5A). This PPI network has 8 nodes and 17 edges. The top 5 ranked core genes, namely PTGS2, STAT1, BCL2L1, HIF1A and HSP90AA1, were then filtered by the Degree topology analysis method in cytohubba plugin (Fig. 5B).

Similarly, we screened the top 10 ranked core ingredients by constructing an "active ingredient-cross-target" network with the cytohubba plug-in (Fig. 5C). These included five major active ingredients acacetin, wogonin, baicalein, bis2S-2-ethylhexyl benzene-1,2-dicarboxylate and 5,2′-Dihydroxy-6,7,8-trimethoxyflavone (Fig. 5D). The core crossover genes obtained by PPI were intersected with those obtained by the "active ingredient-crossover gene" network to obtain the three most important core genes, namely PTGS2, STAT1, and HSP90AA1 (Fig. 5E).

### "Drugs-Active Ingredients-Crossover gene-Disease Pathways" network

**Figure 6:**
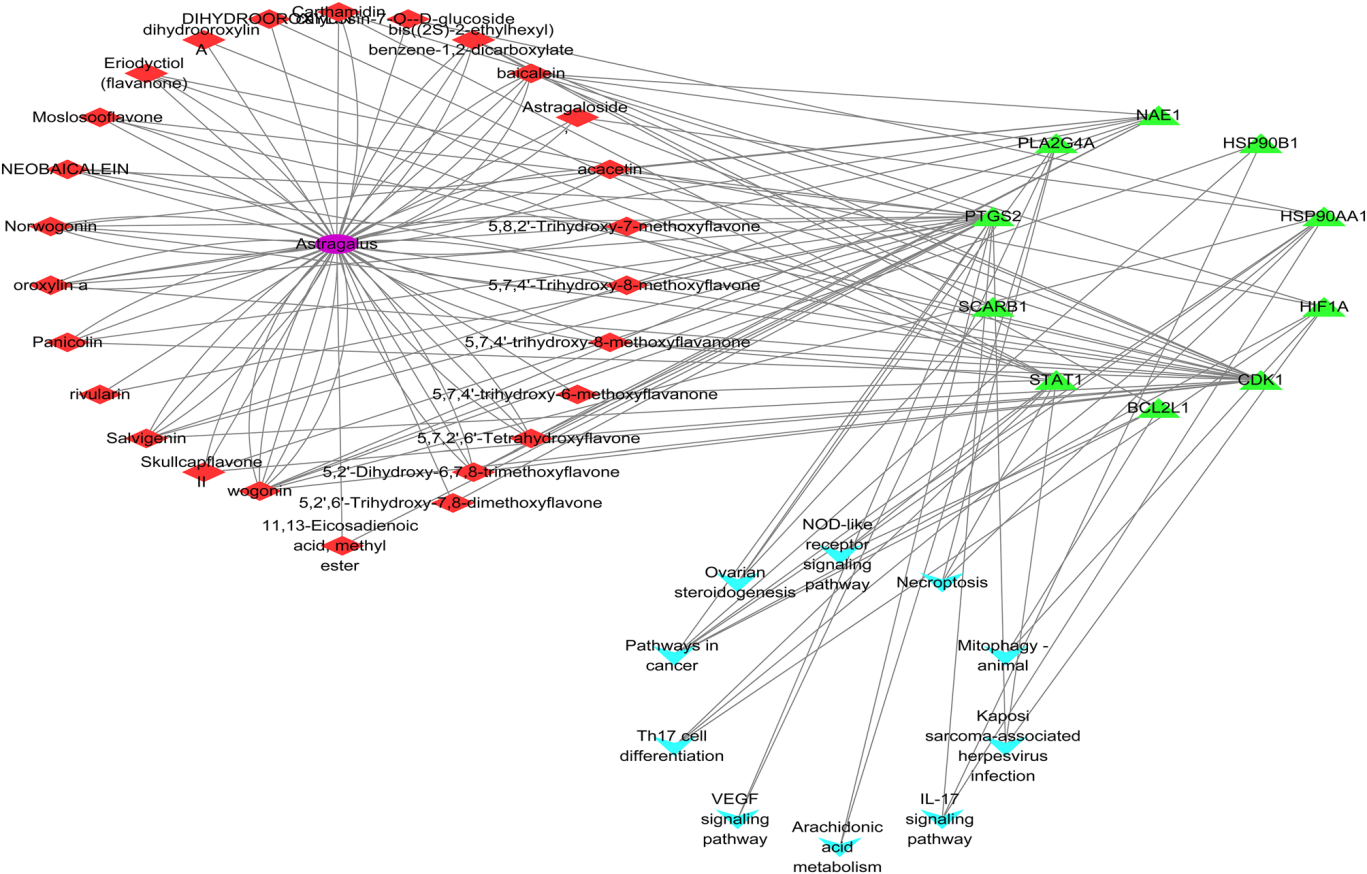
Construction of a drug-active ingredient-target-signalling pathway network. The purple module represents the drug, the red module represents the active ingredient, the green module represents the crossover genre and the blue module represents the KEGG pathway.

To investigate the overall effects of AM for vitiligo and COVID-19, we used cytoscape software to construct a "drug-active-component-crossover gene-disease pathway" network of AM and its 31 active ingredients, 10 common crossover gene and 10 signaling pathways (Fig. 6), in which there are 47 nodes and 148 edges. The interactive network elucidated that the main active ingredients of AM may act on IL-17 signaling pathway, Th17 cell differentiation, Necroptosis, NOD-like receptor signaling pathway, Kaposi's sarcoma-associated herpesvirus infection and VEGF signaling pathway through PTGS2, STAT1, HSP90AA1 and other targets, which are involved in the therapeutic effects of AM on vitiligo and COVID-19.

## Discussion

COVID-19 has spread quickly all over the planet throughout recent years. The COVID-19 pandemic is as yet wild in many nations because of the absence of explicit medications. Vitiligo is considered an autoimmune disease. In immune system sicknesses, oxidative pressure might be brought about by some outer "triggers" like viral diseases^20^. While various questions remain in regards to the long haul sequelae of COVID-19 infection, arising proof recommends the presence of basic immune system complications. Recent literature describes the development of various autoimmune diseases following COVID-19, including vitiligo^21^. Similar disturbances in CD8+ T cells have been described in leukoplakia pathogenesis, particularly in association with HIV infection. Thus, this may represent a common mechanism between post-viral autoimmunity and leukoplakia development. Oxidative stress is a potential catalyst for another autoimmune disease following COVID-19. Infection with SARS-CoV-2 stimulates an overactive immune response, which in turn leads to an overproduction of reactive oxygen species. Severely dysregulated immune responses caused by COVID-19 infection can lead to organ damage through "cytokine storm" or overproduction of inflammatory cytokines and activation of immune cells. Clearly, excessive oxidative stress due to this process leads to the development of COVID-19 sequelae such as acute respiratory distress syndrome. Given that oxidative stress has also been described as a pathogenesis of leukoplakia, this suggests another potential relationship between leukoplakia and COVID-19^22^. It is hence especially significant to investigate a medication that balances the insusceptible status of patients to treat COVID-19 patients with vitiligo. China, a country of more than 1.3 billion people, has managed to contain the outbreak. Traditional Chinese medicine TCM) has made indispensable contributions to the prevention and treatment of COVID-19 infection. Studies have demonstrated the efficacy of AM in patients with both vitiligo and COVID-19. However, little is currently known about the common mechanisms of action and their potential targets in patients with COVID-19-like vitiligo. Here, using network pharmacology and bioinformatics methods, we systematically analyze the main active ingredients, main targets, and signaling pathways of AM on COVID-19 patients with vitiligo.

AM has been widely used to treat vitiligo and COVID-19, but its mechanism of action is unknown. Previous studies have shown that both vitiligo and COVID-19 disease involve dysregulation of the immune-inflammatory response. Five important active components of AM, acacetin, wogonin, baicalein, bis 2S)-2-ethylhexyl) benzene-1, 2-dicarboxylate and 5,2′-Dihydroxy-6,7,8-trimethoxyflavone, were screened and classified as flavonoids. Among them, baicalein is a flavonoid extracted from the root of Scutellaria baicalensis and has been widely used in Asian traditional medicine^23^. It has anti-cytotoxic, anti-inflammatory and anti-tumor effects^24–26^. In an in vitro oxidative stress model of PIG1 induced by h2o2, baicalein protected PIG1 cells from h2o2-induced oxidative stress and apoptosis, and baicalein protected melanocytes most strongly at a concentration of 40 μM^27^. Therefore, baicalein preparation is a feasible treatment for vitiligo. In addition, other studies have reported that baicalin reduces infiltration of CD8+ T cells and expression of CXCL10 and CXCR3 in mouse skin. Baicalin significantly decreased serum cytokines IL-6, TNF-α, IFN-γ, and IL-13)^28^. Furthermore, flavonoids are polyhydroxylated 2-phenylchromones.Their glycosides and some biological isoforms have antiviral activity against SARS coronavirus, MERS-CoV, other human coronavirus, and influenza A virus^29^. Scutellaria baicalensis is rich in flavonoid extracts such as flavonoids, baicalein, baicalin, and their glycosides, which decrease the production of inflammatory cytokines TNF-α, IL-6, monocyte chemoattractant protein-1 MCP-1), and nitric oxide in the lung tissue of mice exposed to influenza A virus, while increasing the synthesis of protective cytokines, IFN-γ, and IL-10^30^. In summary, the above studies have demonstrated the potential therapeutic efficacy of the main active ingredient of Scutellaria baicalensis against vitiligo and COVID-19.

In this study, we obtained three most significant core crossover genes PTGS2, STAT1, and HSP90AA1. Pu et al.^31^ found that PTGS2 and HSP90AA1 are key genes involved in melanopoiesis in vitiligo. Li et al.^32^ found that PTGS2 and HSP90AA1 were associated with oxidative stress response. It has been reported that the expression of PTGS2 is upregulated when cells are stimulated by hydrogen peroxide^33,34^. HSP90AA1 plays a critical role in signal transduction, protein folding, protein degradation, and morphological evolution^35^; STAT1 is a 91 kDa member of the STAT family that responds to cytokines and growth factors. It is phosphorylated by receptor-associated kinases and translocates to the nucleus where it is involved in apoptosis as a transcriptional activator. It can promote or resist apoptosis depending on the cellular environment and different pathways of interaction^36,37^. Previous studies have shown that STAT1, a transcriptional activator in vitiligo melanocytes, migrates into the nucleus to activate genes involved in cell proliferation and viability that can be activated by the IFN pathway^38,39^. In addition, Yan et al.^40^ found that 160 active ingredients in Lianhua Qingwen capsule could have therapeutic effects on COVID-19 by involving HSP90AA1 signaling. Hu et al.^41^ identified PTGS2 as a critical target in COVID-19-associated ARDS by network pharmacology analysis. Camacho et al.^42^ findings elucidated molecules associated with COVID-19 and revealed six upstream regulators, TNF, IFNG, STAT1, IL1β, IL6, and STAT3. Zhou et al.^43^ showed that S protein enhanced the binding of upstream JAK1 to downstream STAT1 and STAT2, thereby promoting the phosphorylation of STAT1 and STAT2 and the expression of ACE2.Thus, the active components of AM may play a role in regulating immune response, anti-oxidative stress, and anti-apoptosis by down-regulating the expression of PTGS2, STAT1, and HSP90AA1 genes.

Our results showed that co-crossover genes were significantly enriched in IL-17 signaling pathway, Th17 cell differentiation signaling, nod-like receptor signaling pathway, and these signaling pathways, which have been shown to be involved in mediating immune regulation in vitiligo^44–46^. These results are consistent with a recent study that found elevated cytokine levels in the plasma of COVID-19 patients^47^. Several independent studies have confirmed the elevation of IL-17 in the serum of vitiligo patients, which tends to correlate with the extent and course of the disease. More importantly, IL-17 levels decreased after successful nb-uvb treatment^48^. IL-17+ cells can be found almost in perilesional and perilesional skin of vitiligo patients, suggesting that they play a driving role in pathogenesis^39^. In addition, it is noteworthy that Hasan et al.^49^ reported that SARS-CoV-2 infection strongly activated the IL-17 signaling pathway compared with other respiratory viruses. They also observed that all SARS-CoV-2 datasets showed particularly strong inflammatory responses triggered by IL-17 activation, indicating the importance of IL-17 in SARS-CoV-2 infection. At the same time, IL-17 is also increased in critically ill patients compared with non-critically ill patients. In addition, Li et al.^50^ found that Qingfei Paidu Decoction could prevent the transition from mild to severe stage in COVID-19 patients through IL-17 signaling pathway, Th17 cell differentiation and other signaling pathways. A study published in April 2021 showed that SARS-CoV-2 open reading frame 8 ORF8) binds to IL17 receptor and activates IL17 signaling pathway. Blocking IL17RA with an antibody reduced IL17-mediated inflammation in lung and liver infected with SARS-CoV2ORF8 pseudovirus^51^. This report supports that SARS-CoV-2 significantly initiates IL17-mediated inflammatory responses and may exacerbate the severity of the disease. Therefore, blocking IL-17 may be a feasible strategy to reduce multiple organ damage and disease severity. Thus, the main active components of AM may exert their efficacy in the treatment of COVID-19 disease by blocking signaling pathways such as IL-17 signaling pathway, Th17 cell differentiation signaling, and nod-like receptor signaling pathway.

However, there are some limitations to our research as well. We have not been able to conduct experiments to demonstrate the therapeutic effect of AM on COVID-19 and vitiligo due to its complexity. The SARS-CoV-2 infection experimental model and the vitiligo model confirm AM's direct curative effect. However, numerous studies have demonstrated the combined effects of anti-inflammatory and antiviral effects, so we are convinced that AM may be beneficial for COVID-19 and vitiligo. Our findings indicate that AM requires related and more in-depth research right away.

## Summary

In conclusion, we explored potential compounds in AM combined with anti-vitiligo and anti-COVID-19 activities using network pharmacology, bioinformatics approaches. Our results suggest that flavonoids screened from AM could have potential in the treatment of vitiligo and COVID-19. This study also suggests that bioinformatics and network pharmacology approaches can serve as effective strategies for discovering potential compounds from herbal medicines against COVID-19 combined with other diseases.

## Supplementary Information

Supplementary Information.

## Data Availability

The datasets used and/or analysed during the current study available from the corresponding author on reasonable request.
