# Author Correction: Based on network pharmacology and bioinformatics to analyze the mechanism of action of Astragalus membranaceus in the treatment of vitiligo and COVID-19

**DOI:** 10.1038/s41598-023-34471-7

**Published:** 2023-05-08

**Authors:** Yaojun Wang, Ming Ding, Jiaoni Chi, Tao Wang, Yue Zhang, Zhimin Li, Qiang Li

**Affiliations:** 1 Graduate School, Hebei North University, Zhangjiakou, 075000 China; 2 Air Force Clinical College, The Fifth School of Clinical Medicine, Anhui Medical University, Hefei, 230032 China; 3 Department of Dermatology, Air Force Medical Center, PLA, Beijing, 100142 China

Correction to: *Scientific Reports* 10.1038/s41598-023-29207-6, published online 08 March 2023

The original version of this Article contained an error in Affiliation 2, which was incorrectly given as ‘The Fifth School of Clinical Medicine, Anhui Medical University, Hefei, 230032 China’. The correct affiliation is listed below:

Air Force Clinical College, The Fifth School of Clinical Medicine, Anhui Medical University, Hefei, 230032 China.

