# Supplementary material for: Based on network pharmacology and bioinformatics to analyze the mechanism of action of Astragalus membranaceus in the treatment of vitiligo and COVID-19
Source: Sci Rep. 2023 Mar 8;13:3884. doi: 10.1038/s41598-023-29207-6 (PMC9993359; doi:10.1038/s41598-023-29207-6)
Supplement: Supplementary file 1 — Supplementary Information. [file 41598_2023_29207_MOESM1_ESM.doc]

**Supplementary File S1. Predictive Targets Corresponding to Main Active Components of Astragalus membranaceus**

| SwissTargetPrediction | Target | possibility |
| --- | --- | --- |
| 5,2',6'-Trihydroxy-7,8-dimethoxyflavone | RPS6KA3 | 0.103186937 |
| 5,2',6'-Trihydroxy-7,8-dimethoxyflavone | CA2 | 0.103186937 |
| 5,2',6'-Trihydroxy-7,8-dimethoxyflavone | CA12 | 0.103186937 |
| 5,2',6'-Trihydroxy-7,8-dimethoxyflavone | AKR1B1 | 0.103186937 |
| 5,2',6'-Trihydroxy-7,8-dimethoxyflavone | ACHE | 0.103186937 |
| 5,2',6'-Trihydroxy-7,8-dimethoxyflavone | NQO2 | 0.103186937 |
| 5,2',6'-Trihydroxy-7,8-dimethoxyflavone | ADRA2C | 0.103186937 |
| 5,2',6'-Trihydroxy-7,8-dimethoxyflavone | NMUR2 | 0.103186937 |
| 5,2',6'-Trihydroxy-7,8-dimethoxyflavone | ADRA2A | 0.103186937 |
| 5,2',6'-Trihydroxy-7,8-dimethoxyflavone | XDH | 0.103186937 |
| 5,2',6'-Trihydroxy-7,8-dimethoxyflavone | TNF | 0.103186937 |
| 5,2',6'-Trihydroxy-7,8-dimethoxyflavone | IL2 | 0.103186937 |
| 5,2',6'-Trihydroxy-7,8-dimethoxyflavone | PTGS2 | 0.103186937 |
| 5,2',6'-Trihydroxy-7,8-dimethoxyflavone | ADORA1 | 0.103186937 |
| 5,2',6'-Trihydroxy-7,8-dimethoxyflavone | CD38 | 0.103186937 |
| 5,2',6'-Trihydroxy-7,8-dimethoxyflavone | IMPDH2 | 0.103186937 |
| 5,2',6'-Trihydroxy-7,8-dimethoxyflavone | HPRT1 | 0.103186937 |
| 5,2',6'-Trihydroxy-7,8-dimethoxyflavone | MAG | 0.103186937 |
| 5,2',6'-Trihydroxy-7,8-dimethoxyflavone | FDFT1 | 0.103186937 |
| 5,2',6'-Trihydroxy-7,8-dimethoxyflavone | CA4 | 0.103186937 |
| 5,7,4'-trihydroxy-8-methoxyflavanone | CYP19A1 | 0.198389229 |
| 5,7,4'-trihydroxy-8-methoxyflavanone | TAS2R31 | 0.166798772 |
| 5,7,4'-trihydroxy-8-methoxyflavanone | HSD17B1 | 0.158886034 |
| 5,7,4'-trihydroxy-8-methoxyflavanone | PTGS1 | 0.143102156 |
| 5,7,4'-trihydroxy-8-methoxyflavanone | CYP1B1 | 0.135202128 |
| 5,7,4'-trihydroxy-8-methoxyflavanone | ESR2 | 0.135202128 |
| 5,7,4'-trihydroxy-8-methoxyflavanone | CA7 | 0.12730257 |
| 5,7,4'-trihydroxy-8-methoxyflavanone | ESR1 | 0.12730257 |
| 5,7,4'-trihydroxy-8-methoxyflavanone | CA12 | 0.12730257 |
| 5,7,4'-trihydroxy-8-methoxyflavanone | CA4 | 0.12730257 |
| 5,7,4'-trihydroxy-8-methoxyflavanone | ABCG2 | 0.119403562 |
| 5,7,4'-trihydroxy-8-methoxyflavanone | MAOB | 0.119403562 |
| 5,7,4'-trihydroxy-8-methoxyflavanone | ABCC1 | 0.111501865 |
| 5,7,4'-trihydroxy-8-methoxyflavanone | ADORA1 | 0.111501865 |
| 5,7,4'-trihydroxy-8-methoxyflavanone | ADORA3 | 0.111501865 |
| 5,7,4'-trihydroxy-8-methoxyflavanone | SHBG | 0.111501865 |
| 5,7,4'-trihydroxy-8-methoxyflavanone | CBR1 | 0.111501865 |
| 5,7,4'-trihydroxy-8-methoxyflavanone | CA3 | 0.111501865 |
| 5,7,4'-trihydroxy-8-methoxyflavanone | ABCB1 | 0.111501865 |
| 5,7,4'-trihydroxy-8-methoxyflavanone | MMP13 | 0.111501865 |
| 5,7,4'-trihydroxy-8-methoxyflavanone | MMP12 | 0.111501865 |
| 5,7,4'-trihydroxy-8-methoxyflavanone | SRC | 0.111501865 |
| 5,7,4'-trihydroxy-8-methoxyflavanone | PLA2G1B | 0.111501865 |
| 5,7,4'-trihydroxy-8-methoxyflavanone | AKR1C3 | 0.111501865 |
| 5,7,4'-trihydroxy-8-methoxyflavanone | CA2 | 0.111501865 |
| 5,7,4'-trihydroxy-8-methoxyflavanone | CA1 | 0.111501865 |
| 5,7,4'-trihydroxy-8-methoxyflavanone | GRM5 | 0.111501865 |
| 5,7,4'-trihydroxy-8-methoxyflavanone | CA6 | 0.111501865 |
| 5,7,4'-trihydroxy-8-methoxyflavanone | CA13 | 0.111501865 |
| 5,7,4'-trihydroxy-8-methoxyflavanone | CA5B | 0.111501865 |
| 5,7,4'-trihydroxy-8-methoxyflavanone | CA5A | 0.111501865 |
| 5,7,4'-trihydroxy-8-methoxyflavanone | CES1 | 0.111501865 |
| 5,7,4'-trihydroxy-8-methoxyflavanone | CES2 | 0.111501865 |
| 5,7,4'-trihydroxy-8-methoxyflavanone | KLK1 | 0.111501865 |
| 5,7,4'-trihydroxy-8-methoxyflavanone | KLK2 | 0.111501865 |
| 5,7,4'-trihydroxy-8-methoxyflavanone | HSD17B2 | 0.111501865 |
| 5,7,4'-trihydroxy-8-methoxyflavanone | SLC5A2 | 0.111501865 |
| 5,7,4'-trihydroxy-8-methoxyflavanone | BACE1 | 0.111501865 |
| 5,7,4'-trihydroxy-8-methoxyflavanone | POLB | 0.111501865 |
| 5,7,4'-trihydroxy-8-methoxyflavanone | MET | 0.111501865 |
| 5,7,4'-trihydroxy-8-methoxyflavanone | PLA2G2A | 0.111501865 |
| 5,7,4'-trihydroxy-8-methoxyflavanone | PLA2G5 | 0.111501865 |
| 5,7,4'-trihydroxy-8-methoxyflavanone | PLA2G10 | 0.111501865 |
| 5,7,4'-trihydroxy-8-methoxyflavanone | MMP2 | 0.111501865 |
| 5,7,4'-trihydroxy-8-methoxyflavanone | CDK2 | 0.111501865 |
| 5,7,4'-trihydroxy-8-methoxyflavanone | CDK4 | 0.111501865 |
| 5,7,4'-trihydroxy-8-methoxyflavanone | CHEK1 | 0.111501865 |
| 5,7,4'-trihydroxy-8-methoxyflavanone | WEE1 | 0.111501865 |
| 5,7,4'-trihydroxy-8-methoxyflavanone | YWHAG | 0.111501865 |
| 5,7,4'-trihydroxy-8-methoxyflavanone | KDR | 0.111501865 |
| 5,7,4'-trihydroxy-8-methoxyflavanone | FGFR1 | 0.111501865 |
| 5,7,4'-trihydroxy-8-methoxyflavanone | CDK1 | 0.111501865 |
| 5,7,4'-trihydroxy-8-methoxyflavanone | SERPINE1 | 0.111501865 |
| 5,7,4'-trihydroxy-8-methoxyflavanone | DYRK1A | 0.111501865 |
| 5,7,4'-trihydroxy-8-methoxyflavanone | NOX4 | 0.111501865 |
| 5,7,4'-trihydroxy-8-methoxyflavanone | TYR | 0.111501865 |
| 5,7,4'-trihydroxy-8-methoxyflavanone | CHRNA7 | 0.111501865 |
| 5,7,4'-trihydroxy-8-methoxyflavanone | MAPT | 0.111501865 |
| 5,7,4'-trihydroxy-8-methoxyflavanone | DNMT1 | 0.111501865 |
| 5,7,4'-trihydroxy-8-methoxyflavanone | BCL2 | 0.111501865 |
| 5,7,4'-trihydroxy-8-methoxyflavanone | STAT1 | 0.111501865 |
| 5,7,4'-trihydroxy-8-methoxyflavanone | SQLE | 0.111501865 |
| 5,7,4'-trihydroxy-8-methoxyflavanone | APP | 0.111501865 |
| 5,7,4'-trihydroxy-8-methoxyflavanone | ESRRA | 0.111501865 |
| 5,7,4'-trihydroxy-8-methoxyflavanone | ESRRB | 0.111501865 |
| 5,7,4'-trihydroxy-8-methoxyflavanone | CLK1 | 0.111501865 |
| 5,7,4'-trihydroxy-8-methoxyflavanone | DYRK1B | 0.111501865 |
| 5,7,4'-trihydroxy-8-methoxyflavanone | STS | 0.111501865 |
| 5,7,4'-trihydroxy-8-methoxyflavanone | ACHE | 0.111501865 |
| 5,7,4'-trihydroxy-8-methoxyflavanone | RXRA | 0.111501865 |
| 5,7,4'-trihydroxy-8-methoxyflavanone | EDNRA | 0.111501865 |
| bis((2S)-2-ethylhexyl) benzene-1,2-dicarboxylate | PRKCD | 0.115736675 |
| bis((2S)-2-ethylhexyl) benzene-1,2-dicarboxylate | PTPN1 | 0.115736675 |
| bis((2S)-2-ethylhexyl) benzene-1,2-dicarboxylate | PRKCA | 0.115736675 |
| bis((2S)-2-ethylhexyl) benzene-1,2-dicarboxylate | PTPN2 | 0.115736675 |
| bis((2S)-2-ethylhexyl) benzene-1,2-dicarboxylate | AR | 0.115736675 |
| bis((2S)-2-ethylhexyl) benzene-1,2-dicarboxylate | CTSK | 0.115736675 |
| bis((2S)-2-ethylhexyl) benzene-1,2-dicarboxylate | CTSS | 0.115736675 |
| bis((2S)-2-ethylhexyl) benzene-1,2-dicarboxylate | CTSL | 0.115736675 |
| bis((2S)-2-ethylhexyl) benzene-1,2-dicarboxylate | CTSB | 0.115736675 |
| bis((2S)-2-ethylhexyl) benzene-1,2-dicarboxylate | FKBP1A | 0.115736675 |
| bis((2S)-2-ethylhexyl) benzene-1,2-dicarboxylate | PDE10A | 0.115736675 |
| bis((2S)-2-ethylhexyl) benzene-1,2-dicarboxylate | GRM2 | 0.115736675 |
| bis((2S)-2-ethylhexyl) benzene-1,2-dicarboxylate | CCND1 CDK4 | 0.115736675 |
| bis((2S)-2-ethylhexyl) benzene-1,2-dicarboxylate | CDK1 CCNB1 | 0.115736675 |
| bis((2S)-2-ethylhexyl) benzene-1,2-dicarboxylate | CCNE1 CDK2 | 0.115736675 |
| bis((2S)-2-ethylhexyl) benzene-1,2-dicarboxylate | MAPK14 | 0.115736675 |
| bis((2S)-2-ethylhexyl) benzene-1,2-dicarboxylate | TSPO | 0.115736675 |
| bis((2S)-2-ethylhexyl) benzene-1,2-dicarboxylate | PTGER1 | 0.115736675 |
| bis((2S)-2-ethylhexyl) benzene-1,2-dicarboxylate | GABRB3 GABRA3 GABRG2 | 0.115736675 |
| bis((2S)-2-ethylhexyl) benzene-1,2-dicarboxylate | GABRB3 GABRG2 GABRA1 | 0.115736675 |
| bis((2S)-2-ethylhexyl) benzene-1,2-dicarboxylate | GABRB3 GABRG2 GABRA5 | 0.115736675 |
| bis((2S)-2-ethylhexyl) benzene-1,2-dicarboxylate | GABRA2 GABRB3 GABRG2 | 0.115736675 |
| bis((2S)-2-ethylhexyl) benzene-1,2-dicarboxylate | PDE5A | 0.115736675 |
| bis((2S)-2-ethylhexyl) benzene-1,2-dicarboxylate | BCL2 | 0.115736675 |
| bis((2S)-2-ethylhexyl) benzene-1,2-dicarboxylate | GPBAR1 | 0.115736675 |
| bis((2S)-2-ethylhexyl) benzene-1,2-dicarboxylate | FNTA FNTB | 0.115736675 |
| bis((2S)-2-ethylhexyl) benzene-1,2-dicarboxylate | PREP | 0.115736675 |
| bis((2S)-2-ethylhexyl) benzene-1,2-dicarboxylate | MAPK11 | 0.115736675 |
| bis((2S)-2-ethylhexyl) benzene-1,2-dicarboxylate | ACE | 0.115736675 |
| bis((2S)-2-ethylhexyl) benzene-1,2-dicarboxylate | P2RX7 | 0.115736675 |
| bis((2S)-2-ethylhexyl) benzene-1,2-dicarboxylate | GABRG2 GABRB3 GABRA6 | 0.115736675 |
| bis((2S)-2-ethylhexyl) benzene-1,2-dicarboxylate | ELOVL6 | 0.115736675 |
| bis((2S)-2-ethylhexyl) benzene-1,2-dicarboxylate | KCNA5 | 0.115736675 |
| bis((2S)-2-ethylhexyl) benzene-1,2-dicarboxylate | PGGT1B FNTA | 0.115736675 |
| bis((2S)-2-ethylhexyl) benzene-1,2-dicarboxylate | TNFRSF1A | 0.115736675 |
| bis((2S)-2-ethylhexyl) benzene-1,2-dicarboxylate | SCN10A | 0.115736675 |
| bis((2S)-2-ethylhexyl) benzene-1,2-dicarboxylate | CPB1 | 0.115736675 |
| bis((2S)-2-ethylhexyl) benzene-1,2-dicarboxylate | MEN1 | 0.115736675 |
| bis((2S)-2-ethylhexyl) benzene-1,2-dicarboxylate | TRPC6 | 0.115736675 |
| bis((2S)-2-ethylhexyl) benzene-1,2-dicarboxylate | TRPC3 | 0.115736675 |
| bis((2S)-2-ethylhexyl) benzene-1,2-dicarboxylate | ELANE | 0.115736675 |
| bis((2S)-2-ethylhexyl) benzene-1,2-dicarboxylate | TRPV4 | 0.115736675 |
| bis((2S)-2-ethylhexyl) benzene-1,2-dicarboxylate | HCRTR2 | 0.115736675 |
| bis((2S)-2-ethylhexyl) benzene-1,2-dicarboxylate | TRPV1 | 0.115736675 |
| bis((2S)-2-ethylhexyl) benzene-1,2-dicarboxylate | MAP3K8 | 0.115736675 |
| bis((2S)-2-ethylhexyl) benzene-1,2-dicarboxylate | HCRTR1 | 0.115736675 |
| bis((2S)-2-ethylhexyl) benzene-1,2-dicarboxylate | TRPA1 | 0.115736675 |
| bis((2S)-2-ethylhexyl) benzene-1,2-dicarboxylate | SLC2A1 | 0.115736675 |
| bis((2S)-2-ethylhexyl) benzene-1,2-dicarboxylate | GRM5 | 0.115736675 |
| bis((2S)-2-ethylhexyl) benzene-1,2-dicarboxylate | ADAM17 | 0.115736675 |
| bis((2S)-2-ethylhexyl) benzene-1,2-dicarboxylate | CMA1 | 0.115736675 |
| bis((2S)-2-ethylhexyl) benzene-1,2-dicarboxylate | PTGDR2 | 0.115736675 |
| bis((2S)-2-ethylhexyl) benzene-1,2-dicarboxylate | SLC2A3 | 0.115736675 |
| bis((2S)-2-ethylhexyl) benzene-1,2-dicarboxylate | SLC2A2 | 0.115736675 |
| bis((2S)-2-ethylhexyl) benzene-1,2-dicarboxylate | ADORA1 | 0.115736675 |
| bis((2S)-2-ethylhexyl) benzene-1,2-dicarboxylate | ADORA2A | 0.115736675 |
| bis((2S)-2-ethylhexyl) benzene-1,2-dicarboxylate | ADORA2B | 0.115736675 |
| bis((2S)-2-ethylhexyl) benzene-1,2-dicarboxylate | MMP9 | 0.115736675 |
| bis((2S)-2-ethylhexyl) benzene-1,2-dicarboxylate | MMP2 | 0.115736675 |
| bis((2S)-2-ethylhexyl) benzene-1,2-dicarboxylate | MMP7 | 0.115736675 |
| bis((2S)-2-ethylhexyl) benzene-1,2-dicarboxylate | RORC | 0.115736675 |
| bis((2S)-2-ethylhexyl) benzene-1,2-dicarboxylate | PABPC1 | 0.115736675 |
| bis((2S)-2-ethylhexyl) benzene-1,2-dicarboxylate | ABL1 | 0.115736675 |
| bis((2S)-2-ethylhexyl) benzene-1,2-dicarboxylate | CCND3 CCND1 CDK4 CCND2 | 0.115736675 |
| bis((2S)-2-ethylhexyl) benzene-1,2-dicarboxylate | CAPN1 | 0.115736675 |
| bis((2S)-2-ethylhexyl) benzene-1,2-dicarboxylate | SCN2A | 0.115736675 |
| bis((2S)-2-ethylhexyl) benzene-1,2-dicarboxylate | PTGES | 0.115736675 |
| bis((2S)-2-ethylhexyl) benzene-1,2-dicarboxylate | PTGFR | 0.115736675 |
| bis((2S)-2-ethylhexyl) benzene-1,2-dicarboxylate | AGTR1 | 0.115736675 |
| bis((2S)-2-ethylhexyl) benzene-1,2-dicarboxylate | TERT | 0.115736675 |
| bis((2S)-2-ethylhexyl) benzene-1,2-dicarboxylate | CTSV | 0.115736675 |
| bis((2S)-2-ethylhexyl) benzene-1,2-dicarboxylate | KCNN4 | 0.115736675 |
| bis((2S)-2-ethylhexyl) benzene-1,2-dicarboxylate | VCP | 0.115736675 |
| bis((2S)-2-ethylhexyl) benzene-1,2-dicarboxylate | MTNR1A | 0.115736675 |
| bis((2S)-2-ethylhexyl) benzene-1,2-dicarboxylate | MTNR1B | 0.115736675 |
| bis((2S)-2-ethylhexyl) benzene-1,2-dicarboxylate | CASP3 | 0.115736675 |
| bis((2S)-2-ethylhexyl) benzene-1,2-dicarboxylate | SLC6A9 | 0.115736675 |
| bis((2S)-2-ethylhexyl) benzene-1,2-dicarboxylate | PDE4A | 0.115736675 |
| bis((2S)-2-ethylhexyl) benzene-1,2-dicarboxylate | PDE4B | 0.115736675 |
| bis((2S)-2-ethylhexyl) benzene-1,2-dicarboxylate | ALOX15 | 0.115736675 |
| bis((2S)-2-ethylhexyl) benzene-1,2-dicarboxylate | PDE4C | 0.115736675 |
| bis((2S)-2-ethylhexyl) benzene-1,2-dicarboxylate | BAD | 0.115736675 |
| bis((2S)-2-ethylhexyl) benzene-1,2-dicarboxylate | PIK3CA | 0.115736675 |
| bis((2S)-2-ethylhexyl) benzene-1,2-dicarboxylate | MCL1 | 0.115736675 |
| bis((2S)-2-ethylhexyl) benzene-1,2-dicarboxylate | BCL2L1 | 0.115736675 |
| bis((2S)-2-ethylhexyl) benzene-1,2-dicarboxylate | BCL2L2 | 0.115736675 |
| bis((2S)-2-ethylhexyl) benzene-1,2-dicarboxylate | BCL2L10 | 0.115736675 |
| bis((2S)-2-ethylhexyl) benzene-1,2-dicarboxylate | BCL2A1 | 0.115736675 |
| bis((2S)-2-ethylhexyl) benzene-1,2-dicarboxylate | CRHR1 | 0.115736675 |
| bis((2S)-2-ethylhexyl) benzene-1,2-dicarboxylate | CDK5R1 CDK5 | 0.115736675 |
| bis((2S)-2-ethylhexyl) benzene-1,2-dicarboxylate | MAPK8 | 0.115736675 |
| bis((2S)-2-ethylhexyl) benzene-1,2-dicarboxylate | CCR1 | 0.115736675 |
| bis((2S)-2-ethylhexyl) benzene-1,2-dicarboxylate | APP | 0.115736675 |
| bis((2S)-2-ethylhexyl) benzene-1,2-dicarboxylate | PTAFR | 0.115736675 |
| bis((2S)-2-ethylhexyl) benzene-1,2-dicarboxylate | SCARB1 | 0.115736675 |
| bis((2S)-2-ethylhexyl) benzene-1,2-dicarboxylate | TDO2 | 0.115736675 |
| bis((2S)-2-ethylhexyl) benzene-1,2-dicarboxylate | GRM4 | 0.115736675 |
| bis((2S)-2-ethylhexyl) benzene-1,2-dicarboxylate | NMBR | 0.115736675 |
| bis((2S)-2-ethylhexyl) benzene-1,2-dicarboxylate | TACR3 | 0.115736675 |
| bis((2S)-2-ethylhexyl) benzene-1,2-dicarboxylate | IDO1 | 0.115736675 |
| Carthamidin | CYP19A1 | 0.344839366 |
| Carthamidin | CA7 | 0.254289912 |
| Carthamidin | CA12 | 0.254289912 |
| Carthamidin | CA4 | 0.254289912 |
| Carthamidin | CYP1B1 | 0.254289912 |
| Carthamidin | ESR1 | 0.254289912 |
| Carthamidin | MAOB | 0.20488198 |
| Carthamidin | PTGS1 | 0.196657136 |
| Carthamidin | HSD17B1 | 0.171978589 |
| Carthamidin | TAS2R31 | 0.171978589 |
| Carthamidin | ESR2 | 0.163737226 |
| Carthamidin | ABCC1 | 0.155528102 |
| Carthamidin | SHBG | 0.147256737 |
| Carthamidin | CBR1 | 0.147256737 |
| Carthamidin | ABCG2 | 0.130791955 |
| Carthamidin | ADORA1 | 0.106099949 |
| Carthamidin | ADORA3 | 0.097874534 |
| Carthamidin | AKR1C3 | 0.097874534 |
| Carthamidin | PLA2G1B | 0.097874534 |
| Carthamidin | MMP12 | 0.097874534 |
| Carthamidin | GRM5 | 0.097874534 |
| Carthamidin | CES1 | 0.097874534 |
| Carthamidin | PPARG | 0.097874534 |
| Carthamidin | CES2 | 0.097874534 |
| Carthamidin | MMP13 | 0.097874534 |
| Carthamidin | KLK1 | 0.097874534 |
| Carthamidin | KLK2 | 0.097874534 |
| Carthamidin | BACE1 | 0.097874534 |
| Carthamidin | SLC5A2 | 0.097874534 |
| Carthamidin | CHRNA7 | 0.097874534 |
| Carthamidin | SRC | 0.097874534 |
| Carthamidin | POLB | 0.097874534 |
| Carthamidin | PLA2G2A | 0.097874534 |
| Carthamidin | PLA2G5 | 0.097874534 |
| Carthamidin | PLA2G10 | 0.097874534 |
| Carthamidin | STS | 0.097874534 |
| Carthamidin | DYRK1A | 0.097874534 |
| Carthamidin | MAPT | 0.097874534 |
| Carthamidin | MAPK14 | 0.097874534 |
| Carthamidin | PGD | 0.097874534 |
| Carthamidin | BCL2 | 0.097874534 |
| Carthamidin | APP | 0.097874534 |
| Carthamidin | EDNRA | 0.097874534 |
| Carthamidin | TERT | 0.097874534 |
| Carthamidin | CA2 | 0.097874534 |
| Carthamidin | CA1 | 0.097874534 |
| Carthamidin | BCHE | 0.097874534 |
| Carthamidin | AKT1 | 0.097874534 |
| Carthamidin | CDK5R1 CDK5 | 0.097874534 |
| Carthamidin | CA6 | 0.097874534 |
| Carthamidin | CA13 | 0.097874534 |
| Carthamidin | CA5B | 0.097874534 |
| Carthamidin | CA5A | 0.097874534 |
| Carthamidin | RXRA | 0.097874534 |
| Carthamidin | CA9 | 0.097874534 |
| Carthamidin | ABCB1 | 0.097874534 |
| Carthamidin | MET | 0.097874534 |
| Carthamidin | KIT | 0.097874534 |
| Carthamidin | KDR | 0.097874534 |
| Carthamidin | FGFR1 | 0.097874534 |
| Carthamidin | NOX4 | 0.097874534 |
| Carthamidin | GRM2 | 0.097874534 |
| Carthamidin | ALOX12 | 0.097874534 |
| Carthamidin | HSD17B2 | 0.097874534 |
| Carthamidin | SERPINE1 | 0.097874534 |
| Carthamidin | NQO2 | 0.097874534 |
| Carthamidin | SNCA | 0.097874534 |
| Carthamidin | DNMT1 | 0.097874534 |
| Carthamidin | MMP2 | 0.097874534 |
| Carthamidin | MMP14 | 0.097874534 |
| Carthamidin | STAT1 | 0.097874534 |
| Carthamidin | SQLE | 0.097874534 |
| Carthamidin | IGF1R | 0.097874534 |
| Carthamidin | INSR | 0.097874534 |
| Carthamidin | FFAR1 | 0.097874534 |
| Carthamidin | CTSB | 0.097874534 |
| DIHYDROOROXYLIN | TAS2R31 | 0.22133019 |
| DIHYDROOROXYLIN | CBR1 | 0.122581769 |
| DIHYDROOROXYLIN | MAOB | 0.122581769 |
| DIHYDROOROXYLIN | CYP1B1 | 0.114337559 |
| DIHYDROOROXYLIN | CA12 | 0.114337559 |
| DIHYDROOROXYLIN | ADORA1 | 0.114337559 |
| DIHYDROOROXYLIN | ABCG2 | 0.114337559 |
| DIHYDROOROXYLIN | CA7 | 0.114337559 |
| DIHYDROOROXYLIN | ABCC1 | 0.106099949 |
| DIHYDROOROXYLIN | CYP19A1 | 0.106099949 |
| DIHYDROOROXYLIN | HSD17B1 | 0.106099949 |
| DIHYDROOROXYLIN | ESR1 | 0.106099949 |
| DIHYDROOROXYLIN | ESR2 | 0.106099949 |
| DIHYDROOROXYLIN | CA4 | 0.106099949 |
| DIHYDROOROXYLIN | SHBG | 0.106099949 |
| DIHYDROOROXYLIN | ADORA3 | 0.097874534 |
| DIHYDROOROXYLIN | AKR1C3 | 0.097874534 |
| DIHYDROOROXYLIN | PLA2G1B | 0.097874534 |
| DIHYDROOROXYLIN | MMP13 | 0.097874534 |
| DIHYDROOROXYLIN | PTGS1 | 0.097874534 |
| DIHYDROOROXYLIN | CA3 | 0.097874534 |
| DIHYDROOROXYLIN | MMP12 | 0.097874534 |
| DIHYDROOROXYLIN | KLK1 | 0.097874534 |
| DIHYDROOROXYLIN | KLK2 | 0.097874534 |
| DIHYDROOROXYLIN | ABCB1 | 0.097874534 |
| DIHYDROOROXYLIN | SLC5A2 | 0.097874534 |
| DIHYDROOROXYLIN | CA2 | 0.097874534 |
| DIHYDROOROXYLIN | CA1 | 0.097874534 |
| DIHYDROOROXYLIN | POLB | 0.097874534 |
| DIHYDROOROXYLIN | GRM5 | 0.097874534 |
| DIHYDROOROXYLIN | PLA2G5 | 0.097874534 |
| DIHYDROOROXYLIN | PLA2G10 | 0.097874534 |
| DIHYDROOROXYLIN | SRC | 0.097874534 |
| DIHYDROOROXYLIN | KDR | 0.097874534 |
| DIHYDROOROXYLIN | MAOA | 0.097874534 |
| DIHYDROOROXYLIN | CES2 | 0.097874534 |
| DIHYDROOROXYLIN | MAPT | 0.097874534 |
| DIHYDROOROXYLIN | DNMT1 | 0.097874534 |
| DIHYDROOROXYLIN | PGD | 0.097874534 |
| DIHYDROOROXYLIN | ST3GAL3 | 0.097874534 |
| DIHYDROOROXYLIN | FUT7 | 0.097874534 |
| DIHYDROOROXYLIN | FUT4 | 0.097874534 |
| DIHYDROOROXYLIN | STAT1 | 0.097874534 |
| DIHYDROOROXYLIN | MET | 0.097874534 |
| DIHYDROOROXYLIN | GUSB | 0.097874534 |
| DIHYDROOROXYLIN | RXRA | 0.097874534 |
| DIHYDROOROXYLIN | CA13 | 0.097874534 |
| DIHYDROOROXYLIN | FFAR1 | 0.097874534 |
| DIHYDROOROXYLIN | DAO | 0.097874534 |
| DIHYDROOROXYLIN | MPI | 0.097874534 |
| DIHYDROOROXYLIN | GSR | 0.097874534 |
| Diop | EGFR | 0.110612204 |
| Diop | SRC | 0.110612204 |
| epiberberine | SAE1 UBA2 | 0.825424897 |
| epiberberine | ACHE | 0.691501945 |
| epiberberine | BCHE | 0.281242088 |
| epiberberine | SIGMAR1 | 0.272973186 |
| epiberberine | HTR2B | 0.272973186 |
| epiberberine | ADRA2C | 0.24785052 |
| epiberberine | ADRA2B | 0.24785052 |
| epiberberine | CHRM1 | 0.24785052 |
| epiberberine | CYP2D6 | 0.24785052 |
| epiberberine | RAC1 | 0.10560828 |
| epiberberine | RPS6KB1 | 0.097239989 |
| epiberberine | AURKA | 0.097239989 |
| epiberberine | CDC42 | 0.097239989 |
| epiberberine | HPGD | 0.097239989 |
| epiberberine | TBXAS1 | 0.097239989 |
| epiberberine | MAOB | 0.097239989 |
| epiberberine | PIK3CG | 0.097239989 |
| epiberberine | GRK5 | 0.097239989 |
| epiberberine | CYP19A1 | 0.097239989 |
| epiberberine | SCD | 0.097239989 |
| epiberberine | AURKB | 0.097239989 |
| epiberberine | XBP1 | 0.097239989 |
| epiberberine | NTRK1 | 0.097239989 |
| epiberberine | PIK3CD | 0.097239989 |
| epiberberine | PIK3CB | 0.097239989 |
| epiberberine | GRIA1 | 0.097239989 |
| epiberberine | MAPKAPK2 | 0.097239989 |
| epiberberine | JAK2 | 0.097239989 |
| epiberberine | IMPDH2 | 0.097239989 |
| epiberberine | CHEK2 | 0.097239989 |
| epiberberine | TGM2 | 0.097239989 |
| epiberberine | CD38 | 0.097239989 |
| epiberberine | LCK | 0.097239989 |
| epiberberine | PIM1 | 0.097239989 |
| epiberberine | PIM2 | 0.097239989 |
| epiberberine | MAPK10 | 0.097239989 |
| epiberberine | MAP4K4 | 0.097239989 |
| epiberberine | PARP1 | 0.097239989 |
| epiberberine | F3 | 0.097239989 |
| epiberberine | TRPM8 | 0.097239989 |
| epiberberine | ABL1 | 0.097239989 |
| epiberberine | SIRT3 | 0.097239989 |
| epiberberine | SIRT2 | 0.097239989 |
| epiberberine | SIRT1 | 0.097239989 |
| epiberberine | NR3C2 | 0.097239989 |
| epiberberine | DHFR | 0.097239989 |
| epiberberine | FLT1 | 0.097239989 |
| epiberberine | ALOX5AP | 0.097239989 |
| epiberberine | ROCK1 | 0.097239989 |
| epiberberine | BCAT2 | 0.097239989 |
| epiberberine | PRKACA | 0.097239989 |
| epiberberine | CYP11B2 | 0.097239989 |
| epiberberine | ICAM1 | 0.097239989 |
| epiberberine | SELE | 0.097239989 |
| epiberberine | AGPAT2 | 0.097239989 |
| epiberberine | DRD4 | 0.097239989 |
| epiberberine | DRD3 | 0.097239989 |
| epiberberine | KIT | 0.097239989 |
| epiberberine | SRC | 0.097239989 |
| epiberberine | MKNK1 | 0.097239989 |
| epiberberine | FLT3 | 0.097239989 |
| epiberberine | IKBKB | 0.097239989 |
| epiberberine | ADORA2A | 0.097239989 |
| epiberberine | ADORA3 | 0.097239989 |
| epiberberine | PTPN1 | 0.097239989 |
| epiberberine | ZAP70 | 0.097239989 |
| epiberberine | SNCA | 0.097239989 |
| epiberberine | JAK3 | 0.097239989 |
| epiberberine | GSK3A | 0.097239989 |
| epiberberine | IRAK4 | 0.097239989 |
| epiberberine | MAPK1 | 0.097239989 |
| epiberberine | SLC22A12 | 0.097239989 |
| epiberberine | HTR3A | 0.097239989 |
| epiberberine | AKR1B1 | 0.097239989 |
| epiberberine | TYMS | 0.097239989 |
| epiberberine | CHEK1 | 0.097239989 |
| epiberberine | ERBB2 | 0.097239989 |
| epiberberine | MAPK8 | 0.097239989 |
| epiberberine | SCN9A | 0.097239989 |
| epiberberine | NPY5R | 0.097239989 |
| epiberberine | SLC5A1 | 0.097239989 |
| epiberberine | PDE4B | 0.097239989 |
| epiberberine | CCNE1 CDK2 | 0.097239989 |
| epiberberine | CYP11B1 | 0.097239989 |
| epiberberine | EPHA2 | 0.097239989 |
| epiberberine | FAAH | 0.097239989 |
| epiberberine | MST1R | 0.097239989 |
| epiberberine | DPP4 | 0.097239989 |
| epiberberine | ROCK2 | 0.097239989 |
| epiberberine | CCNC CDK8 | 0.097239989 |
| epiberberine | MET | 0.097239989 |
| epiberberine | STAT3 | 0.097239989 |
| epiberberine | AXL | 0.097239989 |
| epiberberine | CDK8 | 0.097239989 |
| epiberberine | MME | 0.097239989 |
| epiberberine | RPS6KA3 | 0.097239989 |
| epiberberine | EPHX2 | 0.097239989 |
| epiberberine | QPCT | 0.097239989 |
| epiberberine | MARS | 0.097239989 |
| epiberberine | MMP9 | 0.097239989 |
| Eriodyctiol (flavanone) | CYP19A1 | 0.40264338 |
| Eriodyctiol (flavanone) | CA7 | 0.40264338 |
| Eriodyctiol (flavanone) | CA12 | 0.40264338 |
| Eriodyctiol (flavanone) | CA4 | 0.40264338 |
| Eriodyctiol (flavanone) | CYP1B1 | 0.40264338 |
| Eriodyctiol (flavanone) | ESR1 | 0.262581506 |
| Eriodyctiol (flavanone) | ESR2 | 0.196657136 |
| Eriodyctiol (flavanone) | ABCC1 | 0.171978589 |
| Eriodyctiol (flavanone) | HSD17B1 | 0.171978589 |
| Eriodyctiol (flavanone) | SHBG | 0.171978589 |
| Eriodyctiol (flavanone) | CBR1 | 0.171978589 |
| Eriodyctiol (flavanone) | PTGS1 | 0.139061947 |
| Eriodyctiol (flavanone) | TAS2R31 | 0.106099949 |
| Eriodyctiol (flavanone) | ADORA3 | 0.106099949 |
| Eriodyctiol (flavanone) | ADORA1 | 0.106099949 |
| Eriodyctiol (flavanone) | MAOB | 0.106099949 |
| Eriodyctiol (flavanone) | MMP13 | 0.106099949 |
| Eriodyctiol (flavanone) | ABCG2 | 0.106099949 |
| Eriodyctiol (flavanone) | MMP12 | 0.097874534 |
| Eriodyctiol (flavanone) | AKR1C3 | 0.097874534 |
| Eriodyctiol (flavanone) | PLA2G1B | 0.097874534 |
| Eriodyctiol (flavanone) | ACHE | 0.097874534 |
| Eriodyctiol (flavanone) | KLK1 | 0.097874534 |
| Eriodyctiol (flavanone) | KLK2 | 0.097874534 |
| Eriodyctiol (flavanone) | GRM5 | 0.097874534 |
| Eriodyctiol (flavanone) | CES1 | 0.097874534 |
| Eriodyctiol (flavanone) | PPARG | 0.097874534 |
| Eriodyctiol (flavanone) | CES2 | 0.097874534 |
| Eriodyctiol (flavanone) | ABCB1 | 0.097874534 |
| Eriodyctiol (flavanone) | SLC5A2 | 0.097874534 |
| Eriodyctiol (flavanone) | MAOA | 0.097874534 |
| Eriodyctiol (flavanone) | POLB | 0.097874534 |
| Eriodyctiol (flavanone) | MAPT | 0.097874534 |
| Eriodyctiol (flavanone) | PLA2G2A | 0.097874534 |
| Eriodyctiol (flavanone) | PLA2G5 | 0.097874534 |
| Eriodyctiol (flavanone) | PLA2G10 | 0.097874534 |
| Eriodyctiol (flavanone) | BACE1 | 0.097874534 |
| Eriodyctiol (flavanone) | IGFBP3 | 0.097874534 |
| Eriodyctiol (flavanone) | EDNRA | 0.097874534 |
| Eriodyctiol (flavanone) | BCHE | 0.097874534 |
| Eriodyctiol (flavanone) | SRC | 0.097874534 |
| Eriodyctiol (flavanone) | CA2 | 0.097874534 |
| Eriodyctiol (flavanone) | SNCA | 0.097874534 |
| Eriodyctiol (flavanone) | DNMT1 | 0.097874534 |
| Eriodyctiol (flavanone) | KCNH2 | 0.097874534 |
| Eriodyctiol (flavanone) | MAPK14 | 0.097874534 |
| Eriodyctiol (flavanone) | MMP2 | 0.097874534 |
| Eriodyctiol (flavanone) | PGD | 0.097874534 |
| Eriodyctiol (flavanone) | ST3GAL3 | 0.097874534 |
| Eriodyctiol (flavanone) | FUT7 | 0.097874534 |
| Eriodyctiol (flavanone) | FUT4 | 0.097874534 |
| Eriodyctiol (flavanone) | STAT1 | 0.097874534 |
| Eriodyctiol (flavanone) | SQLE | 0.097874534 |
| Eriodyctiol (flavanone) | RXRA | 0.097874534 |
| Eriodyctiol (flavanone) | CHRNA7 | 0.097874534 |
| Eriodyctiol (flavanone) | CA1 | 0.097874534 |
| Eriodyctiol (flavanone) | GSK3B | 0.097874534 |
| Eriodyctiol (flavanone) | KIT | 0.097874534 |
| Eriodyctiol (flavanone) | KDR | 0.097874534 |
| Eriodyctiol (flavanone) | FGFR1 | 0.097874534 |
| Eriodyctiol (flavanone) | MET | 0.097874534 |
| Eriodyctiol (flavanone) | GRM2 | 0.097874534 |
| Eriodyctiol (flavanone) | MMP9 | 0.097874534 |
| Eriodyctiol (flavanone) | FFAR1 | 0.097874534 |
| Eriodyctiol (flavanone) | ALOX12 | 0.097874534 |
| Eriodyctiol (flavanone) | CA3 | 0.097874534 |
| Eriodyctiol (flavanone) | CA6 | 0.097874534 |
| Eriodyctiol (flavanone) | CA13 | 0.097874534 |
| Eriodyctiol (flavanone) | CA5B | 0.097874534 |
| Eriodyctiol (flavanone) | CA5A | 0.097874534 |
| Eriodyctiol (flavanone) | CA9 | 0.097874534 |
| Eriodyctiol (flavanone) | HIF1A | 0.097874534 |
| Eriodyctiol (flavanone) | CTSB | 0.097874534 |
| Eriodyctiol (flavanone) | APP | 0.097874534 |
| Eriodyctiol (flavanone) | SERPINE1 | 0.097874534 |
| Eriodyctiol (flavanone) | DYRK1A | 0.097874534 |
| Eriodyctiol (flavanone) | CTSL | 0.097874534 |
| Eriodyctiol (flavanone) | YWHAG | 0.097874534 |
| Eriodyctiol (flavanone) | ODC1 | 0.097874534 |
| Eriodyctiol (flavanone) | AKT1 | 0.097874534 |
| Moslosooflavone | AKR1B1 | 0.522277891 |
| Moslosooflavone | PTGS2 | 0.324775687 |
| Moslosooflavone | NOS2 | 0.214178827 |
| Moslosooflavone | ABCG2 | 0.206265233 |
| Moslosooflavone | CYP1A1 | 0.198389229 |
| Moslosooflavone | CYP1A2 | 0.198389229 |
| Moslosooflavone | CYP1B1 | 0.198389229 |
| Moslosooflavone | ABCB1 | 0.166798772 |
| Moslosooflavone | KIT | 0.158886034 |
| Moslosooflavone | ADORA1 | 0.15098181 |
| Moslosooflavone | OPRM1 | 0.135202128 |
| Moslosooflavone | OPRD1 | 0.12730257 |
| Moslosooflavone | PIM1 | 0.12730257 |
| Moslosooflavone | ALOX5 | 0.12730257 |
| Moslosooflavone | ADORA3 | 0.12730257 |
| Moslosooflavone | ADORA2A | 0.12730257 |
| Moslosooflavone | FLT3 | 0.119403562 |
| Moslosooflavone | PTPRS | 0.119403562 |
| Moslosooflavone | CYP19A1 | 0.111501865 |
| Moslosooflavone | PFKFB3 | 0.111501865 |
| Moslosooflavone | CA12 | 0.111501865 |
| Moslosooflavone | CA2 | 0.111501865 |
| Moslosooflavone | CA1 | 0.111501865 |
| Moslosooflavone | CA9 | 0.111501865 |
| Moslosooflavone | PLG | 0.111501865 |
| Moslosooflavone | TERT | 0.111501865 |
| Moslosooflavone | MCL1 | 0.111501865 |
| Moslosooflavone | MAOA | 0.111501865 |
| Moslosooflavone | EGFR | 0.111501865 |
| Moslosooflavone | MMP2 | 0.111501865 |
| Moslosooflavone | BCHE | 0.111501865 |
| Moslosooflavone | PARP1 | 0.111501865 |
| Moslosooflavone | ALOX15 | 0.111501865 |
| Moslosooflavone | IKBKB | 0.111501865 |
| Moslosooflavone | KDR | 0.111501865 |
| Moslosooflavone | GSK3B | 0.111501865 |
| Moslosooflavone | CDK6 | 0.111501865 |
| Moslosooflavone | CBR1 | 0.111501865 |
| Moslosooflavone | HSD17B2 | 0.111501865 |
| Moslosooflavone | HSD17B1 | 0.111501865 |
| Moslosooflavone | APP | 0.111501865 |
| Moslosooflavone | MAOB | 0.111501865 |
| Moslosooflavone | TNKS | 0.111501865 |
| Moslosooflavone | NTRK2 | 0.111501865 |
| Moslosooflavone | AMY1A | 0.111501865 |
| Moslosooflavone | ABCC1 | 0.111501865 |
| Moslosooflavone | CCNB3 CDK1 CCNB1 CCNB2 | 0.111501865 |
| Moslosooflavone | CA7 | 0.111501865 |
| Moslosooflavone | ESRRA | 0.111501865 |
| Moslosooflavone | NOX4 | 0.111501865 |
| Moslosooflavone | AR | 0.111501865 |
| Moslosooflavone | XDH | 0.111501865 |
| Moslosooflavone | CDK5R1 CDK5 | 0.111501865 |
| Moslosooflavone | ALOX12 | 0.111501865 |
| Moslosooflavone | PIK3CG | 0.111501865 |
| Moslosooflavone | CDK1 | 0.111501865 |
| Moslosooflavone | ESR2 | 0.111501865 |
| Moslosooflavone | MET | 0.111501865 |
| Moslosooflavone | MPG | 0.111501865 |
| Moslosooflavone | ACHE | 0.111501865 |
| Moslosooflavone | ESR1 | 0.111501865 |
| Moslosooflavone | BACE1 | 0.111501865 |
| Moslosooflavone | PIK3R1 | 0.111501865 |
| Moslosooflavone | SRC | 0.111501865 |
| Moslosooflavone | SYK | 0.111501865 |
| Moslosooflavone | GLO1 | 0.111501865 |
| Moslosooflavone | ARG1 | 0.111501865 |
| Moslosooflavone | MAPT | 0.111501865 |
| Moslosooflavone | PKN1 | 0.111501865 |
| Moslosooflavone | CAMK2B | 0.111501865 |
| Moslosooflavone | NEK6 | 0.111501865 |
| Moslosooflavone | PLA2G1B | 0.111501865 |
| Moslosooflavone | APEX1 | 0.111501865 |
| Moslosooflavone | AKR1C2 | 0.111501865 |
| Moslosooflavone | AKR1C1 | 0.111501865 |
| Moslosooflavone | AKR1C4 | 0.111501865 |
| Moslosooflavone | CA4 | 0.111501865 |
| Moslosooflavone | MMP3 | 0.111501865 |
| Moslosooflavone | MYLK | 0.111501865 |
| Moslosooflavone | CSNK2A1 | 0.111501865 |
| Moslosooflavone | MMP9 | 0.111501865 |
| Moslosooflavone | TUBB1 | 0.111501865 |
| Moslosooflavone | TUBB3 | 0.111501865 |
| Moslosooflavone | TNKS2 | 0.111501865 |
| Moslosooflavone | KDM5A | 0.111501865 |
| Moslosooflavone | GRK6 | 0.111501865 |
| Moslosooflavone | CTSD | 0.111501865 |
| Moslosooflavone | GUSB | 0.111501865 |
| Moslosooflavone | MPO | 0.111501865 |
| Moslosooflavone | KDM4E | 0.111501865 |
| Moslosooflavone | CA13 | 0.111501865 |
| Moslosooflavone | RET | 0.111501865 |
| Moslosooflavone | TTR | 0.111501865 |
| Moslosooflavone | PLK1 | 0.111501865 |
| Moslosooflavone | AURKB | 0.111501865 |
| Moslosooflavone | ODC1 | 0.111501865 |
| Moslosooflavone | LCK | 0.111501865 |
| Moslosooflavone | IGF1R | 0.111501865 |
| Moslosooflavone | CDK2 | 0.111501865 |
| Moslosooflavone | MMP13 | 0.111501865 |
| NEOBAICALEIN | AKR1B1 | 0.201278368 |
| NEOBAICALEIN | CYP1B1 | 0.153290397 |
| NEOBAICALEIN | KIT | 0.153290397 |
| NEOBAICALEIN | ODC1 | 0.14525066 |
| NEOBAICALEIN | ADORA1 | 0.14525066 |
| NEOBAICALEIN | ADORA2A | 0.137288883 |
| NEOBAICALEIN | ABCG2 | 0.12928422 |
| NEOBAICALEIN | PTGS2 | 0.121287003 |
| NEOBAICALEIN | ADORA3 | 0.121287003 |
| NEOBAICALEIN | ABCB1 | 0.121287003 |
| NEOBAICALEIN | MCL1 | 0.121287003 |
| NEOBAICALEIN | OPRM1 | 0.113285953 |
| NEOBAICALEIN | OPRD1 | 0.113285953 |
| NEOBAICALEIN | PLG | 0.113285953 |
| NEOBAICALEIN | ALOX5 | 0.113285953 |
| NEOBAICALEIN | NOS2 | 0.113285953 |
| NEOBAICALEIN | NOX4 | 0.113285953 |
| NEOBAICALEIN | GPR35 | 0.113285953 |
| NEOBAICALEIN | CA7 | 0.113285953 |
| NEOBAICALEIN | CA12 | 0.113285953 |
| NEOBAICALEIN | ESR2 | 0.113285953 |
| NEOBAICALEIN | FLT3 | 0.113285953 |
| NEOBAICALEIN | BCHE | 0.113285953 |
| NEOBAICALEIN | PTPRS | 0.113285953 |
| NEOBAICALEIN | MPG | 0.113285953 |
| NEOBAICALEIN | TERT | 0.113285953 |
| NEOBAICALEIN | HSD17B2 | 0.113285953 |
| NEOBAICALEIN | HSD17B1 | 0.113285953 |
| NEOBAICALEIN | APP | 0.113285953 |
| NEOBAICALEIN | ABCC1 | 0.113285953 |
| NEOBAICALEIN | CA2 | 0.113285953 |
| NEOBAICALEIN | AHR | 0.113285953 |
| NEOBAICALEIN | ESRRA | 0.113285953 |
| NEOBAICALEIN | MMP13 | 0.113285953 |
| NEOBAICALEIN | CA13 | 0.113285953 |
| NEOBAICALEIN | MMP9 | 0.113285953 |
| NEOBAICALEIN | MAOA | 0.113285953 |
| NEOBAICALEIN | MMP3 | 0.113285953 |
| NEOBAICALEIN | MMP2 | 0.113285953 |
| NEOBAICALEIN | PLA2G7 | 0.113285953 |
| NEOBAICALEIN | TYR | 0.113285953 |
| NEOBAICALEIN | XDH | 0.113285953 |
| NEOBAICALEIN | MAPT | 0.113285953 |
| NEOBAICALEIN | KDM4E | 0.113285953 |
| NEOBAICALEIN | AVPR2 | 0.113285953 |
| NEOBAICALEIN | TOP2A | 0.113285953 |
| NEOBAICALEIN | CYP19A1 | 0.113285953 |
| NEOBAICALEIN | GLO1 | 0.113285953 |
| NEOBAICALEIN | CA3 | 0.113285953 |
| NEOBAICALEIN | CAMK2B | 0.113285953 |
| NEOBAICALEIN | PLA2G1B | 0.113285953 |
| NEOBAICALEIN | APEX1 | 0.113285953 |
| NEOBAICALEIN | AKR1C2 | 0.113285953 |
| NEOBAICALEIN | AKR1C1 | 0.113285953 |
| NEOBAICALEIN | AKR1C3 | 0.113285953 |
| NEOBAICALEIN | AKR1C4 | 0.113285953 |
| NEOBAICALEIN | PIM1 | 0.113285953 |
| NEOBAICALEIN | CA1 | 0.113285953 |
| NEOBAICALEIN | CA9 | 0.113285953 |
| NEOBAICALEIN | KDR | 0.113285953 |
| NEOBAICALEIN | PLK1 | 0.113285953 |
| NEOBAICALEIN | EGFR | 0.113285953 |
| NEOBAICALEIN | GUSB | 0.113285953 |
| NEOBAICALEIN | ALOX15 | 0.113285953 |
| NEOBAICALEIN | ACHE | 0.113285953 |
| NEOBAICALEIN | AURKB | 0.113285953 |
| NEOBAICALEIN | NQO1 | 0.113285953 |
| NEOBAICALEIN | SRC | 0.113285953 |
| NEOBAICALEIN | PIK3CG | 0.113285953 |
| NEOBAICALEIN | CCNB3 CDK1 CCNB1 CCNB2 | 0.113285953 |
| NEOBAICALEIN | GSK3B | 0.113285953 |
| NEOBAICALEIN | BACE1 | 0.113285953 |
| NEOBAICALEIN | PFKFB3 | 0.113285953 |
| NEOBAICALEIN | MET | 0.113285953 |
| NEOBAICALEIN | AMY1A | 0.113285953 |
| NEOBAICALEIN | MPO | 0.113285953 |
| NEOBAICALEIN | RET | 0.113285953 |
| NEOBAICALEIN | MMP1 | 0.113285953 |
| NEOBAICALEIN | ADAM17 | 0.113285953 |
| NEOBAICALEIN | ERN1 | 0.113285953 |
| NEOBAICALEIN | CDK6 | 0.113285953 |
| NEOBAICALEIN | ARG1 | 0.113285953 |
| NEOBAICALEIN | PDGFRB | 0.113285953 |
| NEOBAICALEIN | HSP90AB1 | 0.113285953 |
| NEOBAICALEIN | CA4 | 0.113285953 |
| NEOBAICALEIN | MMP7 | 0.113285953 |
| NEOBAICALEIN | MMP8 | 0.113285953 |
| NEOBAICALEIN | CBR1 | 0.113285953 |
| NEOBAICALEIN | PCSK7 | 0.113285953 |
| NEOBAICALEIN | CYP2C9 | 0.113285953 |
| NEOBAICALEIN | DNM1 | 0.113285953 |
| NEOBAICALEIN | PPIA | 0.113285953 |
| NEOBAICALEIN | IDO1 | 0.113285953 |
| NEOBAICALEIN | CSNK2A1 | 0.113285953 |
| NEOBAICALEIN | CDK1 | 0.113285953 |
| NEOBAICALEIN | SLC29A1 | 0.113285953 |
| NEOBAICALEIN | GRK6 | 0.113285953 |
| NEOBAICALEIN | ADORA2B | 0.113285953 |
| NEOBAICALEIN | BMP1 | 0.113285953 |
| NEOBAICALEIN | DAPK1 | 0.113285953 |
| oroxylin a | P36888 | 1 |
| oroxylin a | P35228 | 0.6004118 |
| oroxylin a | P35354 | 0.270836923 |
| oroxylin a | P08183 | 0.22133019 |
| oroxylin a | P10721 | 0.196657136 |
| oroxylin a | P41143 | 0.196657136 |
| oroxylin a | B2RXH2 | 0.180252494 |
| oroxylin a | P47989 | 0.180252494 |
| oroxylin a | P16050 | 0.180252494 |
| oroxylin a | P06493 | 0.180252494 |
| oroxylin a | P18054 | 0.180252494 |
| oroxylin a | P43250 | 0.180252494 |
| oroxylin a | P11309 | 0.171978589 |
| oroxylin a | P30542 | 0.171978589 |
| oroxylin a | P29274 | 0.171978589 |
| oroxylin a | Q92731 | 0.139061947 |
| oroxylin a | P15121 | 0.139061947 |
| oroxylin a | P06239 | 0.130791955 |
| oroxylin a | Q13332 | 0.122581769 |
| oroxylin a | P03372 | 0.122581769 |
| oroxylin a | P0DMS8 | 0.122581769 |
| oroxylin a | Q16678 | 0.114337559 |
| oroxylin a | P14061 | 0.114337559 |
| oroxylin a | P21397 | 0.114337559 |
| oroxylin a | P43405 | 0.114337559 |
| oroxylin a | O60218 | 0.114337559 |
| oroxylin a | Q9UNQ0 | 0.114337559 |
| oroxylin a | P10275 | 0.114337559 |
| oroxylin a | P00747 | 0.106099949 |
| oroxylin a | Q15078 Q00535 | 0.106099949 |
| oroxylin a | P11511 | 0.106099949 |
| oroxylin a | P00918 | 0.106099949 |
| oroxylin a | Q8WWL7 P06493 P14635 O95067 | 0.106099949 |
| oroxylin a | P43166 | 0.106099949 |
| oroxylin a | Q00534 | 0.106099949 |
| oroxylin a | P00915 | 0.106099949 |
| oroxylin a | O43570 | 0.106099949 |
| oroxylin a | Q16790 | 0.106099949 |
| oroxylin a | P22748 | 0.106099949 |
| oroxylin a | P16152 | 0.106099949 |
| oroxylin a | O14920 | 0.106099949 |
| oroxylin a | Q16620 | 0.106099949 |
| oroxylin a | P11926 | 0.106099949 |
| oroxylin a | P39900 | 0.106099949 |
| oroxylin a | P28907 | 0.106099949 |
| oroxylin a | P11387 | 0.106099949 |
| oroxylin a | P05089 | 0.106099949 |
| oroxylin a | P14780 | 0.106099949 |
| oroxylin a | P08253 | 0.106099949 |
| oroxylin a | P48736 | 0.097874534 |
| oroxylin a | P49841 | 0.097874534 |
| oroxylin a | P33527 | 0.097874534 |
| oroxylin a | P62158 | 0.097874534 |
| oroxylin a | P14555 | 0.097874534 |
| oroxylin a | P22303 | 0.097874534 |
| oroxylin a | Q96S37 | 0.097874534 |
| oroxylin a | O14746 | 0.097874534 |
| oroxylin a | P04745 | 0.097874534 |
| oroxylin a | O95271 | 0.097874534 |
| oroxylin a | P02766 | 0.097874534 |
| oroxylin a | P37059 | 0.097874534 |
| oroxylin a | P14679 | 0.097874534 |
| oroxylin a | P35869 | 0.097874534 |
| oroxylin a | P11474 | 0.097874534 |
| oroxylin a | O76074 | 0.097874534 |
| oroxylin a | P05067 | 0.097874534 |
| oroxylin a | Q07820 | 0.097874534 |
| oroxylin a | Q13564 | 0.097874534 |
| oroxylin a | P00533 | 0.097874534 |
| oroxylin a | Q99720 | 0.097874534 |
| oroxylin a | Q9H2K2 | 0.097874534 |
| oroxylin a | Q9NPH5 | 0.097874534 |
| oroxylin a | P04798 | 0.097874534 |
| oroxylin a | P35372 | 0.097874534 |
| oroxylin a | P25024 | 0.097874534 |
| oroxylin a | P68400 | 0.097874534 |
| oroxylin a | P10636 | 0.097874534 |
| oroxylin a | P11388 | 0.097874534 |
| oroxylin a | P06213 | 0.097874534 |
| oroxylin a | Q15746 | 0.097874534 |
| oroxylin a | P05164 | 0.097874534 |
| oroxylin a | P27986 | 0.097874534 |
| oroxylin a | P53355 | 0.097874534 |
| oroxylin a | P06737 | 0.097874534 |
| oroxylin a | P45452 | 0.097874534 |
| oroxylin a | P08254 | 0.097874534 |
| oroxylin a | P07451 | 0.097874534 |
| oroxylin a | Q9ULX7 | 0.097874534 |
| oroxylin a | Q8N1Q1 | 0.097874534 |
| oroxylin a | P04054 | 0.097874534 |
| oroxylin a | P35218 | 0.097874534 |
| oroxylin a | P27695 | 0.097874534 |
| oroxylin a | P52895 | 0.097874534 |
| oroxylin a | Q04828 | 0.097874534 |
| oroxylin a | P42330 | 0.097874534 |
| oroxylin a | P17516 | 0.097874534 |
| oroxylin a | P14550 | 0.097874534 |
| oroxylin a | P29372 | 0.097874534 |
| oroxylin a | Q9HC97 | 0.097874534 |
| oroxylin a | P06276 | 0.097874534 |
| Salvigenin | AKR1B1 | 0.44383547 |
| Salvigenin | OPRD1 | 0.403466652 |
| Salvigenin | ADORA1 | 0.282326576 |
| Salvigenin | ADORA2A | 0.282326576 |
| Salvigenin | ADORA3 | 0.250033463 |
| Salvigenin | KIT | 0.225803139 |
| Salvigenin | ABCG2 | 0.225803139 |
| Salvigenin | PIM1 | 0.18543728 |
| Salvigenin | PTPRS | 0.136969565 |
| Salvigenin | CDK1 | 0.136969565 |
| Salvigenin | ALOX5 | 0.128898633 |
| Salvigenin | FLT3 | 0.128898633 |
| Salvigenin | MAOA | 0.120823672 |
| Salvigenin | PTGS2 | 0.120823672 |
| Salvigenin | AMY1A | 0.120823672 |
| Salvigenin | GRK6 | 0.120823672 |
| Salvigenin | ALOX15 | 0.120823672 |
| Salvigenin | OPRM1 | 0.120823672 |
| Salvigenin | ABCB1 | 0.120823672 |
| Salvigenin | SYK | 0.112748418 |
| Salvigenin | MMP9 | 0.112748418 |
| Salvigenin | MMP2 | 0.112748418 |
| Salvigenin | ABCC1 | 0.112748418 |
| Salvigenin | HSD17B1 | 0.112748418 |
| Salvigenin | KDM4E | 0.112748418 |
| Salvigenin | AKR1B10 | 0.112748418 |
| Salvigenin | ACHE | 0.112748418 |
| Salvigenin | ESR1 | 0.112748418 |
| Salvigenin | ESR2 | 0.112748418 |
| Salvigenin | APP | 0.112748418 |
| Salvigenin | CYP1B1 | 0.112748418 |
| Salvigenin | GSK3B | 0.104671941 |
| Salvigenin | NOX4 | 0.104671941 |
| Salvigenin | LCK | 0.104671941 |
| Salvigenin | MMP12 | 0.104671941 |
| Salvigenin | PLG | 0.104671941 |
| Salvigenin | CBR1 | 0.104671941 |
| Salvigenin | GLO1 | 0.104671941 |
| Salvigenin | ARG1 | 0.104671941 |
| Salvigenin | CCNB3 CDK1 CCNB1 CCNB2 | 0.104671941 |
| Salvigenin | MAOB | 0.104671941 |
| Salvigenin | CA2 | 0.104671941 |
| Salvigenin | CA1 | 0.104671941 |
| Salvigenin | CA9 | 0.104671941 |
| Salvigenin | NOS2 | 0.104671941 |
| Salvigenin | XDH | 0.104671941 |
| Salvigenin | CDK2 | 0.104671941 |
| Salvigenin | PARP1 | 0.104671941 |
| Salvigenin | CDK5R1 CDK5 | 0.104671941 |
| Salvigenin | CA12 | 0.104671941 |
| Salvigenin | HSD17B2 | 0.104671941 |
| Salvigenin | CA7 | 0.104671941 |
| Salvigenin | MCL1 | 0.104671941 |
| Salvigenin | TERT | 0.104671941 |
| Salvigenin | AHR | 0.104671941 |
| Salvigenin | ESRRA | 0.104671941 |
| Salvigenin | NAE1 | 0.104671941 |
| Salvigenin | MMP13 | 0.104671941 |
| Salvigenin | MMP3 | 0.104671941 |
| Salvigenin | AURKB | 0.104671941 |
| Salvigenin | CSNK2A1 | 0.104671941 |
| Salvigenin | MET | 0.104671941 |
| Salvigenin | AR | 0.104671941 |
| Salvigenin | TNKS | 0.104671941 |
| Salvigenin | CYP19A1 | 0.104671941 |
| Salvigenin | SRC | 0.104671941 |
| Salvigenin | PFKFB3 | 0.104671941 |
| Salvigenin | BACE1 | 0.104671941 |
| Salvigenin | CA4 | 0.104671941 |
| Salvigenin | KDR | 0.104671941 |
| Salvigenin | TTR | 0.104671941 |
| Salvigenin | GPR35 | 0.104671941 |
| Salvigenin | MPO | 0.104671941 |
| Salvigenin | CA3 | 0.104671941 |
| Salvigenin | PKN1 | 0.104671941 |
| Salvigenin | NEK6 | 0.104671941 |
| Salvigenin | APEX1 | 0.104671941 |
| Salvigenin | AKR1C2 | 0.104671941 |
| Salvigenin | AKR1C1 | 0.104671941 |
| Salvigenin | AKR1C4 | 0.104671941 |
| Salvigenin | AKR1A1 | 0.104671941 |
| Salvigenin | BCHE | 0.104671941 |
| Salvigenin | TNKS2 | 0.104671941 |
| Salvigenin | MPG | 0.104671941 |
| Salvigenin | SLC22A12 | 0.104671941 |
| Salvigenin | ALOX12 | 0.104671941 |
| Salvigenin | CALM1 | 0.104671941 |
| Salvigenin | EGFR | 0.104671941 |
| Salvigenin | ST6GAL1 | 0.104671941 |
| Salvigenin | CDK6 | 0.104671941 |
| Salvigenin | CFTR | 0.104671941 |
| Salvigenin | PIK3CG | 0.104671941 |
| Salvigenin | SIGMAR1 | 0.104671941 |
| Salvigenin | ODC1 | 0.104671941 |
| Salvigenin | PTPN1 | 0.104671941 |
| Salvigenin | IKBKB | 0.104671941 |
| Salvigenin | PLK1 | 0.104671941 |
| Salvigenin | CA13 | 0.104671941 |
| Salvigenin | CA6 | 0.104671941 |
| Salvigenin | CA14 | 0.104671941 |
| Skullcapflavone II | AKR1B1 | 0.201278368 |
| Skullcapflavone II | CYP1B1 | 0.153290397 |
| Skullcapflavone II | KIT | 0.153290397 |
| Skullcapflavone II | ODC1 | 0.14525066 |
| Skullcapflavone II | ADORA1 | 0.14525066 |
| Skullcapflavone II | ADORA2A | 0.137288883 |
| Skullcapflavone II | ABCG2 | 0.12928422 |
| Skullcapflavone II | PTGS2 | 0.121287003 |
| Skullcapflavone II | ADORA3 | 0.121287003 |
| Skullcapflavone II | ABCB1 | 0.121287003 |
| Skullcapflavone II | MCL1 | 0.121287003 |
| Skullcapflavone II | OPRM1 | 0.113285953 |
| Skullcapflavone II | OPRD1 | 0.113285953 |
| Skullcapflavone II | PLG | 0.113285953 |
| Skullcapflavone II | ALOX5 | 0.113285953 |
| Skullcapflavone II | NOS2 | 0.113285953 |
| Skullcapflavone II | NOX4 | 0.113285953 |
| Skullcapflavone II | GPR35 | 0.113285953 |
| Skullcapflavone II | CA7 | 0.113285953 |
| Skullcapflavone II | CA12 | 0.113285953 |
| Skullcapflavone II | ESR2 | 0.113285953 |
| Skullcapflavone II | FLT3 | 0.113285953 |
| Skullcapflavone II | BCHE | 0.113285953 |
| Skullcapflavone II | PTPRS | 0.113285953 |
| Skullcapflavone II | MPG | 0.113285953 |
| Skullcapflavone II | TERT | 0.113285953 |
| Skullcapflavone II | HSD17B2 | 0.113285953 |
| Skullcapflavone II | HSD17B1 | 0.113285953 |
| Skullcapflavone II | APP | 0.113285953 |
| Skullcapflavone II | ABCC1 | 0.113285953 |
| Skullcapflavone II | CA2 | 0.113285953 |
| Skullcapflavone II | AHR | 0.113285953 |
| Skullcapflavone II | ESRRA | 0.113285953 |
| Skullcapflavone II | MMP13 | 0.113285953 |
| Skullcapflavone II | CA13 | 0.113285953 |
| Skullcapflavone II | MMP9 | 0.113285953 |
| Skullcapflavone II | MAOA | 0.113285953 |
| Skullcapflavone II | MMP3 | 0.113285953 |
| Skullcapflavone II | MMP2 | 0.113285953 |
| Skullcapflavone II | PLA2G7 | 0.113285953 |
| Skullcapflavone II | TYR | 0.113285953 |
| Skullcapflavone II | XDH | 0.113285953 |
| Skullcapflavone II | MAPT | 0.113285953 |
| Skullcapflavone II | KDM4E | 0.113285953 |
| Skullcapflavone II | AVPR2 | 0.113285953 |
| Skullcapflavone II | TOP2A | 0.113285953 |
| Skullcapflavone II | CYP19A1 | 0.113285953 |
| Skullcapflavone II | GLO1 | 0.113285953 |
| Skullcapflavone II | CA3 | 0.113285953 |
| Skullcapflavone II | CAMK2B | 0.113285953 |
| Skullcapflavone II | PLA2G1B | 0.113285953 |
| Skullcapflavone II | APEX1 | 0.113285953 |
| Skullcapflavone II | AKR1C2 | 0.113285953 |
| Skullcapflavone II | AKR1C1 | 0.113285953 |
| Skullcapflavone II | AKR1C3 | 0.113285953 |
| Skullcapflavone II | AKR1C4 | 0.113285953 |
| Skullcapflavone II | PIM1 | 0.113285953 |
| Skullcapflavone II | CA1 | 0.113285953 |
| Skullcapflavone II | CA9 | 0.113285953 |
| Skullcapflavone II | KDR | 0.113285953 |
| Skullcapflavone II | PLK1 | 0.113285953 |
| Skullcapflavone II | EGFR | 0.113285953 |
| Skullcapflavone II | GUSB | 0.113285953 |
| Skullcapflavone II | ALOX15 | 0.113285953 |
| Skullcapflavone II | ACHE | 0.113285953 |
| Skullcapflavone II | AURKB | 0.113285953 |
| Skullcapflavone II | NQO1 | 0.113285953 |
| Skullcapflavone II | SRC | 0.113285953 |
| Skullcapflavone II | PIK3CG | 0.113285953 |
| Skullcapflavone II | CCNB3 CDK1 CCNB1 CCNB2 | 0.113285953 |
| Skullcapflavone II | GSK3B | 0.113285953 |
| Skullcapflavone II | BACE1 | 0.113285953 |
| Skullcapflavone II | PFKFB3 | 0.113285953 |
| Skullcapflavone II | MET | 0.113285953 |
| Skullcapflavone II | AMY1A | 0.113285953 |
| Skullcapflavone II | MPO | 0.113285953 |
| Skullcapflavone II | RET | 0.113285953 |
| Skullcapflavone II | MMP1 | 0.113285953 |
| Skullcapflavone II | ADAM17 | 0.113285953 |
| Skullcapflavone II | ERN1 | 0.113285953 |
| Skullcapflavone II | CDK6 | 0.113285953 |
| Skullcapflavone II | ARG1 | 0.113285953 |
| Skullcapflavone II | PDGFRB | 0.113285953 |
| Skullcapflavone II | HSP90AB1 | 0.113285953 |
| Skullcapflavone II | CA4 | 0.113285953 |
| Skullcapflavone II | MMP7 | 0.113285953 |
| Skullcapflavone II | MMP8 | 0.113285953 |
| Skullcapflavone II | CBR1 | 0.113285953 |
| Skullcapflavone II | PCSK7 | 0.113285953 |
| Skullcapflavone II | CYP2C9 | 0.113285953 |
| Skullcapflavone II | DNM1 | 0.113285953 |
| Skullcapflavone II | PPIA | 0.113285953 |
| Skullcapflavone II | IDO1 | 0.113285953 |
| Skullcapflavone II | CSNK2A1 | 0.113285953 |
| Skullcapflavone II | CDK1 | 0.113285953 |
| Skullcapflavone II | SLC29A1 | 0.113285953 |
| Skullcapflavone II | GRK6 | 0.113285953 |
| Skullcapflavone II | ADORA2B | 0.113285953 |
| Skullcapflavone II | BMP1 | 0.113285953 |
| Skullcapflavone II | DAPK1 | 0.113285953 |
| Stigmasterol | NPC1L1 | 0.912575154 |
| Stigmasterol | NR1H3 | 0.589276033 |
| Stigmasterol | RORC | 0.472708109 |
| Stigmasterol | SHBG | 0.45601846 |
| Stigmasterol | CYP51A1 | 0.45601846 |
| Stigmasterol | HMGCR | 0.414383452 |
| Stigmasterol | SREBF2 | 0.364410487 |
| Stigmasterol | CYP19A1 | 0.314405075 |
| Stigmasterol | AR | 0.239432052 |
| Stigmasterol | CYP17A1 | 0.189458322 |
| Stigmasterol | RORA | 0.172809876 |
| Stigmasterol | ESR1 | 0.16447208 |
| Stigmasterol | ESR2 | 0.16447208 |
| Stigmasterol | SLC6A2 | 0.131155749 |
| Stigmasterol | CYP2C19 | 0.131155749 |
| Stigmasterol | BCHE | 0.11449479 |
| Stigmasterol | PTPN1 | 0.11449479 |
| Stigmasterol | SERPINA6 | 0.11449479 |
| Stigmasterol | G6PD | 0.11449479 |
| Stigmasterol | ACHE | 0.106165761 |
| Stigmasterol | SLC6A4 | 0.106165761 |
| Stigmasterol | NR1I3 | 0.106165761 |
| Stigmasterol | CHRM2 | 0.106165761 |
| Stigmasterol | PTGER1 | 0.106165761 |
| Stigmasterol | PTGER2 | 0.106165761 |
| Stigmasterol | TBXAS1 | 0.106165761 |
| Stigmasterol | PTGES | 0.106165761 |
| Stigmasterol | NR1H2 | 0.106165761 |
| Stigmasterol | PPARA | 0.106165761 |
| Stigmasterol | PPARD | 0.106165761 |
| Stigmasterol | VDR | 0.106165761 |
| Stigmasterol | SQLE | 0.106165761 |
| Stigmasterol | PTPN6 | 0.106165761 |
| Stigmasterol | PTPN2 | 0.106165761 |
| Stigmasterol | FDFT1 | 0.106165761 |
| Stigmasterol | HSD11B1 | 0.106165761 |
| Stigmasterol | DHCR7 | 0.106165761 |
| Stigmasterol | GLRA1 | 0.106165761 |
| Stigmasterol | PPARG | 0.106165761 |
| Stigmasterol | NOS2 | 0.106165761 |
| Stigmasterol | UGT2B7 | 0.106165761 |
| Stigmasterol | POLB | 0.106165761 |
| Panicolin | PTGS2 | 0.201601849 |
| Panicolin | AKR1B1 | 0.176593625 |
| Panicolin | NOS2 | 0.134939009 |
| Panicolin | CYP1B1 | 0.118277085 |
| Panicolin | OPRM1 | 0.118277085 |
| Panicolin | OPRD1 | 0.118277085 |
| Panicolin | ADORA1 | 0.118277085 |
| Panicolin | ADORA2A | 0.118277085 |
| Panicolin | ABCG2 | 0.10994577 |
| Panicolin | ALOX5 | 0.10994577 |
| Panicolin | MCL1 | 0.10994577 |
| Panicolin | ODC1 | 0.10994577 |
| Panicolin | KIT | 0.101613855 |
| Panicolin | TERT | 0.101613855 |
| Panicolin | BCHE | 0.101613855 |
| Panicolin | MAOA | 0.101613855 |
| Panicolin | CA2 | 0.101613855 |
| Panicolin | MMP13 | 0.101613855 |
| Panicolin | PTPRS | 0.101613855 |
| Panicolin | DAPK1 | 0.101613855 |
| Panicolin | MPG | 0.101613855 |
| Panicolin | FLT3 | 0.101613855 |
| Panicolin | ABCB1 | 0.101613855 |
| Panicolin | EGFR | 0.101613855 |
| Panicolin | KDR | 0.101613855 |
| Panicolin | ESR2 | 0.101613855 |
| Panicolin | HSD17B2 | 0.101613855 |
| Panicolin | HSD17B1 | 0.101613855 |
| Panicolin | GPR35 | 0.101613855 |
| Panicolin | MMP9 | 0.101613855 |
| Panicolin | MMP3 | 0.101613855 |
| Panicolin | CA1 | 0.101613855 |
| Panicolin | MMP2 | 0.101613855 |
| Panicolin | AHR | 0.101613855 |
| Panicolin | ADORA3 | 0.101613855 |
| Panicolin | NOX4 | 0.101613855 |
| Panicolin | ALOX15 | 0.101613855 |
| Panicolin | GSK3B | 0.101613855 |
| Panicolin | CA7 | 0.101613855 |
| Panicolin | CA12 | 0.101613855 |
| Panicolin | XDH | 0.101613855 |
| Panicolin | NTRK2 | 0.101613855 |
| Panicolin | APP | 0.101613855 |
| Panicolin | PIK3CG | 0.101613855 |
| Panicolin | MET | 0.101613855 |
| Panicolin | CYP19A1 | 0.101613855 |
| Panicolin | TYR | 0.101613855 |
| Panicolin | SRC | 0.101613855 |
| Panicolin | PIM1 | 0.101613855 |
| Panicolin | PLK1 | 0.101613855 |
| Panicolin | CA9 | 0.101613855 |
| Panicolin | KDM4E | 0.101613855 |
| Panicolin | AVPR2 | 0.101613855 |
| Panicolin | TOP2A | 0.101613855 |
| Panicolin | GLO1 | 0.101613855 |
| Panicolin | CA3 | 0.101613855 |
| Panicolin | PKN1 | 0.101613855 |
| Panicolin | CAMK2B | 0.101613855 |
| Panicolin | NEK6 | 0.101613855 |
| Panicolin | PLA2G1B | 0.101613855 |
| Panicolin | AXL | 0.101613855 |
| Panicolin | APEX1 | 0.101613855 |
| Panicolin | NUAK1 | 0.101613855 |
| Panicolin | AKR1C2 | 0.101613855 |
| Panicolin | AKR1C1 | 0.101613855 |
| Panicolin | AKR1C3 | 0.101613855 |
| Panicolin | AKR1C4 | 0.101613855 |
| Panicolin | PFKFB3 | 0.101613855 |
| Panicolin | BACE1 | 0.101613855 |
| Panicolin | CDK5R1 CDK5 | 0.101613855 |
| Panicolin | CCNB3 CDK1 CCNB1 CCNB2 | 0.101613855 |
| Panicolin | CDK6 | 0.101613855 |
| Panicolin | CBR1 | 0.101613855 |
| Panicolin | TNKS | 0.101613855 |
| Panicolin | MAOB | 0.101613855 |
| Panicolin | AMY1A | 0.101613855 |
| Panicolin | MMP1 | 0.101613855 |
| Panicolin | ABCC1 | 0.101613855 |
| Panicolin | GRK6 | 0.101613855 |
| Panicolin | ACHE | 0.101613855 |
| Panicolin | CA14 | 0.101613855 |
| Panicolin | CSNK2A1 | 0.101613855 |
| Panicolin | GUSB | 0.101613855 |
| Panicolin | MMP12 | 0.101613855 |
| Panicolin | MYLK | 0.101613855 |
| Panicolin | CDK2 | 0.101613855 |
| Panicolin | CYP1A1 | 0.101613855 |
| Panicolin | CYP1A2 | 0.101613855 |
| Panicolin | ALK | 0.101613855 |
| Panicolin | PLA2G7 | 0.101613855 |
| Panicolin | CDK1 | 0.101613855 |
| Panicolin | PDE5A | 0.101613855 |
| Panicolin | ARG1 | 0.101613855 |
| Panicolin | F2 | 0.101613855 |
| Panicolin | ELANE | 0.101613855 |
| Panicolin | FLT1 | 0.101613855 |
| Panicolin | PDGFRB | 0.101613855 |
| Panicolin | FLT4 | 0.101613855 |
| Panicolin | CTSV | 0.101613855 |
| Panicolin | IGF1R | 0.101613855 |
| Norwogonin | KDM4E | 0.592041374 |
| Norwogonin | XDH | 0.592041374 |
| Norwogonin | ALOX15 | 0.592041374 |
| Norwogonin | CDK1 | 0.592041374 |
| Norwogonin | ALOX12 | 0.592041374 |
| Norwogonin | GRK6 | 0.592041374 |
| Norwogonin | IKBKB | 0.411906406 |
| Norwogonin | NTRK2 | 0.411906406 |
| Norwogonin | TERT | 0.25622784 |
| Norwogonin | CYP19A1 | 0.231637185 |
| Norwogonin | PFKFB3 | 0.231637185 |
| Norwogonin | HSD17B1 | 0.207053974 |
| Norwogonin | HSD17B2 | 0.207053974 |
| Norwogonin | ABCB1 | 0.190656977 |
| Norwogonin | AKR1B1 | 0.190656977 |
| Norwogonin | CDK5R1 CDK5 | 0.190656977 |
| Norwogonin | CA2 | 0.190656977 |
| Norwogonin | CCNB3 CDK1 CCNB1 CCNB2 | 0.190656977 |
| Norwogonin | CA7 | 0.190656977 |
| Norwogonin | CDK6 | 0.190656977 |
| Norwogonin | CA1 | 0.190656977 |
| Norwogonin | CA12 | 0.190656977 |
| Norwogonin | CA9 | 0.190656977 |
| Norwogonin | CA4 | 0.190656977 |
| Norwogonin | CYP1B1 | 0.190656977 |
| Norwogonin | ABCG2 | 0.190656977 |
| Norwogonin | CBR1 | 0.190656977 |
| Norwogonin | BCHE | 0.174270753 |
| Norwogonin | ACHE | 0.174270753 |
| Norwogonin | ADORA1 | 0.174270753 |
| Norwogonin | ADORA2A | 0.174270753 |
| Norwogonin | ADORA3 | 0.174270753 |
| Norwogonin | PTGS2 | 0.166097445 |
| Norwogonin | NOS2 | 0.166097445 |
| Norwogonin | ESR2 | 0.157929217 |
| Norwogonin | ESR1 | 0.149732594 |
| Norwogonin | FLT3 | 0.141522086 |
| Norwogonin | PTPRS | 0.125142649 |
| Norwogonin | AMY1A | 0.125142649 |
| Norwogonin | AR | 0.125142649 |
| Norwogonin | NOX4 | 0.125142649 |
| Norwogonin | MAOA | 0.125142649 |
| Norwogonin | SYK | 0.125142649 |
| Norwogonin | GSK3B | 0.125142649 |
| Norwogonin | ABCC1 | 0.125142649 |
| Norwogonin | TTR | 0.125142649 |
| Norwogonin | CSNK2A1 | 0.125142649 |
| Norwogonin | CFTR | 0.125142649 |
| Norwogonin | AKR1B10 | 0.125142649 |
| Norwogonin | TNKS2 | 0.125142649 |
| Norwogonin | TNKS | 0.125142649 |
| Norwogonin | EGFR | 0.116965063 |
| Norwogonin | SRC | 0.116965063 |
| Norwogonin | ALOX5 | 0.116965063 |
| Norwogonin | PIM1 | 0.116965063 |
| Norwogonin | LCK | 0.116965063 |
| Norwogonin | TYR | 0.116965063 |
| Norwogonin | AHR | 0.116965063 |
| Norwogonin | ESRRA | 0.116965063 |
| Norwogonin | AURKB | 0.116965063 |
| Norwogonin | PIK3CG | 0.108770969 |
| Norwogonin | MAPT | 0.108770969 |
| Norwogonin | TOP2A | 0.108770969 |
| Norwogonin | IGF1R | 0.108770969 |
| Norwogonin | INSR | 0.108770969 |
| Norwogonin | MYLK | 0.108770969 |
| Norwogonin | APEX1 | 0.108770969 |
| Norwogonin | PARP1 | 0.108770969 |
| Norwogonin | SLC22A12 | 0.108770969 |
| Norwogonin | GPR35 | 0.108770969 |
| Norwogonin | APP | 0.108770969 |
| Norwogonin | KDR | 0.108770969 |
| Norwogonin | PLK1 | 0.108770969 |
| Norwogonin | MET | 0.108770969 |
| Norwogonin | ALK | 0.108770969 |
| Norwogonin | AXL | 0.108770969 |
| Norwogonin | ARG1 | 0.108770969 |
| Norwogonin | KIT | 0.100578902 |
| Norwogonin | GLO1 | 0.100578902 |
| Norwogonin | MMP9 | 0.100578902 |
| Norwogonin | MMP2 | 0.100578902 |
| Norwogonin | MMP12 | 0.100578902 |
| Norwogonin | CD38 | 0.100578902 |
| Norwogonin | TOP1 | 0.100578902 |
| Norwogonin | CALM1 | 0.100578902 |
| Norwogonin | BACE1 | 0.100578902 |
| Norwogonin | NAE1 | 0.100578902 |
| Norwogonin | CA6 | 0.100578902 |
| Norwogonin | CXCR1 | 0.100578902 |
| Norwogonin | CYP1A1 | 0.100578902 |
| Norwogonin | F2 | 0.100578902 |
| Norwogonin | CDK2 | 0.100578902 |
| Norwogonin | DAPK1 | 0.100578902 |
| Norwogonin | MPG | 0.100578902 |
| Norwogonin | PTPN1 | 0.100578902 |
| Norwogonin | AVPR2 | 0.100578902 |
| Norwogonin | DRD4 | 0.100578902 |
| Norwogonin | MPO | 0.100578902 |
| Norwogonin | PIK3R1 | 0.100578902 |
| Norwogonin | PYGL | 0.100578902 |
| 11,13-Eicosadienoic acid, methyl ester | CES2 | 0.101613855 |
| 11,13-Eicosadienoic acid, methyl ester | TOP2A | 0.101613855 |
| 11,13-Eicosadienoic acid, methyl ester | NR1H3 | 0.101613855 |
| 11,13-Eicosadienoic acid, methyl ester | PPARG | 0.101613855 |
| 11,13-Eicosadienoic acid, methyl ester | FAAH | 0.101613855 |
| 11,13-Eicosadienoic acid, methyl ester | FABP4 | 0.101613855 |
| 11,13-Eicosadienoic acid, methyl ester | PPARA | 0.101613855 |
| 11,13-Eicosadienoic acid, methyl ester | TERT | 0.101613855 |
| 11,13-Eicosadienoic acid, methyl ester | FABP3 | 0.101613855 |
| 11,13-Eicosadienoic acid, methyl ester | FABP5 | 0.101613855 |
| 11,13-Eicosadienoic acid, methyl ester | FABP1 | 0.101613855 |
| 11,13-Eicosadienoic acid, methyl ester | ACACB | 0.101613855 |
| 11,13-Eicosadienoic acid, methyl ester | PTGER2 | 0.101613855 |
| 11,13-Eicosadienoic acid, methyl ester | VDR | 0.101613855 |
| 11,13-Eicosadienoic acid, methyl ester | PTGIR | 0.101613855 |
| 11,13-Eicosadienoic acid, methyl ester | SCD | 0.101613855 |
| 11,13-Eicosadienoic acid, methyl ester | MMP13 | 0.101613855 |
| 11,13-Eicosadienoic acid, methyl ester | MGLL | 0.101613855 |
| 11,13-Eicosadienoic acid, methyl ester | PTGS2 | 0.101613855 |
| dihydrooroxylin A | TAS2R31 | 0.22133019 |
| dihydrooroxylin A | CBR1 | 0.122581769 |
| dihydrooroxylin A | MAOB | 0.122581769 |
| dihydrooroxylin A | CYP1B1 | 0.114337559 |
| dihydrooroxylin A | CA12 | 0.114337559 |
| dihydrooroxylin A | ADORA1 | 0.114337559 |
| dihydrooroxylin A | ABCG2 | 0.114337559 |
| dihydrooroxylin A | CA7 | 0.114337559 |
| dihydrooroxylin A | ABCC1 | 0.106099949 |
| dihydrooroxylin A | CYP19A1 | 0.106099949 |
| dihydrooroxylin A | HSD17B1 | 0.106099949 |
| dihydrooroxylin A | ESR1 | 0.106099949 |
| dihydrooroxylin A | ESR2 | 0.106099949 |
| dihydrooroxylin A | CA4 | 0.106099949 |
| dihydrooroxylin A | SHBG | 0.106099949 |
| dihydrooroxylin A | ADORA3 | 0.097874534 |
| dihydrooroxylin A | AKR1C3 | 0.097874534 |
| dihydrooroxylin A | PLA2G1B | 0.097874534 |
| dihydrooroxylin A | MMP13 | 0.097874534 |
| dihydrooroxylin A | PTGS1 | 0.097874534 |
| dihydrooroxylin A | CA3 | 0.097874534 |
| dihydrooroxylin A | MMP12 | 0.097874534 |
| dihydrooroxylin A | KLK1 | 0.097874534 |
| dihydrooroxylin A | KLK2 | 0.097874534 |
| dihydrooroxylin A | ABCB1 | 0.097874534 |
| dihydrooroxylin A | SLC5A2 | 0.097874534 |
| dihydrooroxylin A | CA2 | 0.097874534 |
| dihydrooroxylin A | CA1 | 0.097874534 |
| dihydrooroxylin A | POLB | 0.097874534 |
| dihydrooroxylin A | GRM5 | 0.097874534 |
| dihydrooroxylin A | PLA2G5 | 0.097874534 |
| dihydrooroxylin A | PLA2G10 | 0.097874534 |
| dihydrooroxylin A | SRC | 0.097874534 |
| dihydrooroxylin A | KDR | 0.097874534 |
| dihydrooroxylin A | MAOA | 0.097874534 |
| dihydrooroxylin A | CES2 | 0.097874534 |
| dihydrooroxylin A | MAPT | 0.097874534 |
| dihydrooroxylin A | DNMT1 | 0.097874534 |
| dihydrooroxylin A | PGD | 0.097874534 |
| dihydrooroxylin A | ST3GAL3 | 0.097874534 |
| dihydrooroxylin A | FUT7 | 0.097874534 |
| dihydrooroxylin A | FUT4 | 0.097874534 |
| dihydrooroxylin A | STAT1 | 0.097874534 |
| dihydrooroxylin A | MET | 0.097874534 |
| dihydrooroxylin A | GUSB | 0.097874534 |
| dihydrooroxylin A | RXRA | 0.097874534 |
| dihydrooroxylin A | CA13 | 0.097874534 |
| dihydrooroxylin A | FFAR1 | 0.097874534 |
| dihydrooroxylin A | DAO | 0.097874534 |
| dihydrooroxylin A | MPI | 0.097874534 |
| dihydrooroxylin A | GSR | 0.097874534 |
| rivularin | PTGS2 | 0.164184052 |
| rivularin | CYP1B1 | 0.130706653 |
| rivularin | ABCG2 | 0.122339194 |
| rivularin | ADORA1 | 0.122339194 |
| rivularin | ADORA2A | 0.122339194 |
| rivularin | ODC1 | 0.113979815 |
| rivularin | AKR1B1 | 0.113979815 |
| rivularin | OPRM1 | 0.113979815 |
| rivularin | OPRD1 | 0.113979815 |
| rivularin | NOS2 | 0.10560828 |
| rivularin | MCL1 | 0.10560828 |
| rivularin | ALOX5 | 0.097239989 |
| rivularin | TERT | 0.097239989 |
| rivularin | KIT | 0.097239989 |
| rivularin | BCHE | 0.097239989 |
| rivularin | ADORA3 | 0.097239989 |
| rivularin | NOX4 | 0.097239989 |
| rivularin | ABCB1 | 0.097239989 |
| rivularin | MMP13 | 0.097239989 |
| rivularin | MMP9 | 0.097239989 |
| rivularin | FLT3 | 0.097239989 |
| rivularin | PTPRS | 0.097239989 |
| rivularin | ESR2 | 0.097239989 |
| rivularin | DAPK1 | 0.097239989 |
| rivularin | MPG | 0.097239989 |
| rivularin | CA2 | 0.097239989 |
| rivularin | APP | 0.097239989 |
| rivularin | MAOA | 0.097239989 |
| rivularin | MMP3 | 0.097239989 |
| rivularin | MMP2 | 0.097239989 |
| rivularin | HSD17B2 | 0.097239989 |
| rivularin | HSD17B1 | 0.097239989 |
| rivularin | EGFR | 0.097239989 |
| rivularin | KDR | 0.097239989 |
| rivularin | GPR35 | 0.097239989 |
| rivularin | AHR | 0.097239989 |
| rivularin | TYR | 0.097239989 |
| rivularin | CA1 | 0.097239989 |
| rivularin | PLG | 0.097239989 |
| rivularin | NTRK2 | 0.097239989 |
| rivularin | ALOX15 | 0.097239989 |
| rivularin | CA9 | 0.097239989 |
| rivularin | CYP19A1 | 0.097239989 |
| rivularin | GSK3B | 0.097239989 |
| rivularin | PLK1 | 0.097239989 |
| rivularin | MET | 0.097239989 |
| rivularin | XDH | 0.097239989 |
| rivularin | CA12 | 0.097239989 |
| rivularin | KDM4E | 0.097239989 |
| rivularin | TOP2A | 0.097239989 |
| rivularin | GLO1 | 0.097239989 |
| rivularin | CA3 | 0.097239989 |
| rivularin | PKN1 | 0.097239989 |
| rivularin | CAMK2B | 0.097239989 |
| rivularin | NEK6 | 0.097239989 |
| rivularin | PLA2G1B | 0.097239989 |
| rivularin | AXL | 0.097239989 |
| rivularin | APEX1 | 0.097239989 |
| rivularin | NUAK1 | 0.097239989 |
| rivularin | AKR1C2 | 0.097239989 |
| rivularin | AKR1C1 | 0.097239989 |
| rivularin | AKR1C3 | 0.097239989 |
| rivularin | AKR1C4 | 0.097239989 |
| rivularin | PIM1 | 0.097239989 |
| rivularin | CDK2 | 0.097239989 |
| rivularin | SRC | 0.097239989 |
| rivularin | PIK3CG | 0.097239989 |
| rivularin | ELANE | 0.097239989 |
| rivularin | CA7 | 0.097239989 |
| rivularin | NQO1 | 0.097239989 |
| rivularin | ACHE | 0.097239989 |
| rivularin | GUSB | 0.097239989 |
| rivularin | ALK | 0.097239989 |
| rivularin | MMP1 | 0.097239989 |
| rivularin | MMP16 | 0.097239989 |
| rivularin | MMP14 | 0.097239989 |
| rivularin | MMP8 | 0.097239989 |
| rivularin | MMP12 | 0.097239989 |
| rivularin | MPO | 0.097239989 |
| rivularin | CDK6 | 0.097239989 |
| rivularin | CBR1 | 0.097239989 |
| rivularin | ERN1 | 0.097239989 |
| rivularin | PDE4A | 0.097239989 |
| rivularin | PDE4C | 0.097239989 |
| rivularin | ADAM17 | 0.097239989 |
| rivularin | GRK6 | 0.097239989 |
| rivularin | FLT1 | 0.097239989 |
| rivularin | PDGFRB | 0.097239989 |
| rivularin | FLT4 | 0.097239989 |
| rivularin | TNKS | 0.097239989 |
| rivularin | BACE1 | 0.097239989 |
| rivularin | PLA2G7 | 0.097239989 |
| rivularin | SLC22A12 | 0.097239989 |
| rivularin | BMP1 | 0.097239989 |
| rivularin | AMY1A | 0.097239989 |
| rivularin | DUSP3 | 0.097239989 |
| rivularin | KCNMA1 | 0.097239989 |
| rivularin | CSNK2A1 | 0.097239989 |
| rivularin | MYLK | 0.097239989 |
| rivularin | PFKFB3 | 0.097239989 |
| 5,7,2',6'-Tetrahydroxyflavone | AKR1B1 | 0.328395696 |
| 5,7,2',6'-Tetrahydroxyflavone | ABCB1 | 0.303816095 |
| 5,7,2',6'-Tetrahydroxyflavone | XDH | 0.262581506 |
| 5,7,2',6'-Tetrahydroxyflavone | CA7 | 0.262581506 |
| 5,7,2',6'-Tetrahydroxyflavone | CA12 | 0.262581506 |
| 5,7,2',6'-Tetrahydroxyflavone | CA4 | 0.262581506 |
| 5,7,2',6'-Tetrahydroxyflavone | CYP1B1 | 0.262581506 |
| 5,7,2',6'-Tetrahydroxyflavone | ESR2 | 0.213125923 |
| 5,7,2',6'-Tetrahydroxyflavone | CA2 | 0.213125923 |
| 5,7,2',6'-Tetrahydroxyflavone | ABCG2 | 0.213125923 |
| 5,7,2',6'-Tetrahydroxyflavone | BCHE | 0.213125923 |
| 5,7,2',6'-Tetrahydroxyflavone | ACHE | 0.213125923 |
| 5,7,2',6'-Tetrahydroxyflavone | ADORA1 | 0.213125923 |
| 5,7,2',6'-Tetrahydroxyflavone | ADORA2A | 0.213125923 |
| 5,7,2',6'-Tetrahydroxyflavone | ADORA3 | 0.213125923 |
| 5,7,2',6'-Tetrahydroxyflavone | ESR1 | 0.196657136 |
| 5,7,2',6'-Tetrahydroxyflavone | CSNK2A1 | 0.196657136 |
| 5,7,2',6'-Tetrahydroxyflavone | CDK5R1 CDK5 | 0.196657136 |
| 5,7,2',6'-Tetrahydroxyflavone | CCNB3 CDK1 CCNB1 CCNB2 | 0.196657136 |
| 5,7,2',6'-Tetrahydroxyflavone | CDK6 | 0.188420386 |
| 5,7,2',6'-Tetrahydroxyflavone | PTPRS | 0.180252494 |
| 5,7,2',6'-Tetrahydroxyflavone | NOX4 | 0.163737226 |
| 5,7,2',6'-Tetrahydroxyflavone | FLT3 | 0.163737226 |
| 5,7,2',6'-Tetrahydroxyflavone | ABCC1 | 0.163737226 |
| 5,7,2',6'-Tetrahydroxyflavone | HSD17B1 | 0.163737226 |
| 5,7,2',6'-Tetrahydroxyflavone | MAOA | 0.155528102 |
| 5,7,2',6'-Tetrahydroxyflavone | SYK | 0.155528102 |
| 5,7,2',6'-Tetrahydroxyflavone | GSK3B | 0.155528102 |
| 5,7,2',6'-Tetrahydroxyflavone | TTR | 0.155528102 |
| 5,7,2',6'-Tetrahydroxyflavone | AKR1B10 | 0.155528102 |
| 5,7,2',6'-Tetrahydroxyflavone | TNKS2 | 0.155528102 |
| 5,7,2',6'-Tetrahydroxyflavone | TNKS | 0.155528102 |
| 5,7,2',6'-Tetrahydroxyflavone | CYP19A1 | 0.155528102 |
| 5,7,2',6'-Tetrahydroxyflavone | IGF1R | 0.155528102 |
| 5,7,2',6'-Tetrahydroxyflavone | PIM1 | 0.155528102 |
| 5,7,2',6'-Tetrahydroxyflavone | AURKB | 0.155528102 |
| 5,7,2',6'-Tetrahydroxyflavone | KDR | 0.155528102 |
| 5,7,2',6'-Tetrahydroxyflavone | PLK1 | 0.155528102 |
| 5,7,2',6'-Tetrahydroxyflavone | MET | 0.155528102 |
| 5,7,2',6'-Tetrahydroxyflavone | ALK | 0.155528102 |
| 5,7,2',6'-Tetrahydroxyflavone | AXL | 0.155528102 |
| 5,7,2',6'-Tetrahydroxyflavone | ALOX15 | 0.155528102 |
| 5,7,2',6'-Tetrahydroxyflavone | ALOX12 | 0.155528102 |
| 5,7,2',6'-Tetrahydroxyflavone | TYR | 0.147256737 |
| 5,7,2',6'-Tetrahydroxyflavone | ALOX5 | 0.147256737 |
| 5,7,2',6'-Tetrahydroxyflavone | HSD17B2 | 0.147256737 |
| 5,7,2',6'-Tetrahydroxyflavone | AHR | 0.147256737 |
| 5,7,2',6'-Tetrahydroxyflavone | ESRRA | 0.147256737 |
| 5,7,2',6'-Tetrahydroxyflavone | CA1 | 0.139061947 |
| 5,7,2',6'-Tetrahydroxyflavone | CA9 | 0.139061947 |
| 5,7,2',6'-Tetrahydroxyflavone | GPR35 | 0.139061947 |
| 5,7,2',6'-Tetrahydroxyflavone | DAPK1 | 0.139061947 |
| 5,7,2',6'-Tetrahydroxyflavone | MPG | 0.139061947 |
| 5,7,2',6'-Tetrahydroxyflavone | SLC22A12 | 0.139061947 |
| 5,7,2',6'-Tetrahydroxyflavone | PTGS2 | 0.130791955 |
| 5,7,2',6'-Tetrahydroxyflavone | CFTR | 0.130791955 |
| 5,7,2',6'-Tetrahydroxyflavone | CDK1 | 0.122581769 |
| 5,7,2',6'-Tetrahydroxyflavone | AR | 0.122581769 |
| 5,7,2',6'-Tetrahydroxyflavone | KDM4E | 0.122581769 |
| 5,7,2',6'-Tetrahydroxyflavone | GRK6 | 0.122581769 |
| 5,7,2',6'-Tetrahydroxyflavone | CBR1 | 0.122581769 |
| 5,7,2',6'-Tetrahydroxyflavone | PARP1 | 0.114337559 |
| 5,7,2',6'-Tetrahydroxyflavone | NAE1 | 0.114337559 |
| 5,7,2',6'-Tetrahydroxyflavone | GLO1 | 0.114337559 |
| 5,7,2',6'-Tetrahydroxyflavone | APP | 0.114337559 |
| 5,7,2',6'-Tetrahydroxyflavone | MMP9 | 0.114337559 |
| 5,7,2',6'-Tetrahydroxyflavone | MMP2 | 0.114337559 |
| 5,7,2',6'-Tetrahydroxyflavone | MMP12 | 0.114337559 |
| 5,7,2',6'-Tetrahydroxyflavone | CD38 | 0.114337559 |
| 5,7,2',6'-Tetrahydroxyflavone | TOP1 | 0.114337559 |
| 5,7,2',6'-Tetrahydroxyflavone | ARG1 | 0.114337559 |
| 5,7,2',6'-Tetrahydroxyflavone | LCK | 0.106099949 |
| 5,7,2',6'-Tetrahydroxyflavone | AMY1A | 0.106099949 |
| 5,7,2',6'-Tetrahydroxyflavone | CDK2 | 0.106099949 |
| 5,7,2',6'-Tetrahydroxyflavone | EGFR | 0.106099949 |
| 5,7,2',6'-Tetrahydroxyflavone | AVPR2 | 0.097874534 |
| 5,7,2',6'-Tetrahydroxyflavone | F2 | 0.097874534 |
| 5,7,2',6'-Tetrahydroxyflavone | DRD4 | 0.097874534 |
| 5,7,2',6'-Tetrahydroxyflavone | MPO | 0.097874534 |
| 5,7,2',6'-Tetrahydroxyflavone | PIK3R1 | 0.097874534 |
| 5,7,2',6'-Tetrahydroxyflavone | PYGL | 0.097874534 |
| 5,7,2',6'-Tetrahydroxyflavone | SRC | 0.097874534 |
| 5,7,2',6'-Tetrahydroxyflavone | PTK2 | 0.097874534 |
| 5,7,2',6'-Tetrahydroxyflavone | MMP13 | 0.097874534 |
| 5,7,2',6'-Tetrahydroxyflavone | MMP3 | 0.097874534 |
| 5,7,2',6'-Tetrahydroxyflavone | CA3 | 0.097874534 |
| 5,7,2',6'-Tetrahydroxyflavone | CA6 | 0.097874534 |
| 5,7,2',6'-Tetrahydroxyflavone | PKN1 | 0.097874534 |
| 5,7,2',6'-Tetrahydroxyflavone | CA14 | 0.097874534 |
| 5,7,2',6'-Tetrahydroxyflavone | NEK2 | 0.097874534 |
| 5,7,2',6'-Tetrahydroxyflavone | CXCR1 | 0.097874534 |
| 5,7,2',6'-Tetrahydroxyflavone | CAMK2B | 0.097874534 |
| 5,7,2',6'-Tetrahydroxyflavone | AKT1 | 0.097874534 |
| 5,7,2',6'-Tetrahydroxyflavone | NEK6 | 0.097874534 |
| 5,7,2',6'-Tetrahydroxyflavone | PLA2G1B | 0.097874534 |
| 5,7,2',6'-Tetrahydroxyflavone | CA5A | 0.097874534 |
| 5,7,2',6'-Tetrahydroxyflavone | BACE1 | 0.097874534 |
| 5,7,2',6'-Tetrahydroxyflavone | NUAK1 | 0.097874534 |
| 5,7,2',6'-Tetrahydroxyflavone | AKR1C2 | 0.097874534 |
| 5,7,2',6'-Tetrahydroxyflavone | AKR1C1 | 0.097874534 |
| 5,8,2'-Trihydroxy-7-methoxyflavone | AKR1B1 | 0.135202128 |
| 5,8,2'-Trihydroxy-7-methoxyflavone | MAOA | 0.12730257 |
| 5,8,2'-Trihydroxy-7-methoxyflavone | ABCB1 | 0.119403562 |
| 5,8,2'-Trihydroxy-7-methoxyflavone | GPR35 | 0.119403562 |
| 5,8,2'-Trihydroxy-7-methoxyflavone | ODC1 | 0.119403562 |
| 5,8,2'-Trihydroxy-7-methoxyflavone | CYP1B1 | 0.119403562 |
| 5,8,2'-Trihydroxy-7-methoxyflavone | ADORA1 | 0.119403562 |
| 5,8,2'-Trihydroxy-7-methoxyflavone | PTPRS | 0.119403562 |
| 5,8,2'-Trihydroxy-7-methoxyflavone | DAPK1 | 0.119403562 |
| 5,8,2'-Trihydroxy-7-methoxyflavone | MPG | 0.119403562 |
| 5,8,2'-Trihydroxy-7-methoxyflavone | SLC22A12 | 0.119403562 |
| 5,8,2'-Trihydroxy-7-methoxyflavone | PTGS2 | 0.119403562 |
| 5,8,2'-Trihydroxy-7-methoxyflavone | TERT | 0.119403562 |
| 5,8,2'-Trihydroxy-7-methoxyflavone | EGFR | 0.119403562 |
| 5,8,2'-Trihydroxy-7-methoxyflavone | KDR | 0.119403562 |
| 5,8,2'-Trihydroxy-7-methoxyflavone | BCHE | 0.111501865 |
| 5,8,2'-Trihydroxy-7-methoxyflavone | ESR2 | 0.111501865 |
| 5,8,2'-Trihydroxy-7-methoxyflavone | TYR | 0.111501865 |
| 5,8,2'-Trihydroxy-7-methoxyflavone | ABCC1 | 0.111501865 |
| 5,8,2'-Trihydroxy-7-methoxyflavone | AHR | 0.111501865 |
| 5,8,2'-Trihydroxy-7-methoxyflavone | ESRRA | 0.111501865 |
| 5,8,2'-Trihydroxy-7-methoxyflavone | CA2 | 0.111501865 |
| 5,8,2'-Trihydroxy-7-methoxyflavone | IKBKB | 0.111501865 |
| 5,8,2'-Trihydroxy-7-methoxyflavone | NTRK2 | 0.111501865 |
| 5,8,2'-Trihydroxy-7-methoxyflavone | ABCG2 | 0.111501865 |
| 5,8,2'-Trihydroxy-7-methoxyflavone | ALOX5 | 0.111501865 |
| 5,8,2'-Trihydroxy-7-methoxyflavone | ADORA2A | 0.111501865 |
| 5,8,2'-Trihydroxy-7-methoxyflavone | NOS2 | 0.111501865 |
| 5,8,2'-Trihydroxy-7-methoxyflavone | KIT | 0.111501865 |
| 5,8,2'-Trihydroxy-7-methoxyflavone | NOX4 | 0.111501865 |
| 5,8,2'-Trihydroxy-7-methoxyflavone | KDM4E | 0.111501865 |
| 5,8,2'-Trihydroxy-7-methoxyflavone | AVPR2 | 0.111501865 |
| 5,8,2'-Trihydroxy-7-methoxyflavone | TOP2A | 0.111501865 |
| 5,8,2'-Trihydroxy-7-methoxyflavone | DRD4 | 0.111501865 |
| 5,8,2'-Trihydroxy-7-methoxyflavone | GLO1 | 0.111501865 |
| 5,8,2'-Trihydroxy-7-methoxyflavone | PIK3R1 | 0.111501865 |
| 5,8,2'-Trihydroxy-7-methoxyflavone | PYGL | 0.111501865 |
| 5,8,2'-Trihydroxy-7-methoxyflavone | MMP3 | 0.111501865 |
| 5,8,2'-Trihydroxy-7-methoxyflavone | CA3 | 0.111501865 |
| 5,8,2'-Trihydroxy-7-methoxyflavone | CA6 | 0.111501865 |
| 5,8,2'-Trihydroxy-7-methoxyflavone | PKN1 | 0.111501865 |
| 5,8,2'-Trihydroxy-7-methoxyflavone | NEK2 | 0.111501865 |
| 5,8,2'-Trihydroxy-7-methoxyflavone | CAMK2B | 0.111501865 |
| 5,8,2'-Trihydroxy-7-methoxyflavone | NEK6 | 0.111501865 |
| 5,8,2'-Trihydroxy-7-methoxyflavone | PLA2G1B | 0.111501865 |
| 5,8,2'-Trihydroxy-7-methoxyflavone | CA5A | 0.111501865 |
| 5,8,2'-Trihydroxy-7-methoxyflavone | AXL | 0.111501865 |
| 5,8,2'-Trihydroxy-7-methoxyflavone | APEX1 | 0.111501865 |
| 5,8,2'-Trihydroxy-7-methoxyflavone | NUAK1 | 0.111501865 |
| 5,8,2'-Trihydroxy-7-methoxyflavone | AKR1C2 | 0.111501865 |
| 5,8,2'-Trihydroxy-7-methoxyflavone | AKR1C1 | 0.111501865 |
| 5,8,2'-Trihydroxy-7-methoxyflavone | AKR1C3 | 0.111501865 |
| 5,8,2'-Trihydroxy-7-methoxyflavone | AKR1C4 | 0.111501865 |
| 5,8,2'-Trihydroxy-7-methoxyflavone | AKR1A1 | 0.111501865 |
| 5,8,2'-Trihydroxy-7-methoxyflavone | ALOX15 | 0.111501865 |
| 5,8,2'-Trihydroxy-7-methoxyflavone | CA12 | 0.111501865 |
| 5,8,2'-Trihydroxy-7-methoxyflavone | HSD17B2 | 0.111501865 |
| 5,8,2'-Trihydroxy-7-methoxyflavone | HSD17B1 | 0.111501865 |
| 5,8,2'-Trihydroxy-7-methoxyflavone | CA1 | 0.111501865 |
| 5,8,2'-Trihydroxy-7-methoxyflavone | MMP9 | 0.111501865 |
| 5,8,2'-Trihydroxy-7-methoxyflavone | MMP2 | 0.111501865 |
| 5,8,2'-Trihydroxy-7-methoxyflavone | CYP1A1 | 0.111501865 |
| 5,8,2'-Trihydroxy-7-methoxyflavone | CYP1A2 | 0.111501865 |
| 5,8,2'-Trihydroxy-7-methoxyflavone | OPRM1 | 0.111501865 |
| 5,8,2'-Trihydroxy-7-methoxyflavone | OPRD1 | 0.111501865 |
| 5,8,2'-Trihydroxy-7-methoxyflavone | MMP13 | 0.111501865 |
| 5,8,2'-Trihydroxy-7-methoxyflavone | SRC | 0.111501865 |
| 5,8,2'-Trihydroxy-7-methoxyflavone | PIK3CG | 0.111501865 |
| 5,8,2'-Trihydroxy-7-methoxyflavone | MCL1 | 0.111501865 |
| 5,8,2'-Trihydroxy-7-methoxyflavone | CA7 | 0.111501865 |
| 5,8,2'-Trihydroxy-7-methoxyflavone | CDK6 | 0.111501865 |
| 5,8,2'-Trihydroxy-7-methoxyflavone | CBR1 | 0.111501865 |
| 5,8,2'-Trihydroxy-7-methoxyflavone | FLT3 | 0.111501865 |
| 5,8,2'-Trihydroxy-7-methoxyflavone | GRK6 | 0.111501865 |
| 5,8,2'-Trihydroxy-7-methoxyflavone | CSNK2A1 | 0.111501865 |
| 5,8,2'-Trihydroxy-7-methoxyflavone | AMY1A | 0.111501865 |
| 5,8,2'-Trihydroxy-7-methoxyflavone | CA4 | 0.111501865 |
| 5,8,2'-Trihydroxy-7-methoxyflavone | BACE1 | 0.111501865 |
| 5,8,2'-Trihydroxy-7-methoxyflavone | CA9 | 0.111501865 |
| 5,8,2'-Trihydroxy-7-methoxyflavone | MET | 0.111501865 |
| 5,8,2'-Trihydroxy-7-methoxyflavone | CYP19A1 | 0.111501865 |
| 5,8,2'-Trihydroxy-7-methoxyflavone | ARG1 | 0.111501865 |
| 5,8,2'-Trihydroxy-7-methoxyflavone | GSK3B | 0.111501865 |
| 5,8,2'-Trihydroxy-7-methoxyflavone | XDH | 0.111501865 |
| 5,8,2'-Trihydroxy-7-methoxyflavone | CFTR | 0.111501865 |
| 5,8,2'-Trihydroxy-7-methoxyflavone | AKR1B10 | 0.111501865 |
| 5,8,2'-Trihydroxy-7-methoxyflavone | NAE1 | 0.111501865 |
| 5,8,2'-Trihydroxy-7-methoxyflavone | ACHE | 0.111501865 |
| 5,8,2'-Trihydroxy-7-methoxyflavone | AR | 0.111501865 |
| 5,8,2'-Trihydroxy-7-methoxyflavone | IGF1R | 0.111501865 |
| 5,8,2'-Trihydroxy-7-methoxyflavone | TNKS | 0.111501865 |
| 5,8,2'-Trihydroxy-7-methoxyflavone | SYK | 0.111501865 |
| 5,8,2'-Trihydroxy-7-methoxyflavone | ADORA3 | 0.111501865 |
| 5,8,2'-Trihydroxy-7-methoxyflavone | CD38 | 0.111501865 |
| 5,8,2'-Trihydroxy-7-methoxyflavone | F2 | 0.111501865 |
| 5,8,2'-Trihydroxy-7-methoxyflavone | CDK5R1 CDK5 | 0.111501865 |
| 5,8,2'-Trihydroxy-7-methoxyflavone | CCNB3 CDK1 CCNB1 CCNB2 | 0.111501865 |
| 5,8,2'-Trihydroxy-7-methoxyflavone | PFKFB3 | 0.111501865 |
| 5,8,2'-Trihydroxy-7-methoxyflavone | NQO1 | 0.111501865 |
| 5,8,2'-Trihydroxy-7-methoxyflavone | CA14 | 0.111501865 |
| beta-sitosterol | Q9UHC9 | 0.747494949 |
| beta-sitosterol | Q13133 | 0.689284537 |
| beta-sitosterol | P51449 | 0.581021535 |
| beta-sitosterol | P04278 | 0.45601846 |
| beta-sitosterol | P04035 | 0.414383452 |
| beta-sitosterol | Q12772 | 0.347726199 |
| beta-sitosterol | P11511 | 0.314405075 |
| beta-sitosterol | P05093 | 0.264448566 |
| beta-sitosterol | P10275 | 0.256112183 |
| beta-sitosterol | Q16850 | 0.247762388 |
| beta-sitosterol | P35398 | 0.206119055 |
| beta-sitosterol | P03372 | 0.181090099 |
| beta-sitosterol | Q92731 | 0.181090099 |
| beta-sitosterol | P18031 | 0.139453236 |
| beta-sitosterol | P33261 | 0.131155749 |
| beta-sitosterol | P08185 | 0.11449479 |
| beta-sitosterol | P11413 | 0.11449479 |
| beta-sitosterol | P22303 | 0.11449479 |
| beta-sitosterol | P23975 | 0.11449479 |
| beta-sitosterol | P31645 | 0.11449479 |
| beta-sitosterol | P06276 | 0.106165761 |
| beta-sitosterol | Q14994 | 0.106165761 |
| beta-sitosterol | P08172 | 0.106165761 |
| beta-sitosterol | P55055 | 0.106165761 |
| beta-sitosterol | P11473 | 0.106165761 |
| beta-sitosterol | P34995 | 0.106165761 |
| beta-sitosterol | P43116 | 0.106165761 |
| beta-sitosterol | P24557 | 0.106165761 |
| beta-sitosterol | O14684 | 0.106165761 |
| beta-sitosterol | P23415 | 0.106165761 |
| beta-sitosterol | Q07869 | 0.106165761 |
| beta-sitosterol | Q03181 | 0.106165761 |
| beta-sitosterol | Q9UBM7 | 0.106165761 |
| beta-sitosterol | Q14534 | 0.106165761 |
| beta-sitosterol | P29350 | 0.106165761 |
| beta-sitosterol | P17706 | 0.106165761 |
| beta-sitosterol | O00748 | 0.106165761 |
| beta-sitosterol | P37268 | 0.106165761 |
| beta-sitosterol | P35228 | 0.106165761 |
| beta-sitosterol | P28845 | 0.106165761 |
| beta-sitosterol | P37231 | 0.106165761 |
| beta-sitosterol | P16662 | 0.106165761 |
| beta-sitosterol | P06746 | 0.106165761 |
| sitosterol | Q9UHC9 | 0.747494949 |
| sitosterol | Q13133 | 0.689284537 |
| sitosterol | P51449 | 0.581021535 |
| sitosterol | P04278 | 0.45601846 |
| sitosterol | P04035 | 0.414383452 |
| sitosterol | Q12772 | 0.347726199 |
| sitosterol | P11511 | 0.314405075 |
| sitosterol | P05093 | 0.264448566 |
| sitosterol | P10275 | 0.256112183 |
| sitosterol | Q16850 | 0.247762388 |
| sitosterol | P35398 | 0.206119055 |
| sitosterol | P03372 | 0.181090099 |
| sitosterol | Q92731 | 0.181090099 |
| sitosterol | P18031 | 0.139453236 |
| sitosterol | P33261 | 0.131155749 |
| sitosterol | P08185 | 0.11449479 |
| sitosterol | P11413 | 0.11449479 |
| sitosterol | P22303 | 0.11449479 |
| sitosterol | P23975 | 0.11449479 |
| sitosterol | P31645 | 0.11449479 |
| sitosterol | P06276 | 0.106165761 |
| sitosterol | Q14994 | 0.106165761 |
| sitosterol | P08172 | 0.106165761 |
| sitosterol | P55055 | 0.106165761 |
| sitosterol | P11473 | 0.106165761 |
| sitosterol | P34995 | 0.106165761 |
| sitosterol | P43116 | 0.106165761 |
| sitosterol | P24557 | 0.106165761 |
| sitosterol | O14684 | 0.106165761 |
| sitosterol | P23415 | 0.106165761 |
| sitosterol | Q07869 | 0.106165761 |
| sitosterol | Q03181 | 0.106165761 |
| sitosterol | Q9UBM7 | 0.106165761 |
| sitosterol | Q14534 | 0.106165761 |
| sitosterol | P29350 | 0.106165761 |
| sitosterol | P17706 | 0.106165761 |
| sitosterol | O00748 | 0.106165761 |
| sitosterol | P37268 | 0.106165761 |
| sitosterol | P35228 | 0.106165761 |
| sitosterol | P28845 | 0.106165761 |
| sitosterol | P37231 | 0.106165761 |
| sitosterol | P16662 | 0.106165761 |
| sitosterol | P06746 | 0.106165761 |
| 5,7,4'-trihydroxy-6-methoxyflavanone | TAS2R31 | 0.403756445 |
| 5,7,4'-trihydroxy-6-methoxyflavanone | CYP1B1 | 0.158886034 |
| 5,7,4'-trihydroxy-6-methoxyflavanone | ABCG2 | 0.143102156 |
| 5,7,4'-trihydroxy-6-methoxyflavanone | CA7 | 0.143102156 |
| 5,7,4'-trihydroxy-6-methoxyflavanone | MAOB | 0.143102156 |
| 5,7,4'-trihydroxy-6-methoxyflavanone | CA12 | 0.143102156 |
| 5,7,4'-trihydroxy-6-methoxyflavanone | CA4 | 0.143102156 |
| 5,7,4'-trihydroxy-6-methoxyflavanone | ADORA1 | 0.143102156 |
| 5,7,4'-trihydroxy-6-methoxyflavanone | ADORA3 | 0.143102156 |
| 5,7,4'-trihydroxy-6-methoxyflavanone | SHBG | 0.135202128 |
| 5,7,4'-trihydroxy-6-methoxyflavanone | CBR1 | 0.135202128 |
| 5,7,4'-trihydroxy-6-methoxyflavanone | ESR2 | 0.135202128 |
| 5,7,4'-trihydroxy-6-methoxyflavanone | HSD17B1 | 0.135202128 |
| 5,7,4'-trihydroxy-6-methoxyflavanone | CYP19A1 | 0.12730257 |
| 5,7,4'-trihydroxy-6-methoxyflavanone | ESR1 | 0.119403562 |
| 5,7,4'-trihydroxy-6-methoxyflavanone | ABCC1 | 0.119403562 |
| 5,7,4'-trihydroxy-6-methoxyflavanone | MMP13 | 0.111501865 |
| 5,7,4'-trihydroxy-6-methoxyflavanone | MMP12 | 0.111501865 |
| 5,7,4'-trihydroxy-6-methoxyflavanone | PTGS1 | 0.111501865 |
| 5,7,4'-trihydroxy-6-methoxyflavanone | PLA2G1B | 0.111501865 |
| 5,7,4'-trihydroxy-6-methoxyflavanone | CA3 | 0.111501865 |
| 5,7,4'-trihydroxy-6-methoxyflavanone | CES1 | 0.111501865 |
| 5,7,4'-trihydroxy-6-methoxyflavanone | CES2 | 0.111501865 |
| 5,7,4'-trihydroxy-6-methoxyflavanone | KLK1 | 0.111501865 |
| 5,7,4'-trihydroxy-6-methoxyflavanone | KLK2 | 0.111501865 |
| 5,7,4'-trihydroxy-6-methoxyflavanone | BACE1 | 0.111501865 |
| 5,7,4'-trihydroxy-6-methoxyflavanone | CA2 | 0.111501865 |
| 5,7,4'-trihydroxy-6-methoxyflavanone | CA1 | 0.111501865 |
| 5,7,4'-trihydroxy-6-methoxyflavanone | STS | 0.111501865 |
| 5,7,4'-trihydroxy-6-methoxyflavanone | POLB | 0.111501865 |
| 5,7,4'-trihydroxy-6-methoxyflavanone | MMP14 | 0.111501865 |
| 5,7,4'-trihydroxy-6-methoxyflavanone | PLA2G5 | 0.111501865 |
| 5,7,4'-trihydroxy-6-methoxyflavanone | PLA2G10 | 0.111501865 |
| 5,7,4'-trihydroxy-6-methoxyflavanone | MET | 0.111501865 |
| 5,7,4'-trihydroxy-6-methoxyflavanone | GRM2 | 0.111501865 |
| 5,7,4'-trihydroxy-6-methoxyflavanone | KDM1A | 0.111501865 |
| 5,7,4'-trihydroxy-6-methoxyflavanone | SGK1 | 0.111501865 |
| 5,7,4'-trihydroxy-6-methoxyflavanone | ACHE | 0.111501865 |
| 5,7,4'-trihydroxy-6-methoxyflavanone | MMP2 | 0.111501865 |
| 5,7,4'-trihydroxy-6-methoxyflavanone | MAPT | 0.111501865 |
| 5,7,4'-trihydroxy-6-methoxyflavanone | DNMT1 | 0.111501865 |
| 5,7,4'-trihydroxy-6-methoxyflavanone | PGD | 0.111501865 |
| 5,7,4'-trihydroxy-6-methoxyflavanone | ST3GAL3 | 0.111501865 |
| 5,7,4'-trihydroxy-6-methoxyflavanone | FUT7 | 0.111501865 |
| 5,7,4'-trihydroxy-6-methoxyflavanone | FUT4 | 0.111501865 |
| 5,7,4'-trihydroxy-6-methoxyflavanone | STAT1 | 0.111501865 |
| 5,7,4'-trihydroxy-6-methoxyflavanone | SQLE | 0.111501865 |
| 5,7,4'-trihydroxy-6-methoxyflavanone | PLG | 0.111501865 |
| 5,7,4'-trihydroxy-6-methoxyflavanone | MAOA | 0.111501865 |
| 5,7,4'-trihydroxy-6-methoxyflavanone | CA6 | 0.111501865 |
| 5,7,4'-trihydroxy-6-methoxyflavanone | CA5A | 0.111501865 |
| 5,7,4'-trihydroxy-6-methoxyflavanone | HPGDS | 0.111501865 |
| 5,7,4'-trihydroxy-6-methoxyflavanone | CA13 | 0.111501865 |
| 5,7,4'-trihydroxy-6-methoxyflavanone | DNM1 | 0.111501865 |
| 5,7,4'-trihydroxy-6-methoxyflavanone | ADORA2A | 0.111501865 |
| 5,7,4'-trihydroxy-6-methoxyflavanone | CA5B | 0.111501865 |
| 5,7,4'-trihydroxy-6-methoxyflavanone | SPHK2 | 0.111501865 |
| 5,7,4'-trihydroxy-6-methoxyflavanone | SPHK1 | 0.111501865 |
| 5,7,4'-trihydroxy-6-methoxyflavanone | KDR | 0.111501865 |
| 5,7,4'-trihydroxy-6-methoxyflavanone | RXRA | 0.111501865 |
| 5,7,4'-trihydroxy-6-methoxyflavanone | CHRNA7 | 0.111501865 |
| 5,7,4'-trihydroxy-6-methoxyflavanone | BCHE | 0.111501865 |
| 5,7,4'-trihydroxy-6-methoxyflavanone | SLC5A2 | 0.111501865 |
| 5,7,4'-trihydroxy-6-methoxyflavanone | KIT | 0.111501865 |
| 5,7,4'-trihydroxy-6-methoxyflavanone | APP | 0.111501865 |
| 5,7,4'-trihydroxy-6-methoxyflavanone | DYRK1A | 0.111501865 |
| 5,7,4'-trihydroxy-6-methoxyflavanone | NOX4 | 0.111501865 |
| 5,7,4'-trihydroxy-6-methoxyflavanone | MAPK14 | 0.111501865 |
| 5,7,4'-trihydroxy-6-methoxyflavanone | TERT | 0.111501865 |
| 5,7,4'-trihydroxy-6-methoxyflavanone | CA9 | 0.111501865 |
| 5,7,4'-Trihydroxy-8-methoxyflavone | AKR1B1 | 0.609130548 |
| 5,7,4'-Trihydroxy-8-methoxyflavone | NOS2 | 0.348497063 |
| 5,7,4'-Trihydroxy-8-methoxyflavone | PTGS2 | 0.277371341 |
| 5,7,4'-Trihydroxy-8-methoxyflavone | PIM1 | 0.222013735 |
| 5,7,4'-Trihydroxy-8-methoxyflavone | ADORA1 | 0.222013735 |
| 5,7,4'-Trihydroxy-8-methoxyflavone | ADORA2A | 0.222013735 |
| 5,7,4'-Trihydroxy-8-methoxyflavone | ESR2 | 0.158886034 |
| 5,7,4'-Trihydroxy-8-methoxyflavone | OPRD1 | 0.15098181 |
| 5,7,4'-Trihydroxy-8-methoxyflavone | TERT | 0.143102156 |
| 5,7,4'-Trihydroxy-8-methoxyflavone | MCL1 | 0.143102156 |
| 5,7,4'-Trihydroxy-8-methoxyflavone | ESR1 | 0.143102156 |
| 5,7,4'-Trihydroxy-8-methoxyflavone | EGFR | 0.135202128 |
| 5,7,4'-Trihydroxy-8-methoxyflavone | HSD17B2 | 0.135202128 |
| 5,7,4'-Trihydroxy-8-methoxyflavone | HSD17B1 | 0.135202128 |
| 5,7,4'-Trihydroxy-8-methoxyflavone | PFKFB3 | 0.135202128 |
| 5,7,4'-Trihydroxy-8-methoxyflavone | APP | 0.12730257 |
| 5,7,4'-Trihydroxy-8-methoxyflavone | OPRM1 | 0.12730257 |
| 5,7,4'-Trihydroxy-8-methoxyflavone | FLT3 | 0.12730257 |
| 5,7,4'-Trihydroxy-8-methoxyflavone | PTPRS | 0.12730257 |
| 5,7,4'-Trihydroxy-8-methoxyflavone | ABCG2 | 0.119403562 |
| 5,7,4'-Trihydroxy-8-methoxyflavone | AMY1A | 0.119403562 |
| 5,7,4'-Trihydroxy-8-methoxyflavone | GRK6 | 0.119403562 |
| 5,7,4'-Trihydroxy-8-methoxyflavone | CYP1B1 | 0.119403562 |
| 5,7,4'-Trihydroxy-8-methoxyflavone | KIT | 0.119403562 |
| 5,7,4'-Trihydroxy-8-methoxyflavone | ALOX5 | 0.119403562 |
| 5,7,4'-Trihydroxy-8-methoxyflavone | ABCB1 | 0.119403562 |
| 5,7,4'-Trihydroxy-8-methoxyflavone | SLC22A12 | 0.119403562 |
| 5,7,4'-Trihydroxy-8-methoxyflavone | NOX4 | 0.119403562 |
| 5,7,4'-Trihydroxy-8-methoxyflavone | IGF1R | 0.119403562 |
| 5,7,4'-Trihydroxy-8-methoxyflavone | XDH | 0.119403562 |
| 5,7,4'-Trihydroxy-8-methoxyflavone | ABCC1 | 0.119403562 |
| 5,7,4'-Trihydroxy-8-methoxyflavone | CYP1A1 | 0.111501865 |
| 5,7,4'-Trihydroxy-8-methoxyflavone | CYP1A2 | 0.111501865 |
| 5,7,4'-Trihydroxy-8-methoxyflavone | KDM4E | 0.111501865 |
| 5,7,4'-Trihydroxy-8-methoxyflavone | ALOX15 | 0.111501865 |
| 5,7,4'-Trihydroxy-8-methoxyflavone | CDK1 | 0.111501865 |
| 5,7,4'-Trihydroxy-8-methoxyflavone | ALOX12 | 0.111501865 |
| 5,7,4'-Trihydroxy-8-methoxyflavone | CYP19A1 | 0.111501865 |
| 5,7,4'-Trihydroxy-8-methoxyflavone | CA2 | 0.111501865 |
| 5,7,4'-Trihydroxy-8-methoxyflavone | CA12 | 0.111501865 |
| 5,7,4'-Trihydroxy-8-methoxyflavone | TYR | 0.111501865 |
| 5,7,4'-Trihydroxy-8-methoxyflavone | CA7 | 0.111501865 |
| 5,7,4'-Trihydroxy-8-methoxyflavone | AHR | 0.111501865 |
| 5,7,4'-Trihydroxy-8-methoxyflavone | ESRRA | 0.111501865 |
| 5,7,4'-Trihydroxy-8-methoxyflavone | CA4 | 0.111501865 |
| 5,7,4'-Trihydroxy-8-methoxyflavone | CDK5R1 CDK5 | 0.111501865 |
| 5,7,4'-Trihydroxy-8-methoxyflavone | PIK3CG | 0.111501865 |
| 5,7,4'-Trihydroxy-8-methoxyflavone | IKBKB | 0.111501865 |
| 5,7,4'-Trihydroxy-8-methoxyflavone | NTRK2 | 0.111501865 |
| 5,7,4'-Trihydroxy-8-methoxyflavone | TNKS2 | 0.111501865 |
| 5,7,4'-Trihydroxy-8-methoxyflavone | TNKS | 0.111501865 |
| 5,7,4'-Trihydroxy-8-methoxyflavone | MAOA | 0.111501865 |
| 5,7,4'-Trihydroxy-8-methoxyflavone | CCNB3 CDK1 CCNB1 CCNB2 | 0.111501865 |
| 5,7,4'-Trihydroxy-8-methoxyflavone | ACHE | 0.111501865 |
| 5,7,4'-Trihydroxy-8-methoxyflavone | CDK6 | 0.111501865 |
| 5,7,4'-Trihydroxy-8-methoxyflavone | SYK | 0.111501865 |
| 5,7,4'-Trihydroxy-8-methoxyflavone | GSK3B | 0.111501865 |
| 5,7,4'-Trihydroxy-8-methoxyflavone | TTR | 0.111501865 |
| 5,7,4'-Trihydroxy-8-methoxyflavone | CSNK2A1 | 0.111501865 |
| 5,7,4'-Trihydroxy-8-methoxyflavone | CFTR | 0.111501865 |
| 5,7,4'-Trihydroxy-8-methoxyflavone | AKR1B10 | 0.111501865 |
| 5,7,4'-Trihydroxy-8-methoxyflavone | AR | 0.111501865 |
| 5,7,4'-Trihydroxy-8-methoxyflavone | ADORA3 | 0.111501865 |
| 5,7,4'-Trihydroxy-8-methoxyflavone | BACE1 | 0.111501865 |
| 5,7,4'-Trihydroxy-8-methoxyflavone | CBR1 | 0.111501865 |
| 5,7,4'-Trihydroxy-8-methoxyflavone | CA1 | 0.111501865 |
| 5,7,4'-Trihydroxy-8-methoxyflavone | CA9 | 0.111501865 |
| 5,7,4'-Trihydroxy-8-methoxyflavone | PLA2G2A | 0.111501865 |
| 5,7,4'-Trihydroxy-8-methoxyflavone | GPR35 | 0.111501865 |
| 5,7,4'-Trihydroxy-8-methoxyflavone | DAPK1 | 0.111501865 |
| 5,7,4'-Trihydroxy-8-methoxyflavone | MPG | 0.111501865 |
| 5,7,4'-Trihydroxy-8-methoxyflavone | SRC | 0.111501865 |
| 5,7,4'-Trihydroxy-8-methoxyflavone | MET | 0.111501865 |
| 5,7,4'-Trihydroxy-8-methoxyflavone | F2 | 0.111501865 |
| 5,7,4'-Trihydroxy-8-methoxyflavone | PARP1 | 0.111501865 |
| 5,7,4'-Trihydroxy-8-methoxyflavone | NAE1 | 0.111501865 |
| 5,7,4'-Trihydroxy-8-methoxyflavone | ARG1 | 0.111501865 |
| 5,7,4'-Trihydroxy-8-methoxyflavone | AURKB | 0.111501865 |
| 5,7,4'-Trihydroxy-8-methoxyflavone | GLO1 | 0.111501865 |
| 5,7,4'-Trihydroxy-8-methoxyflavone | MMP9 | 0.111501865 |
| 5,7,4'-Trihydroxy-8-methoxyflavone | MMP2 | 0.111501865 |
| 5,7,4'-Trihydroxy-8-methoxyflavone | MMP12 | 0.111501865 |
| 5,7,4'-Trihydroxy-8-methoxyflavone | CD38 | 0.111501865 |
| 5,7,4'-Trihydroxy-8-methoxyflavone | TOP1 | 0.111501865 |
| 5,7,4'-Trihydroxy-8-methoxyflavone | PLG | 0.111501865 |
| 5,7,4'-Trihydroxy-8-methoxyflavone | ODC1 | 0.111501865 |
| 5,7,4'-Trihydroxy-8-methoxyflavone | AVPR2 | 0.111501865 |
| 5,7,4'-Trihydroxy-8-methoxyflavone | DRD4 | 0.111501865 |
| 5,7,4'-Trihydroxy-8-methoxyflavone | MPO | 0.111501865 |
| 5,7,4'-Trihydroxy-8-methoxyflavone | PIK3R1 | 0.111501865 |
| 5,7,4'-Trihydroxy-8-methoxyflavone | PYGL | 0.111501865 |
| 5,7,4'-Trihydroxy-8-methoxyflavone | PTK2 | 0.111501865 |
| 5,7,4'-Trihydroxy-8-methoxyflavone | KDR | 0.111501865 |
| 5,7,4'-Trihydroxy-8-methoxyflavone | MMP13 | 0.111501865 |
| 5,7,4'-Trihydroxy-8-methoxyflavone | MMP3 | 0.111501865 |
| 5,7,4'-Trihydroxy-8-methoxyflavone | CA3 | 0.111501865 |
| 5,7,4'-Trihydroxy-8-methoxyflavone | PLK1 | 0.111501865 |
| 5,7,4'-Trihydroxy-8-methoxyflavone | CA6 | 0.111501865 |
| 5,7,4'-Trihydroxy-8-methoxyflavone | PKN1 | 0.111501865 |
| 5,7,4'-Trihydroxy-8-methoxyflavone | CA14 | 0.111501865 |
| acacetin | CYP1B1 | 1 |
| acacetin | CYP19A1 | 0.353074081 |
| acacetin | CA7 | 0.254289912 |
| acacetin | CA12 | 0.254289912 |
| acacetin | CA4 | 0.254289912 |
| acacetin | CBR1 | 0.254289912 |
| acacetin | ABCC1 | 0.237885168 |
| acacetin | ESR2 | 0.213125923 |
| acacetin | ABCB1 | 0.213125923 |
| acacetin | NOX4 | 0.188420386 |
| acacetin | TNKS2 | 0.180252494 |
| acacetin | TNKS | 0.180252494 |
| acacetin | CDK5R1 CDK5 | 0.171978589 |
| acacetin | XDH | 0.171978589 |
| acacetin | CCNB3 CDK1 CCNB1 CCNB2 | 0.171978589 |
| acacetin | ACHE | 0.171978589 |
| acacetin | CDK6 | 0.171978589 |
| acacetin | ESR1 | 0.171978589 |
| acacetin | AKR1B1 | 0.163737226 |
| acacetin | FLT3 | 0.163737226 |
| acacetin | HSD17B1 | 0.163737226 |
| acacetin | ABCG2 | 0.163737226 |
| acacetin | MAOA | 0.155528102 |
| acacetin | ADORA1 | 0.155528102 |
| acacetin | ADORA2A | 0.155528102 |
| acacetin | SYK | 0.155528102 |
| acacetin | GSK3B | 0.155528102 |
| acacetin | TTR | 0.155528102 |
| acacetin | AKR1B10 | 0.155528102 |
| acacetin | KIT | 0.147256737 |
| acacetin | OPRD1 | 0.147256737 |
| acacetin | LCK | 0.147256737 |
| acacetin | HSD17B2 | 0.147256737 |
| acacetin | ALOX15 | 0.139061947 |
| acacetin | ALOX12 | 0.139061947 |
| acacetin | PTGS2 | 0.139061947 |
| acacetin | CSNK2A1 | 0.139061947 |
| acacetin | CFTR | 0.139061947 |
| acacetin | PIM1 | 0.130791955 |
| acacetin | CA2 | 0.130791955 |
| acacetin | MCL1 | 0.130791955 |
| acacetin | PTPRS | 0.130791955 |
| acacetin | PLG | 0.122581769 |
| acacetin | ARG1 | 0.122581769 |
| acacetin | ALOX5 | 0.114337559 |
| acacetin | CA1 | 0.114337559 |
| acacetin | CA9 | 0.114337559 |
| acacetin | GRK6 | 0.114337559 |
| acacetin | APP | 0.114337559 |
| acacetin | GLO1 | 0.114337559 |
| acacetin | PARP1 | 0.114337559 |
| acacetin | MMP9 | 0.114337559 |
| acacetin | MMP2 | 0.114337559 |
| acacetin | MMP12 | 0.114337559 |
| acacetin | CD38 | 0.114337559 |
| acacetin | TOP1 | 0.114337559 |
| acacetin | AMY1A | 0.106099949 |
| acacetin | PLA2G2A | 0.106099949 |
| acacetin | NAE1 | 0.106099949 |
| acacetin | CDK1 | 0.106099949 |
| acacetin | KDM4E | 0.106099949 |
| acacetin | TYR | 0.106099949 |
| acacetin | AHR | 0.106099949 |
| acacetin | ESRRA | 0.106099949 |
| acacetin | TERT | 0.106099949 |
| acacetin | ADORA3 | 0.106099949 |
| acacetin | MAOB | 0.097874534 |
| acacetin | PFKFB3 | 0.097874534 |
| acacetin | SLC22A12 | 0.097874534 |
| acacetin | AR | 0.097874534 |
| acacetin | ST6GAL1 | 0.097874534 |
| acacetin | SIGMAR1 | 0.097874534 |
| acacetin | IGF1R | 0.097874534 |
| acacetin | EGFR | 0.097874534 |
| acacetin | NOS2 | 0.097874534 |
| acacetin | BACE1 | 0.097874534 |
| acacetin | CALM1 | 0.097874534 |
| acacetin | CYP1A1 | 0.097874534 |
| acacetin | CYP1A2 | 0.097874534 |
| acacetin | F2 | 0.097874534 |
| acacetin | DAPK1 | 0.097874534 |
| acacetin | CDK2 | 0.097874534 |
| acacetin | GPR35 | 0.097874534 |
| acacetin | MPG | 0.097874534 |
| acacetin | AVPR2 | 0.097874534 |
| acacetin | AURKB | 0.097874534 |
| acacetin | DRD4 | 0.097874534 |
| acacetin | MPO | 0.097874534 |
| acacetin | PIK3R1 | 0.097874534 |
| acacetin | PYGL | 0.097874534 |
| acacetin | SRC | 0.097874534 |
| acacetin | PTK2 | 0.097874534 |
| acacetin | KDR | 0.097874534 |
| acacetin | MMP13 | 0.097874534 |
| acacetin | MMP3 | 0.097874534 |
| acacetin | CA3 | 0.097874534 |
| acacetin | PLK1 | 0.097874534 |
| acacetin | CA6 | 0.097874534 |
| acacetin | PKN1 | 0.097874534 |
| acacetin | CA14 | 0.097874534 |
| Supraene | PPARA | 0.106165761 |
| Supraene | CNR2 | 0.106165761 |
| baicalein | KDM4E | 1 |
| baicalein | XDH | 1 |
| baicalein | ALOX15 | 1 |
| baicalein | CDK1 | 1 |
| baicalein | ALOX12 | 1 |
| baicalein | GRK6 | 1 |
| baicalein | CYP19A1 | 0.436430316 |
| baicalein | CA7 | 0.346270483 |
| baicalein | CA12 | 0.346270483 |
| baicalein | CA4 | 0.346270483 |
| baicalein | ABCB1 | 0.346270483 |
| baicalein | CYP1B1 | 0.346270483 |
| baicalein | HSD17B1 | 0.33811031 |
| baicalein | AKR1B1 | 0.329868485 |
| baicalein | CDK5R1 CDK5 | 0.329868485 |
| baicalein | CA2 | 0.329868485 |
| baicalein | CCNB3 CDK1 CCNB1 CCNB2 | 0.329868485 |
| baicalein | CDK6 | 0.329868485 |
| baicalein | CA1 | 0.329868485 |
| baicalein | CA9 | 0.329868485 |
| baicalein | ABCG2 | 0.329868485 |
| baicalein | CBR1 | 0.329868485 |
| baicalein | ESR2 | 0.264392858 |
| baicalein | ESR1 | 0.25622784 |
| baicalein | ACHE | 0.247942823 |
| baicalein | ADORA1 | 0.247942823 |
| baicalein | ADORA2A | 0.247942823 |
| baicalein | PTGS2 | 0.247942823 |
| baicalein | PTPRS | 0.239574696 |
| baicalein | AMY1A | 0.239574696 |
| baicalein | FLT3 | 0.231637185 |
| baicalein | IKBKB | 0.215238178 |
| baicalein | NTRK2 | 0.215238178 |
| baicalein | AR | 0.182484829 |
| baicalein | NOX4 | 0.174270753 |
| baicalein | MAOA | 0.174270753 |
| baicalein | SYK | 0.174270753 |
| baicalein | GSK3B | 0.174270753 |
| baicalein | ABCC1 | 0.174270753 |
| baicalein | TTR | 0.174270753 |
| baicalein | CSNK2A1 | 0.174270753 |
| baicalein | CFTR | 0.174270753 |
| baicalein | AKR1B10 | 0.174270753 |
| baicalein | TNKS2 | 0.174270753 |
| baicalein | TNKS | 0.174270753 |
| baicalein | HSD17B2 | 0.157929217 |
| baicalein | BCHE | 0.157929217 |
| baicalein | ADORA3 | 0.157929217 |
| baicalein | TERT | 0.149732594 |
| baicalein | LCK | 0.149732594 |
| baicalein | PFKFB3 | 0.141522086 |
| baicalein | PIM1 | 0.141522086 |
| baicalein | PARP1 | 0.125142649 |
| baicalein | ALOX5 | 0.125142649 |
| baicalein | APP | 0.125142649 |
| baicalein | CALM1 | 0.125142649 |
| baicalein | ARG1 | 0.125142649 |
| baicalein | NOS2 | 0.116965063 |
| baicalein | GLO1 | 0.116965063 |
| baicalein | MMP9 | 0.116965063 |
| baicalein | MMP2 | 0.116965063 |
| baicalein | MMP12 | 0.116965063 |
| baicalein | CD38 | 0.116965063 |
| baicalein | TOP1 | 0.116965063 |
| baicalein | NAE1 | 0.108770969 |
| baicalein | EGFR | 0.108770969 |
| baicalein | SRC | 0.108770969 |
| baicalein | TYR | 0.108770969 |
| baicalein | AHR | 0.108770969 |
| baicalein | ESRRA | 0.108770969 |
| baicalein | KIT | 0.108770969 |
| baicalein | OPRD1 | 0.108770969 |
| baicalein | CYP1A1 | 0.108770969 |
| baicalein | AURKB | 0.108770969 |
| baicalein | PIK3CG | 0.108770969 |
| baicalein | MAPT | 0.108770969 |
| baicalein | TOP2A | 0.108770969 |
| baicalein | INSR | 0.108770969 |
| baicalein | MYLK | 0.108770969 |
| baicalein | APEX1 | 0.108770969 |
| baicalein | IGF1R | 0.108770969 |
| baicalein | KDR | 0.100578902 |
| baicalein | PLK1 | 0.100578902 |
| baicalein | MET | 0.100578902 |
| baicalein | ALK | 0.100578902 |
| baicalein | AXL | 0.100578902 |
| baicalein | PTPN1 | 0.100578902 |
| baicalein | ST6GAL1 | 0.100578902 |
| baicalein | SLC22A12 | 0.100578902 |
| baicalein | GPR35 | 0.100578902 |
| baicalein | FYN | 0.100578902 |
| baicalein | TACR2 | 0.100578902 |
| baicalein | PRKDC | 0.100578902 |
| baicalein | MAPK3 | 0.100578902 |
| baicalein | BACE1 | 0.100578902 |
| baicalein | SIRT1 | 0.100578902 |
| baicalein | PLA2G2A | 0.100578902 |
| baicalein | PLA2G4A | 0.100578902 |
| baicalein | CA6 | 0.100578902 |
| baicalein | CDK2 | 0.100578902 |
| 5,2'-Dihydroxy-6,7,8-trimethoxyflavone | KIT | 0.348262677 |
| 5,2'-Dihydroxy-6,7,8-trimethoxyflavone | AKR1B1 | 0.256161866 |
| 5,2'-Dihydroxy-6,7,8-trimethoxyflavone | CYP1B1 | 0.139088623 |
| 5,2'-Dihydroxy-6,7,8-trimethoxyflavone | ADORA1 | 0.122339194 |
| 5,2'-Dihydroxy-6,7,8-trimethoxyflavone | ADORA2A | 0.122339194 |
| 5,2'-Dihydroxy-6,7,8-trimethoxyflavone | OPRM1 | 0.113979815 |
| 5,2'-Dihydroxy-6,7,8-trimethoxyflavone | PTGS2 | 0.113979815 |
| 5,2'-Dihydroxy-6,7,8-trimethoxyflavone | ODC1 | 0.113979815 |
| 5,2'-Dihydroxy-6,7,8-trimethoxyflavone | OPRD1 | 0.113979815 |
| 5,2'-Dihydroxy-6,7,8-trimethoxyflavone | ABCG2 | 0.10560828 |
| 5,2'-Dihydroxy-6,7,8-trimethoxyflavone | ADORA3 | 0.10560828 |
| 5,2'-Dihydroxy-6,7,8-trimethoxyflavone | ABCB1 | 0.10560828 |
| 5,2'-Dihydroxy-6,7,8-trimethoxyflavone | MCL1 | 0.10560828 |
| 5,2'-Dihydroxy-6,7,8-trimethoxyflavone | NOS2 | 0.097239989 |
| 5,2'-Dihydroxy-6,7,8-trimethoxyflavone | GPR35 | 0.097239989 |
| 5,2'-Dihydroxy-6,7,8-trimethoxyflavone | ALOX5 | 0.097239989 |
| 5,2'-Dihydroxy-6,7,8-trimethoxyflavone | PLG | 0.097239989 |
| 5,2'-Dihydroxy-6,7,8-trimethoxyflavone | ESR2 | 0.097239989 |
| 5,2'-Dihydroxy-6,7,8-trimethoxyflavone | HSD17B2 | 0.097239989 |
| 5,2'-Dihydroxy-6,7,8-trimethoxyflavone | HSD17B1 | 0.097239989 |
| 5,2'-Dihydroxy-6,7,8-trimethoxyflavone | PTPRS | 0.097239989 |
| 5,2'-Dihydroxy-6,7,8-trimethoxyflavone | MPG | 0.097239989 |
| 5,2'-Dihydroxy-6,7,8-trimethoxyflavone | SLC22A12 | 0.097239989 |
| 5,2'-Dihydroxy-6,7,8-trimethoxyflavone | BCHE | 0.097239989 |
| 5,2'-Dihydroxy-6,7,8-trimethoxyflavone | MMP13 | 0.097239989 |
| 5,2'-Dihydroxy-6,7,8-trimethoxyflavone | MMP9 | 0.097239989 |
| 5,2'-Dihydroxy-6,7,8-trimethoxyflavone | MMP2 | 0.097239989 |
| 5,2'-Dihydroxy-6,7,8-trimethoxyflavone | TERT | 0.097239989 |
| 5,2'-Dihydroxy-6,7,8-trimethoxyflavone | CYP1A2 | 0.097239989 |
| 5,2'-Dihydroxy-6,7,8-trimethoxyflavone | FLT3 | 0.097239989 |
| 5,2'-Dihydroxy-6,7,8-trimethoxyflavone | CA2 | 0.097239989 |
| 5,2'-Dihydroxy-6,7,8-trimethoxyflavone | AHR | 0.097239989 |
| 5,2'-Dihydroxy-6,7,8-trimethoxyflavone | ESRRA | 0.097239989 |
| 5,2'-Dihydroxy-6,7,8-trimethoxyflavone | ABCC1 | 0.097239989 |
| 5,2'-Dihydroxy-6,7,8-trimethoxyflavone | NOX4 | 0.097239989 |
| 5,2'-Dihydroxy-6,7,8-trimethoxyflavone | MAOA | 0.097239989 |
| 5,2'-Dihydroxy-6,7,8-trimethoxyflavone | MMP3 | 0.097239989 |
| 5,2'-Dihydroxy-6,7,8-trimethoxyflavone | PIM1 | 0.097239989 |
| 5,2'-Dihydroxy-6,7,8-trimethoxyflavone | CA7 | 0.097239989 |
| 5,2'-Dihydroxy-6,7,8-trimethoxyflavone | CA12 | 0.097239989 |
| 5,2'-Dihydroxy-6,7,8-trimethoxyflavone | EGFR | 0.097239989 |
| 5,2'-Dihydroxy-6,7,8-trimethoxyflavone | KDR | 0.097239989 |
| 5,2'-Dihydroxy-6,7,8-trimethoxyflavone | PLK1 | 0.097239989 |
| 5,2'-Dihydroxy-6,7,8-trimethoxyflavone | CCNB3 CDK1 CCNB1 CCNB2 | 0.097239989 |
| 5,2'-Dihydroxy-6,7,8-trimethoxyflavone | MAPT | 0.097239989 |
| 5,2'-Dihydroxy-6,7,8-trimethoxyflavone | AVPR2 | 0.097239989 |
| 5,2'-Dihydroxy-6,7,8-trimethoxyflavone | TOP2A | 0.097239989 |
| 5,2'-Dihydroxy-6,7,8-trimethoxyflavone | GLO1 | 0.097239989 |
| 5,2'-Dihydroxy-6,7,8-trimethoxyflavone | CA1 | 0.097239989 |
| 5,2'-Dihydroxy-6,7,8-trimethoxyflavone | CA3 | 0.097239989 |
| 5,2'-Dihydroxy-6,7,8-trimethoxyflavone | CA9 | 0.097239989 |
| 5,2'-Dihydroxy-6,7,8-trimethoxyflavone | CAMK2B | 0.097239989 |
| 5,2'-Dihydroxy-6,7,8-trimethoxyflavone | PLA2G1B | 0.097239989 |
| 5,2'-Dihydroxy-6,7,8-trimethoxyflavone | APEX1 | 0.097239989 |
| 5,2'-Dihydroxy-6,7,8-trimethoxyflavone | AKR1C2 | 0.097239989 |
| 5,2'-Dihydroxy-6,7,8-trimethoxyflavone | AKR1C1 | 0.097239989 |
| 5,2'-Dihydroxy-6,7,8-trimethoxyflavone | AKR1C3 | 0.097239989 |
| 5,2'-Dihydroxy-6,7,8-trimethoxyflavone | AKR1C4 | 0.097239989 |
| 5,2'-Dihydroxy-6,7,8-trimethoxyflavone | SRC | 0.097239989 |
| 5,2'-Dihydroxy-6,7,8-trimethoxyflavone | PIK3CG | 0.097239989 |
| 5,2'-Dihydroxy-6,7,8-trimethoxyflavone | TYR | 0.097239989 |
| 5,2'-Dihydroxy-6,7,8-trimethoxyflavone | PLA2G7 | 0.097239989 |
| 5,2'-Dihydroxy-6,7,8-trimethoxyflavone | PFKFB3 | 0.097239989 |
| 5,2'-Dihydroxy-6,7,8-trimethoxyflavone | ACHE | 0.097239989 |
| 5,2'-Dihydroxy-6,7,8-trimethoxyflavone | CYP19A1 | 0.097239989 |
| 5,2'-Dihydroxy-6,7,8-trimethoxyflavone | ALOX15 | 0.097239989 |
| 5,2'-Dihydroxy-6,7,8-trimethoxyflavone | XDH | 0.097239989 |
| 5,2'-Dihydroxy-6,7,8-trimethoxyflavone | APP | 0.097239989 |
| 5,2'-Dihydroxy-6,7,8-trimethoxyflavone | DAPK1 | 0.097239989 |
| 5,2'-Dihydroxy-6,7,8-trimethoxyflavone | AMY1A | 0.097239989 |
| 5,2'-Dihydroxy-6,7,8-trimethoxyflavone | GSK3B | 0.097239989 |
| 5,2'-Dihydroxy-6,7,8-trimethoxyflavone | MMP1 | 0.097239989 |
| 5,2'-Dihydroxy-6,7,8-trimethoxyflavone | ADAM17 | 0.097239989 |
| 5,2'-Dihydroxy-6,7,8-trimethoxyflavone | MMP7 | 0.097239989 |
| 5,2'-Dihydroxy-6,7,8-trimethoxyflavone | MMP8 | 0.097239989 |
| 5,2'-Dihydroxy-6,7,8-trimethoxyflavone | AURKB | 0.097239989 |
| 5,2'-Dihydroxy-6,7,8-trimethoxyflavone | SYK | 0.097239989 |
| 5,2'-Dihydroxy-6,7,8-trimethoxyflavone | CA13 | 0.097239989 |
| 5,2'-Dihydroxy-6,7,8-trimethoxyflavone | CDK6 | 0.097239989 |
| 5,2'-Dihydroxy-6,7,8-trimethoxyflavone | ARG1 | 0.097239989 |
| 5,2'-Dihydroxy-6,7,8-trimethoxyflavone | MET | 0.097239989 |
| 5,2'-Dihydroxy-6,7,8-trimethoxyflavone | NQO1 | 0.097239989 |
| 5,2'-Dihydroxy-6,7,8-trimethoxyflavone | CDK1 | 0.097239989 |
| 5,2'-Dihydroxy-6,7,8-trimethoxyflavone | CSNK2A1 | 0.097239989 |
| 5,2'-Dihydroxy-6,7,8-trimethoxyflavone | GUSB | 0.097239989 |
| 5,2'-Dihydroxy-6,7,8-trimethoxyflavone | ALK | 0.097239989 |
| 5,2'-Dihydroxy-6,7,8-trimethoxyflavone | CA4 | 0.097239989 |
| 5,2'-Dihydroxy-6,7,8-trimethoxyflavone | TNKS | 0.097239989 |
| 5,2'-Dihydroxy-6,7,8-trimethoxyflavone | RET | 0.097239989 |
| 5,2'-Dihydroxy-6,7,8-trimethoxyflavone | HSP90AB1 | 0.097239989 |
| 5,2'-Dihydroxy-6,7,8-trimethoxyflavone | BACE1 | 0.097239989 |
| 5,2'-Dihydroxy-6,7,8-trimethoxyflavone | MPO | 0.097239989 |
| 5,2'-Dihydroxy-6,7,8-trimethoxyflavone | PCSK7 | 0.097239989 |
| 5,2'-Dihydroxy-6,7,8-trimethoxyflavone | CYP2C9 | 0.097239989 |
| 5,2'-Dihydroxy-6,7,8-trimethoxyflavone | NAE1 | 0.097239989 |
| 5,2'-Dihydroxy-6,7,8-trimethoxyflavone | PDGFRB | 0.097239989 |
| 5,2'-Dihydroxy-6,7,8-trimethoxyflavone | GRK6 | 0.097239989 |
| 5,2'-Dihydroxy-6,7,8-trimethoxyflavone | FNTA FNTB | 0.097239989 |
| 5,2'-Dihydroxy-6,7,8-trimethoxyflavone | PTK2 | 0.097239989 |
| 5,2'-Dihydroxy-6,7,8-trimethoxyflavone | CDK5R1 CDK5 | 0.097239989 |
| wogonin | PTGS2 | 1 |
| wogonin | NOS2 | 1 |
| wogonin | FLT3 | 0.246150751 |
| wogonin | AKR1B1 | 0.229685699 |
| wogonin | OPRD1 | 0.139061947 |
| wogonin | KIT | 0.130791955 |
| wogonin | ABCB1 | 0.130791955 |
| wogonin | IKBKB | 0.122581769 |
| wogonin | NTRK2 | 0.122581769 |
| wogonin | KDM4E | 0.106099949 |
| wogonin | XDH | 0.106099949 |
| wogonin | ALOX15 | 0.106099949 |
| wogonin | CDK1 | 0.106099949 |
| wogonin | ALOX12 | 0.106099949 |
| wogonin | GRK6 | 0.106099949 |
| wogonin | CYP19A1 | 0.106099949 |
| wogonin | ESR2 | 0.106099949 |
| wogonin | CYP1A1 | 0.106099949 |
| wogonin | OPRM1 | 0.106099949 |
| wogonin | ABCG2 | 0.106099949 |
| wogonin | ESR1 | 0.106099949 |
| wogonin | EGFR | 0.106099949 |
| wogonin | SLC22A12 | 0.106099949 |
| wogonin | CYP1B1 | 0.106099949 |
| wogonin | CA4 | 0.106099949 |
| wogonin | CA2 | 0.097874534 |
| wogonin | CA1 | 0.097874534 |
| wogonin | MCL1 | 0.097874534 |
| wogonin | PIK3CG | 0.097874534 |
| wogonin | PIM1 | 0.097874534 |
| wogonin | ADORA1 | 0.097874534 |
| wogonin | ADORA2A | 0.097874534 |
| wogonin | HSD17B2 | 0.097874534 |
| wogonin | HSD17B1 | 0.097874534 |
| wogonin | CA7 | 0.097874534 |
| wogonin | CA12 | 0.097874534 |
| wogonin | CDK5R1 CDK5 | 0.097874534 |
| wogonin | CCNB3 CDK1 CCNB1 CCNB2 | 0.097874534 |
| wogonin | CDK6 | 0.097874534 |
| wogonin | CA9 | 0.097874534 |
| wogonin | CBR1 | 0.097874534 |
| wogonin | TERT | 0.097874534 |
| wogonin | AR | 0.097874534 |
| wogonin | CA6 | 0.097874534 |
| wogonin | PTPRS | 0.097874534 |
| wogonin | DAPK1 | 0.097874534 |
| wogonin | MPG | 0.097874534 |
| wogonin | PFKFB3 | 0.097874534 |
| wogonin | MMP9 | 0.097874534 |
| wogonin | MMP2 | 0.097874534 |
| wogonin | LCK | 0.097874534 |
| wogonin | MMP12 | 0.097874534 |
| wogonin | CD38 | 0.097874534 |
| wogonin | TOP1 | 0.097874534 |
| wogonin | ARG1 | 0.097874534 |
| wogonin | MAPT | 0.097874534 |
| wogonin | TOP2A | 0.097874534 |
| wogonin | INSR | 0.097874534 |
| wogonin | DRD4 | 0.097874534 |
| wogonin | MYLK | 0.097874534 |
| wogonin | MPO | 0.097874534 |
| wogonin | PIK3R1 | 0.097874534 |
| wogonin | PYGL | 0.097874534 |
| wogonin | MMP13 | 0.097874534 |
| wogonin | MMP3 | 0.097874534 |
| wogonin | CA3 | 0.097874534 |
| wogonin | CA14 | 0.097874534 |
| wogonin | MET | 0.097874534 |
| wogonin | CA13 | 0.097874534 |
| wogonin | CAMK2B | 0.097874534 |
| wogonin | PLA2G1B | 0.097874534 |
| wogonin | CA5A | 0.097874534 |
| wogonin | APEX1 | 0.097874534 |
| wogonin | AKR1C2 | 0.097874534 |
| wogonin | AKR1C1 | 0.097874534 |
| wogonin | AKR1C3 | 0.097874534 |
| wogonin | AKR1C4 | 0.097874534 |
| wogonin | AKR1A1 | 0.097874534 |
| wogonin | GPR35 | 0.097874534 |
| wogonin | ODC1 | 0.097874534 |
| wogonin | HSP90AA1 | 0.097874534 |
| wogonin | ALOX5 | 0.097874534 |
| wogonin | CXCR1 | 0.097874534 |
| wogonin | PLA2G2A | 0.097874534 |
| wogonin | SRC | 0.097874534 |
| wogonin | APP | 0.097874534 |
| wogonin | CYP1A2 | 0.097874534 |
| wogonin | ADORA3 | 0.097874534 |
| wogonin | HSP90B1 | 0.097874534 |
| wogonin | ABCC1 | 0.097874534 |
| wogonin | TNKS | 0.097874534 |
| wogonin | TTR | 0.097874534 |
| wogonin | NOX4 | 0.097874534 |
| wogonin | AVPR2 | 0.097874534 |
| wogonin | NAE1 | 0.097874534 |
| wogonin | BCHE | 0.097874534 |
| wogonin | ACHE | 0.097874534 |
| wogonin | BACE1 | 0.097874534 |
| wogonin | PDE5A | 0.097874534 |
| wogonin | GSK3B | 0.097874534 |
| coptisine | ACHE | 0.306568978 |
| coptisine | SIGMAR1 | 0.241979077 |
| coptisine | CHRM1 | 0.217731049 |
| coptisine | HTR2B | 0.209643327 |
| coptisine | BCHE | 0.209643327 |
| coptisine | ADRA2C | 0.209643327 |
| coptisine | ADRA2B | 0.209643327 |
| coptisine | CYP2D6 | 0.209643327 |
| coptisine | SAE1 UBA2 | 0.201584698 |
| coptisine | RAC1 | 0.104671941 |
| coptisine | CDC42 | 0.104671941 |
| coptisine | CHRM4 | 0.104671941 |
| coptisine | TBXAS1 | 0.104671941 |
| coptisine | XBP1 | 0.104671941 |
| coptisine | IKBKB | 0.104671941 |
| coptisine | PLK1 | 0.104671941 |
| coptisine | PRF1 | 0.104671941 |
| coptisine | ABL1 | 0.104671941 |
| coptisine | CHEK2 | 0.104671941 |
| coptisine | SCN9A | 0.104671941 |
| coptisine | CDC7 | 0.104671941 |
| coptisine | LIMK1 | 0.104671941 |
| coptisine | SCD | 0.104671941 |
| Astragaloside Ⅳ, | PSEN2 PSENEN NCSTN APH1A PSEN1 APH1B | 0.100110094 |
| Astragaloside Ⅳ, | HSP90AA1 | 0.050560032 |
| Astragaloside Ⅳ, | VEGFA | 0.050560032 |
| Astragaloside Ⅳ, | FGF1 | 0.050560032 |
| Astragaloside Ⅳ, | FGF2 | 0.050560032 |
| Astragaloside Ⅳ, | HPSE | 0.050560032 |
| Astragaloside Ⅳ, | LGALS4 | 0.040647891 |
| Astragaloside Ⅳ, | LGALS3 | 0.040647891 |
| Astragaloside Ⅳ, | LGALS8 | 0.040647891 |
| Astragaloside Ⅳ, | HTR2B | 0.040647891 |
| Astragaloside Ⅳ, | ADRA2A | 0.040647891 |
| Astragaloside Ⅳ, | ADRA2C | 0.040647891 |
| Astragaloside Ⅳ, | ADRA2B | 0.040647891 |
| Astragaloside Ⅳ, | DRD1 | 0.040647891 |
| Astragaloside Ⅳ, | ADRA1D | 0.040647891 |
| Astragaloside Ⅳ, | CYP2D6 | 0.040647891 |
| Astragaloside Ⅳ, | HTR6 | 0.040647891 |
| Astragaloside Ⅳ, | ADRA1A | 0.040647891 |
| Astragaloside Ⅳ, | HTR1B | 0.040647891 |
| Astragaloside Ⅳ, | PTPRA | 0.040647891 |
| Astragaloside Ⅳ, | PTPN2 | 0.040647891 |
| Astragaloside Ⅳ, | CDK1 | 0.040647891 |
| Astragaloside Ⅳ, | AKT2 | 0.040647891 |
| Astragaloside Ⅳ, | RPS6KA1 | 0.040647891 |
| Astragaloside Ⅳ, | ROCK1 | 0.040647891 |
| Astragaloside Ⅳ, | AKT1 | 0.040647891 |
| Astragaloside Ⅳ, | STAT3 | 0.040647891 |
| Astragaloside Ⅳ, | MET | 0.040647891 |
| calycosin-7-O-β-D-glucoside | TNF | 0.999640903 |
| calycosin-7-O-β-D-glucoside | IL2 | 0.999640903 |
| calycosin-7-O-β-D-glucoside | ALDH2 | 0.148023744 |
| calycosin-7-O-β-D-glucoside | ADORA1 | 0.106542926 |
| calycosin-7-O-β-D-glucoside | AKR1B1 | 0.106542926 |
| calycosin-7-O-β-D-glucoside | XDH | 0.106542926 |
| calycosin-7-O-β-D-glucoside | CA1 | 0.106542926 |
| calycosin-7-O-β-D-glucoside | CA13 | 0.106542926 |
| calycosin-7-O-β-D-glucoside | CA12 | 0.106542926 |
| calycosin-7-O-β-D-glucoside | CA9 | 0.106542926 |
| calycosin-7-O-β-D-glucoside | TOP1 | 0.106542926 |
| calycosin-7-O-β-D-glucoside | ADORA3 | 0.106542926 |
| calycosin-7-O-β-D-glucoside | DRD4 | 0.106542926 |
| calycosin-7-O-β-D-glucoside | HSP90AA1 | 0.106542926 |
| calycosin-7-O-β-D-glucoside | CA2 | 0.106542926 |
